# Prediction of Drug–Drug–Gene Interaction Scenarios of (*E*)-Clomiphene and Its Metabolites Using Physiologically Based Pharmacokinetic Modeling

**DOI:** 10.3390/pharmaceutics14122604

**Published:** 2022-11-25

**Authors:** Christina Kovar, Lukas Kovar, Simeon Rüdesheim, Dominik Selzer, Boian Ganchev, Patrick Kröner, Svitlana Igel, Reinhold Kerb, Elke Schaeffeler, Thomas E. Mürdter, Matthias Schwab, Thorsten Lehr

**Affiliations:** 1 Clinical Pharmacy, Saarland University, 66123 Saarbrücken, Germany; 2 Dr. Margarete Fischer-Bosch Institute of Clinical Pharmacology, University of Tübingen, 70376 Stuttgart, Germany; 3 Departments of Clinical Pharmacology, Pharmacy and Biochemistry, University of Tübingen, 72076 Tübingen, Germany

**Keywords:** clomiphene, pharmacokinetics, cytochrome P450 2D6 (CYP2D6) polymorphisms, drug–drug interactions (DDIs), drug–drug–gene interactions (DDGIs), drug–gene interactions (DGIs), (*E*)-clomiphene, physiologically based pharmacokinetic (PBPK) modeling

## Abstract

Clomiphene, a selective estrogen receptor modulator (SERM), has been used for the treatment of anovulation for more than 50 years. However, since (*E*)-clomiphene ((*E*)-Clom) and its metabolites are eliminated primarily via Cytochrome P450 (CYP) 2D6 and CYP3A4, exposure can be affected by CYP2D6 polymorphisms and concomitant use with CYP inhibitors. Thus, clomiphene therapy may be susceptible to drug–gene interactions (DGIs), drug–drug interactions (DDIs) and drug–drug–gene interactions (DDGIs). Physiologically based pharmacokinetic (PBPK) modeling is a tool to quantify such DGI and DD(G)I scenarios. This study aimed to develop a whole-body PBPK model of (*E*)-Clom including three important metabolites to describe and predict DGI and DD(G)I effects. Model performance was evaluated both graphically and by calculating quantitative measures. Here, 90% of predicted C_max_ and 80% of AUC_last_ values were within two-fold of the corresponding observed value for DGIs and DD(G)Is with clarithromycin and paroxetine. The model also revealed quantitative contributions of different CYP enzymes to the involved metabolic pathways of (*E*)-Clom and its metabolites. The developed PBPK model can be employed to assess the exposure of (*E*)-Clom and its active metabolites in as-yet unexplored DD(G)I scenarios in future studies.

## 1. Introduction

Ovulation disorders resulting in infertility can be caused by polycystic ovary syndrome (PCOS), which shows a prevalence of 4–20% in women of reproductive age worldwide [1,2]. Clomiphene has been used for the treatment of infertility in women with PCOS since the late 1960s and is administered orally as a racemic mixture of (*E*)- and (*Z*)-clomiphene ((*E*)-Clom and (*Z*)-Clom) [1,3]. As a selective estrogen receptor modulator (SERM), clomiphene—particularly (*E*)-Clom and its metabolites—inhibits the estrogen receptor at the hypothalamic arcuate nucleus [4,5,6]. Here, a rise in gonadotropin-releasing hormone levels leads to an increase in follicle-stimulating and luteinizing hormones, which in turn, induces ovulation [7]. In addition, antimicrobial activity of SERMs against different strains of bacteria has been shown in recent work [8,9].

During clomiphene therapy, 8–54% of women do not respond, while variability in response is affected by various factors such as hyperandrogenemia and obesity [10,11,12]. Additionally, research efforts have identified the importance of the highly polymorphic cytochrome P450 (CYP) 2D6 enzyme in the bioactivation of (*E*)-Clom [6,13]. Here, the two metabolites (*E*)-4-hydroxyclomiphene ((*E*)-4-OH-Clom) and (*E*)-4-hydroxy-N-desethylclomiphene ((*E*)-4-OH-DE-Clom) were identified to exhibit the highest inhibitory affinity towards the estrogen receptor with half-maximal inhibitory concentrations of 2.2 and 0.9 nM, respectively [7]. In contrast, the parent drug (*E*)-Clom as well as (*Z*)-Clom and its metabolites showed lower inhibitory effects in in vitro assays [5,6]. Thus, (*E*)-4-OH-Clom and (*E*)-4-OH-DE-Clom are assumed to be key components in the bioactivation process of clomiphene with their pharmacokinetics (PK) strongly depending on CYP2D6 activity [5].

As a result, treatment with clomiphene can be subject to drug–gene interactions (DGIs) which has been confirmed in a study with healthy female volunteers [5]. Here, CYP2D6 poor metabolizers (PM) showed approximately ten-fold lower maximum plasma concentrations (C_max_) of (*E*)-4-OH-Clom and (*E*)-4-OH-DE-Clom compared with normal metabolizers (NM) [5]. Furthermore, the in vitro formation rates for both (*E*)-4-OH-Clom and (*E*)-4-OH-DE-Clom increased with CYP2D6 activity [5]. The impact of CYP2D6 polymorphisms has also been observed in a recent clinical trial, where all CYP2D6 intermediate metabolizers (IM) responded to clomiphene therapy, while 30% of NM were non-responders [14]. However, this non-classical gene–dose effect points to a more complex metabolic scheme.

As the biotransformation of its active metabolites does not only depend on CYP2D6, but also on CYP3A4 metabolism, among others, systemic exposure of (*E*)-Clom and its metabolites can be altered by drug–drug interactions (DDIs) with CYP2D6 inhibitors and additionally with CYP3A4 inhibitors/inducers [15,16]. This dependency of (*E*)-Clom PK and bioactivation on CYP2D6 and CYP3A4 leads to a complex network of possible DGI, DDI and drug–drug–gene interaction (DDGI) scenarios that can cause a high variability in the longitudinal trajectory of plasma concentrations for (*E*)-Clom and its metabolites. The fact, that not only the formation, but also the elimination, of the active metabolites depends on CYP2D6 and CYP3A4 activity, adds to the complexity of the PK. Here, physiologically based pharmacokinetic (PBPK) modeling can integrate available in vitro and in vivo information on these processes to quantify and investigate DGI, DDI and DDGI scenarios.

Thus, this study aimed to develop a whole-body parent–metabolite PBPK model of (*E*)-Clom and its metabolites (*E*)-4-OH-Clom, (*E*)-N-desethylclomiphene ((*E*)-DE-Clom) and (*E*)-4-OH-DE-Clom to support the investigation of CYP2D6 DGI effects on the PK and bioactivation of (*E*)-Clom. In addition, the model was applied to predict various DD(G)I scenarios with the CYP2D6 inhibitor paroxetine and the CYP3A4 inhibitor clarithromycin and to gain insights into the PK regarding the contribution of different metabolic pathways to the elimination of (*E*)-Clom and its metabolites. The supplementary document to this article serves as a model reference and includes a detailed evaluation of the model performance. In addition, the model files will be made publicly available (http://models.clinicalpharmacy.me/).

## 2. Materials and Methods

### 2.1. Clinical Study Data

Clinical data from a recently performed pharmacokinetic panel study (EudraCT-Nr.: 2009-014531-20, ClinicalTrails.gov: NCT01289756) were used for PBPK model development [6]. The study protocol, patient information sheet and consent form were approved by the Ethics Committee of the University of Tübingen and the German Federal Institute for Drugs and Medical Devices (BfArM). All study participants had signed an informed consent form.

**Figure 1:**
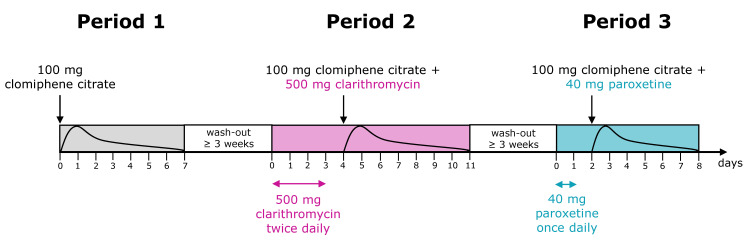
Drug administration schedule in the pharmacokinetic panel study. In period I, clomiphene citrate alone; in period II, combined with clarithromycin; and in period III, combined with paroxetine was administered.

The study was conducted in 20 healthy, Caucasian, premenopausal female volunteers that were genotyped for CYP2D6 polymorphisms and subsequently assigned to predicted phenotypes according to the respective CYP2D6 activity score (AS) as depicted in Table 1 [17,18]. All subjects received 100 mg clomiphene citrate (two 50 mg tablets Ratiopharm GmbH, Ulm, Germany, with 62:38 (*E*)-Clom:(*Z*)-Clom) as a single dose after an overnight fast and without any concomitant medication. After a wash-out phase of at least three weeks, clomiphene was administered concomitantly with the strong CYP3A4 inhibitor clarithromycin [19]. Here, the participants received 500 mg clarithromycin twice daily for four days. On day 5, a single dose of clomiphene citrate was administered together with 500 mg clarithromycin. Finally, in the third period, all subjects received clomiphene citrate together with the strong CYP2D6 inhibitor paroxetine [19]. Here, 40 mg paroxetine was administered once daily for two days. On day 3, participants received a single dose of clomiphene citrate concomitantly with 40 mg paroxetine (Figure 1).

Both plasma concentration–time profiles as well as renal excretion data of (*E*)-Clom and its metabolites (*E*)-4-OH-Clom, (*E*)-DE-Clom and (*E*)-4-OH-DE-Clom were obtained by validated liquid chromatography–tandem mass spectrometry (LC-MS/MS) methods [13,20]. The demographic and clinical characteristics of the study population are shown in Table 1.

Additionally, (*E*)-Clom plasma concentration–time profiles from two single-dose [21,22] and two multiple-dose [23,24] studies were identified in a literature search and plasma profiles were digitized for further model evaluation. In these clinical trials, *CYP2D6* genotypes of study participants were not reported. Additional information including study populations and the corresponding administration protocols are listed in Table S2 of the supplementary document.

### 2.2. Software

PBPK modeling and simulation was performed in PK-Sim^®^ and MoBi^®^ (version 9.1 part of the Open Systems Pharmacology (OSP) Suite, http://www.open-systems-pharmacology.org) [25]. Published clinical data of (*E*)-Clom were digitized with GetData Graph Digitizer version 2.26.0.20 (S. Fedorov) according to Wojtyniak and coworkers [26]. PK parameter calculations, model performance evaluations and graphics were accomplished with the R programming language version 3.6.3 (R Foundation for Statistical Computing, Vienna, Austria) [27]. Model parameter estimation via Monte-Carlo optimization as well as local sensitivity analysis were performed within PK-Sim^®^.

### 2.3. PBPK Model Development

For PBPK model building, information on physicochemical properties, as well as absorption, distribution, metabolism and excretion (ADME) processes of all investigated compounds, were gathered from the literature. Clinical data were split into a training and a test dataset. The training dataset for model development comprised mean plasma and renal excretion profiles of (*E*)-Clom and its metabolites from NM and PM study populations (*n* = 8 plasma concentration–time profiles and *n* = 8 renal excretion profiles). This dataset was selected to inform catalytic rate constant (k_cat_) parameters associated with CYP2D6-dependent and -independent metabolic pathways, respectively. Plasma concentration–time profiles and renal excretion data of IM and ultrarapid metabolizers (UM) in the DGI setting, data from all phenotypes in the DD(G)I setting as well as digitized clinical study data from the published literature were utilized as the test dataset for PBPK model evaluation (*n* = 70 plasma concentration–time profiles and *n* = 64 renal excretion profiles).

**Figure 2:**
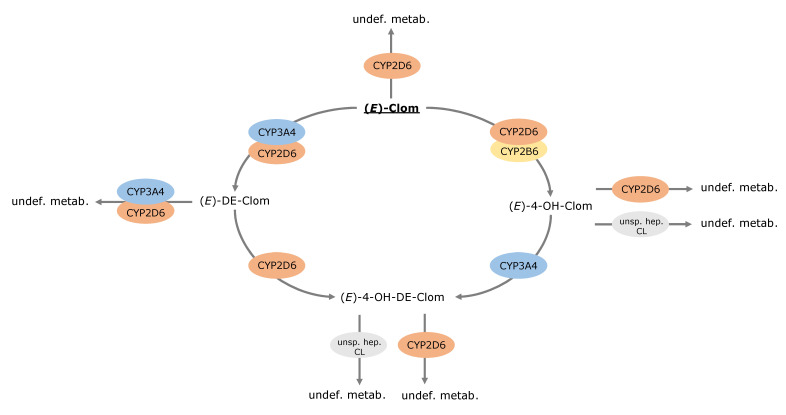
Overview of implemented metabolic processes in the (*E*)-Clom PBPK model. CYP, cytochrome P450; (*E*)-4-OH-Clom, (*E*)-4-hydroxyclomiphene; (*E*)-4-OH-DE-Clom, (*E*)-4-hydroxy-N-desethylclomiphene; (*E*)-Clom, (*E*)-clomiphene; (*E*)-DE-Clom, (*E*)-N-desethylclomiphene; undef. metab., undefined metabolite; unsp. hep. CL, unspecific hepatic clearance.

Metabolic pathways of (*E*)-Clom and its metabolites comprising hydroxylation, N-de-ethylation and glucuronidation, among others, were implemented via CYP enzymes (CYP2D6, CYP3A4 and CYP2B6) and unspecific hepatic clearance mechanisms (Figure 2). In summary, (*E*)-Clom is primarily metabolized via CYP2D6 to the active metabolite (*E*)-4-OH-Clom as well as to (*Z*)-3-hydroxyclomiphene (implemented as an undefined metabolite) [6]. An additional biotransformation process via CYP2B6 to (*E*)-4-OH-Clom was implemented to cover the fraction of CYP2D6-independent metabolism observed in the PM population and in CYP2D6 DD(G)I scenarios [5,6]. Biotransformation of (*E*)-Clom to (*E*)-DE-Clom was implemented mainly through CYP3A4 with CYP2D6 playing only a minor role in this metabolic pathway [5,28].

(*E*)-4-OH-Clom is metabolized via CYP2D6 to (*Z*)-3,4-dihydroxyclomiphene (implemented as an undefined metabolite), via an unspecific hepatic clearance mechanism and via CYP3A4 to the second active metabolite (*E*)-4-OH-DE-Clom [5,6,28]. (*E*)-4-OH-DE-Clom is also formed via CYP2D6 metabolism of (*E*)-DE-Clom, which in turn, represents the main route of elimination of (*E*)-DE-Clom [5,28]. Furthermore, (*E*)-DE-Clom is metabolized to minor extents through CYP2D6 and CYP3A4 to (*E*)-N,N-didesethylclomiphene (implemented as an undefined metabolite) [5,28]. The metabolism of (*E*)-4-OH-DE-Clom has not been extensively investigated, yet. According to work by Kröner [6], (*E*)-4-OH-DE-Clom is presumably metabolized through a CYP-mediated pathway to (*Z*)-3,4-dihydroxydesethyl-clomiphene. Additionally, glucuronidation, sulfation and potentially further unexplored pathways play a role in (*E*)-4-OH-DE-Clom biotransformation [6] and were grouped under an unspecific hepatic clearance process in the PBPK model (Figure 2).

Renal excretion through glomerular filtration was implemented and potential reabsorption or secretion processes were informed via renal excretion data. Model parameters that could not be informed from experimental reports during model development were optimized by fitting the model to the observed data of the training dataset. Moreover, a fraction of (*E*)-Clom metabolized via CYP3A4 was calculated (see Section S1.5 of the supplementary document) and used to inform k_cat_ model parameters associated with (*E*)-Clom metabolism. For detailed information on PBPK model building, see Section S1 of the supplementary document.

### 2.4. DGI and DD(G)I Modeling

Using the training dataset, k_cat_ values for CYP2D6-mediated pathways were estimated for the NM population, while CYP2D6 k_cat_ values for the PM population were set to zero. To predict DGIs and plasma concentration–time profiles in the IM and UM populations, IM and UM k_cat_ values for CYP2D6-dependent pathways were extrapolated from the estimated NM-k_cat_ value (Equation (1)):

(1) $$k_{\mathrm{cat},\;\mathrm{AS}=i}={\;k}_{\mathrm{cat},\;\mathrm{AS}=2}\;\cdot {\mathrm{IVSF}}_{i}$$

Here, k_cat, AS=i_ represents the catalytic rate constant for CYP2D6 AS = i, k_cat, AS=2_ is the catalytic rate constant for the NM population and IVSF_i_ is the corresponding in vitro scaling factor (IVSF). IVSFs were obtained using in vitro information on CYP2D6 AS-specific formation rates regarding the metabolism of (*E*)-Clom and its three metabolites (see Table S8 of the supplementary document) [5]. For predictions of plasma concentrations from clinical trials that did not report CYP2D6 phenotypes, CYP2D6 k_cat_ parameters were fitted to the respective plasma concentration–time profiles for each study.

In the DD(G)I setting, study participants in the pharmacokinetic panel study received clomiphene citrate together with the CYP3A4 inhibitor clarithromycin or the CYP2D6 inhibitor paroxetine that additionally acts as a weak inhibitor of CYP3A4 [19,29]. Predictions for DD(G)I scenarios of (*E*)-Clom and the investigated metabolites were performed for all CYP2D6 AS by coupling the developed parent–metabolite PBPK model with previously published PBPK models of the perpetrator drugs clarithromycin [16] and paroxetine [30]. Inhibition mechanisms of CYP3A4 and CYP2D6 were implemented as described in the OSP Suite manual [31]. Interaction parameters were used as published in the respective perpetrator PBPK models [16].

### 2.5. PBPK DGI and DD(G)I Model Evaluation

The performance of the parent–metabolite PBPK model was evaluated, applying several graphical and quantitative methods. The predicted plasma concentration–time profiles of (*E*)-Clom, (*E*)-4-OH-Clom, (*E*)-DE-Clom and (*E*)-4-OH-DE-Clom were graphically compared with their respective observed plasma profiles for all investigated CYP2D6 AS populations. Additionally, goodness-of-fit plots were used to compare predicted and observed areas under the plasma concentration–time curves from the first to the last time point of measurements (AUC_last_), C_max_ values and plasma concentrations of all model compounds for the DGI and DD(G)I scenarios. As quantitative measures, the mean relative deviation (MRD) of predicted plasma concentrations and the geometric mean fold error (GMFE) of predicted AUC_last_ and C_max_ were calculated according to Equations (2) and (3), respectively:

(2) $$\mathrm{MRD}=10^{x}\;\mathrm{with}\;x=\sqrt{\frac{1}{n}\;\sum_{i=1}^{n}{\left(\log_{10}\hat{c_{i}}-\log_{10}c_{i}\right)}^{2}}$$

Here, $\hat{c_{i}}$ represents the i-th predicted plasma concentration, c_i_ is the corresponding observed plasma concentration and n equals the number of observed values.

(3) $$\mathrm{GMFE}=10^{x}\;\mathrm{with}\;x=\frac{1}{n}\;\sum_{i=1}^{n}\left|\log_{10}\left(\frac{\hat{a_{i}}}{a_{i}}\right)\right|$$

Here, $\hat{a_{i}}$ represents the i-th predicted AUC_last_ and C_max_ value, respectively, a_i_ is the corresponding observed value and n equals the number of predicted plasma profiles.

For the evaluation of DGI and DD(G)I effects, the predicted AUC_last_ and C_max_ effect ratios were calculated according to Equations (4) and (5) and compared with the corresponding observed values. Here, model performance was assessed using the prediction acceptance limits proposed by Guest et al. with 1.25-fold variability [32].

(4) $${\mathrm{AUC}}_{\mathrm{last},\;\mathrm{AS}=i}\;\mathrm{ratio}=\frac{{\mathrm{AUC}}_{\mathrm{last},\;\mathrm{effect},\;\mathrm{AS}=i}}{{\mathrm{AUC}}_{\mathrm{last},\;\mathrm{control}}}$$

(5) $$C_{\max,\;\mathrm{AS}=i}\;\mathrm{ratio}=\frac{C_{\max,\;\mathrm{effect},\;\mathrm{AS}=i}}{C_{\max,\;\mathrm{control}}}$$

For the calculation of DGI ratios, AUC_last, effect, AS=i_ and C_max, effect, AS=i_ represent the AUC_last_ and C_max_ for CYP2D6 AS = i, while AUC_last, control_ and C_max, control_ are the AUC_last_ and C_max_ values for the NM (AS = 2) population. For the calculation of DD(G)I ratios, AUC_last, effect, AS=i_ and C_max, effect, AS=i_ represent the AUC_last_ and C_max_ for the CYP2D6 AS = i in the DD(G)I scenario with clarithromycin or paroxetine, while AUC_last, control_ and C_max, control_ are the AUC_last_ and C_max_ values for the CYP2D6 AS = i without the concomitant use of perpetrator drugs.

Moreover, a local sensitivity analysis was performed using PK-Sim^®^. A detailed description of the analysis and results is provided in Section S4.4 of the supplementary document.

## 3. Results

### 3.1. PBPK Model Building and Evaluation

The developed whole-body parent–metabolite PBPK model successfully described plasma concentration–time profiles and renal excretion profiles in NM and PM populations. In addition, DGI effects in IM and UM populations as well as DD(G)I scenarios with clarithromycin and paroxetine in various phenotypes could be successfully predicted. With that, the PBPK model of (*E*)-Clom and the three metabolites (*E*)-4-OH-Clom, (*E*)-DE-Clom and (*E*)-4-OH-DE-Clom was able to capture the complexity of the parent–metabolite network and was used to characterize the contribution of various elimination pathways.

For model building and evaluation, plasma concentration–time and renal excretion–time profiles of various CYP2D6 AS from a pharmacokinetic panel study as well as from four published clinical studies with a dose range from 6.25 mg to 62 mg of orally administered (*E*)-Clom citrate were included. In total, 22 plasma concentration–time profiles for (*E*)-Clom, 16 plasma profiles each for (*E*)-4-OH-Clom, (*E*)-DE-Clom and (*E*)-4-OH-DE-Clom as well as 64 renal excretion profiles were available. With the observed increase in exposure for NM during concomitant clarithromycin administration, a fraction metabolized (f_m_) of (*E*)-Clom via CYP3A4 of approximately 13% could be estimated (cf., Section S1.5 of the supplementary document) and subsequently integrated into the model building process to inform the contribution of the CYP3A4-dependent pathway. The drug-dependent model input parameters of (*E*)-Clom, (E)-4-OH-Clom, (*E*)-DE-Clom and (*E*)-4-OH-DE-Clom are provided in Tables S4–S7 of the supplementary document.

### 3.2. DGI Modeling and Evaluation

**Figure 3:**
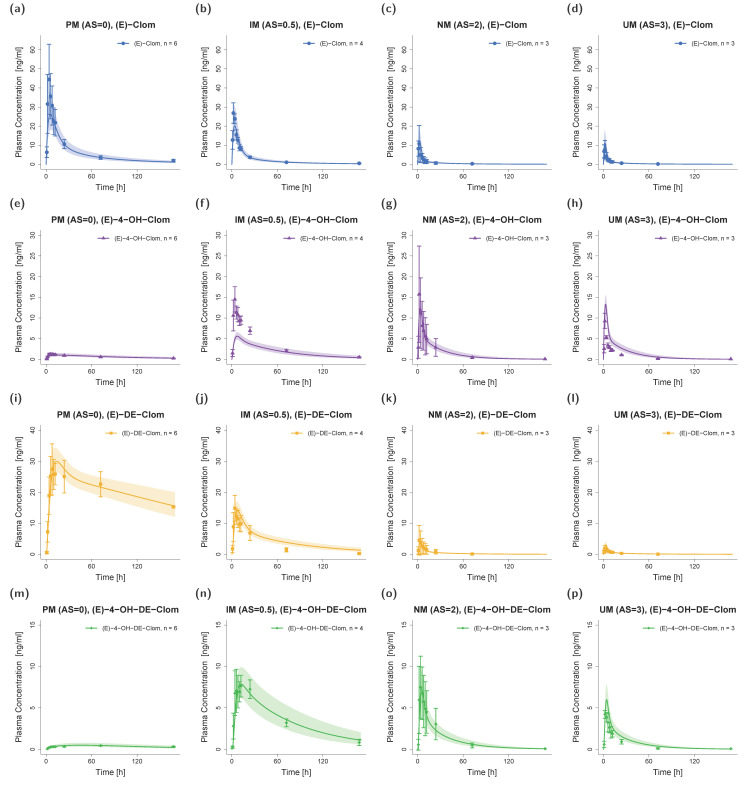
Predicted and observed plasma concentration–time profiles of (*E*)-Clom (**a**–**d**), (*E*)-4-OH-Clom (**e**–**h**), (*E*)-DE-Clom (**i**–**l**) and (*E*)-4-OH-DE-Clom (**m**–**p**) in PM (first column), IM (only AS = 0.5 shown; second column), NM (third column) and UM (last column) for DGI scenarios. Solid lines depict predicted geometric mean concentration–time profiles in the PM, IM (AS = 0.5), NM and UM populations. Colored ribbons show the corresponding geometric standard deviation of the population simulations (*n* = 1000). Mean observed data are shown as symbols with the corresponding standard deviation. Linear and semilogarithmic predicted and observed plasma concentration–time profiles of all studies and AS are shown in Section S4.1 of the supplementary document. AS, CYP2D6 activity score; DGI, drug–gene interaction; (*E*)-4-OH-Clom, (*E*)-4-hydroxyclomiphene; (*E*)-4-OH-DE-Clom, (*E*)-4-hydroxy-N-desethylclomiphene; (*E*)-Clom, (*E*)-clomiphene; (*E*)-DE-Clom, (*E*)-N-desethylclomiphene; IM, intermediate metabolizers; *n*, number of subjects; NM, normal metabolizers, PM, poor metabolizers; UM, ultrarapid metabolizers.

The final PBPK model precisely captured mean plasma concentration–time profiles of the NM (AS = 2) population for (*E*)-Clom and all three integrated metabolites (see Figure 3, third column). All predicted AUC_last_ and C_max_ values were in good agreement with the observed values: GMFEs for AUC_last_ and C_max_ in the NM population were 1.11 and 1.13, respectively. The overall MRD value for predicted plasma concentrations was 1.37.

For DGI model predictions, CYP2D6 k_cat_ values were extrapolated from NM to IM (AS = 0.5, AS = 0.75 and AS = 1) and UM populations. The extrapolation of k_cat_ parameters based on in vitro scaling factors led to successful predictions of plasma profiles in IM and UM phenotypes. Plasma profiles in PM volunteers that were part of the training dataset were also well captured in model simulations (Figure 3).

Since (*E*)-Clom is primarily metabolized via CYP2D6 (predicted f_m_ = 86%), the PM population showed the highest AUC_last_ for the parent compound (*E*)-Clom (AUC_PM_ > AUC_IM_ > AUC_NM_ > AUC_UM_), but the lowest AUC_last_ for the two most active metabolites (*E*)-4-OH-Clom and (*E*)-4-OH-DE-Clom. However, since (*E*)-4-OH-Clom and (*E*)-4-OH-DE-Clom were not only formed but also degraded via CYP2D6, their highest AUC_last_ could not be found in UM, but in IM with AS = 0.5 (AUC_IM (AS = 0.5)_ > AUC_NM_ > AUC_UM_ > AUC_PM_). A detailed listing of all predicted and observed AUC_last_ and C_max_ values for all phenotypes in the DGI study setting is depicted in Table S11 of the supplementary document.

**Figure 4:**
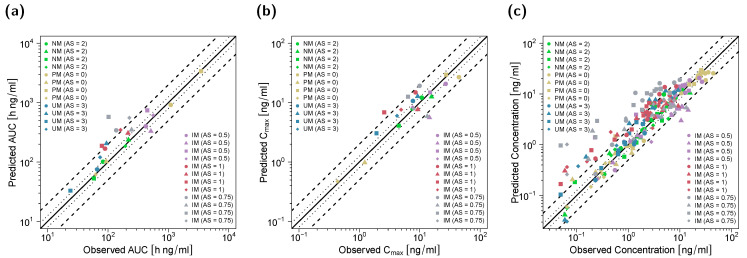
Predicted versus observed AUC_last_ (**a**), C_max_ (**b**) and plasma concentrations (**c**) of (*E*)-Clom (circles), (*E*)-4-OH-Clom (triangles), (*E*)-DE-Clom (squares) and (*E*)-4-OH-DE-Clom (diamonds) in PM, IM, NM and UM (DGI scenarios). The black solid lines mark the lines of identity. Black dotted lines indicate 1.25-fold; black dashed lines indicate two-fold deviation. Goodness-of-fit plots of digitized studies are depicted in Figure S8 of the supplementary document. AS, CYP2D6 activity score; DGI, drug–gene interaction; (*E*)-4-OH-Clom, (*E*)-4-hydroxyclomiphene; (*E*)-4-OH-DE-Clom, (*E*)-4-hydroxy-N-desethylclomiphene; (*E*)-Clom, (*E*)-clomiphene; (*E*)-DE-Clom, (*E*)-N-desethylclomiphene; IM, intermediate metabolizers; NM, normal metabolizers; PM, poor metabolizers; UM, ultrarapid metabolizers.

Goodness-of-fit plots for all modeled compounds showing predicted compared with observed plasma concentrations, AUC_last_ and C_max_ values in the DGI study setting are depicted in Figure 4. Here, 90% of C_max_, 80% of AUC_last_ and 78% of the predicted concentrations were within the two-fold acceptance criterion. GMFEs for the predicted C_max_ and AUC_last_ values were 1.41 and 1.43, respectively, and the overall MRD value for predicted plasma concentrations was 1.95.

**Figure 5:**
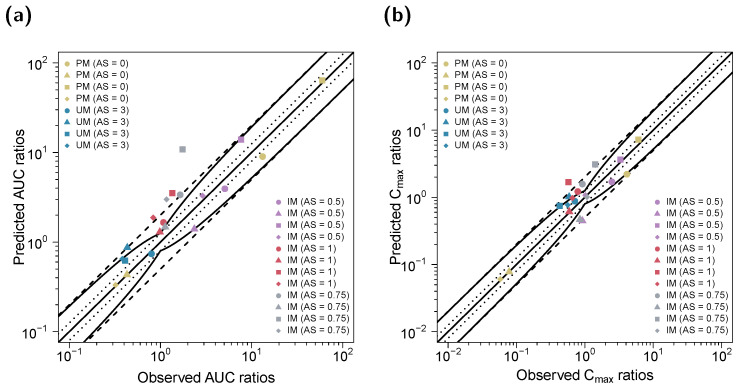
Predicted versus observed DGI (**a**) AUC_last_ and (**b**) C_max_ ratios of (*E*)-Clom (circles), (*E*)-4-OH-Clom (triangles), (*E*)-DE-Clom (squares) and (*E*)-4-OH-DE-Clom (diamonds). The straight black lines mark the lines of identity; the curved solid black lines show the limits of the predictive measure proposed by Guest et al. with 1.25-fold variability [32]. Black dotted lines indicate 1.25-fold; black dashed lines indicate two-fold deviation. AS, CYP2D6 activity score; (*E*)-4-OH-Clom, (*E*)-4-hydroxyclomiphene; (*E*)-4-OH-DE-Clom, (*E*)-4-hydroxy-N-desethylclomiphene; (*E*)-Clom, (*E*)-clomiphene; (*E*)-DE-Clom, (*E*)-N-desethylclomiphene; IM, intermediate metabolizers; NM, normal metabolizers; PM, poor metabolizers; UM, ultrarapid metabolizers.

The predicted impact of CYP2D6 polymorphisms on the PK of (*E*)-Clom and its three metabolites (DGI effect ratios) is shown in Figure 5 and is highly consistent with observed effects. GMFEs for the predicted C_max_ and AUC_last_ ratios in the DGI setting were 1.46 and 1.65, respectively. Predicted and observed renal excretion profiles are visualized in Section S4.1 of the supplementary document. Moreover, complementary prediction results of concentration–time profiles for the remaining AS and included published clinical studies are shown in Sections S4.1.3 and S4.1.7, respectively, of the supplementary document.

### 3.3. DD(G)I Modeling and Evaluation

**Figure 6:**
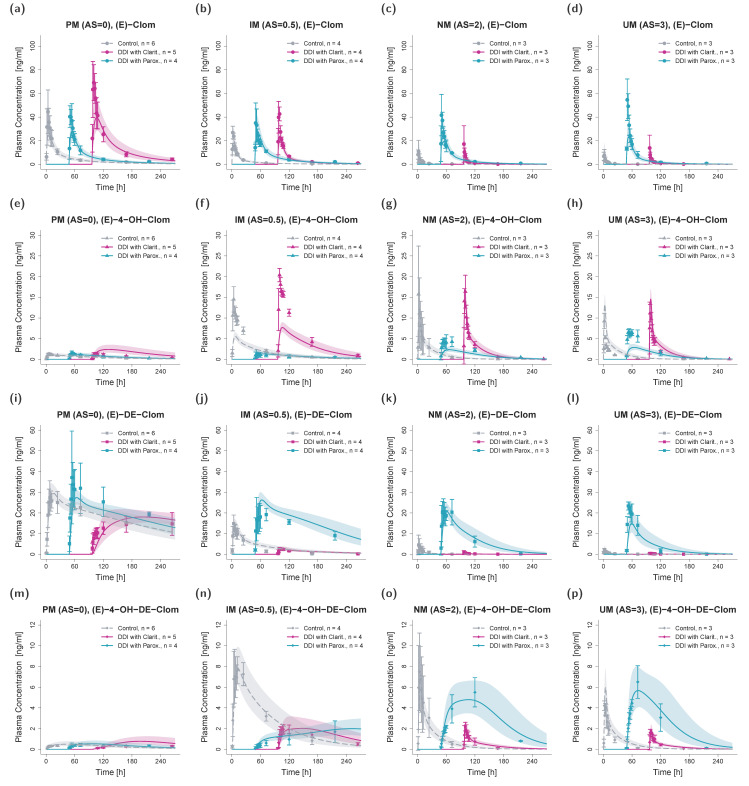
Predicted and observed plasma concentration–time profiles of (*E*)-Clom (**a**–**d**), (*E*)-4-OH-Clom (**e**–**h**), (*E*)-DE-Clom (**i**–**l**) and (*E*)-4-OH-DE-Clom (**m**–**p**) for DD(G)I scenarios in PM (first column), IM (only AS = 0.5 shown; second column), NM (third column) and UM (last column). Grey dashed lines depict the predicted geometric mean concentration–time profiles in absence of clarithromycin and paroxetine (control); turquoise solid lines represent the predicted geometric mean profiles in the presence of paroxetine; and pink solid lines represent the predicted geometric mean profiles in the presence of clarithromycin (DD(G)I). Colored ribbons show the corresponding geometric standard deviation of the population simulations (*n* = 1000). Mean observed data are shown as symbols with the corresponding standard deviation. Linear and semilogarithmic predicted and observed plasma concentration–time profiles of all AS are shown in Section S4.2 of the supplementary document. For better visibility, DD(G)I scenarios were plotted with a time offset with t = 0 at the first dose of the perpetrator drug. AS, CYP2D6 activity score; Clarit., Clarithromycin; DD(G)I, drug–drug and drug–drug–gene interactions; (*E*)-4-OH-Clom, (*E*)-4-hydroxyclomiphene; (*E*)-4-OH-DE-Clom, (*E*)-4-hydroxy-N-desethylclomiphene; (*E*)-Clom, (*E*)-clomiphene; (*E*)-DE-Clom, (*E*)-N-desethylclomiphene; IM, intermediate metabolizers; *n*, number of subjects; NM, normal metabolizers; Parox., Paroxetine; PM, poor metabolizers; UM, ultrarapid metabolizers.

**Figure 7:**
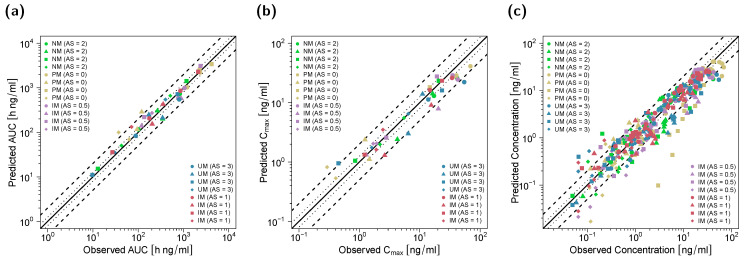
Predicted versus observed AUC_last_ (**a**), C_max_ (**b**) and plasma concentrations (**c**) of (*E*)-Clom (circles), (*E*)-4-OH-Clom (triangles), (*E*)-DE-Clom (squares) and (*E*)-4-OH-DE-Clom (diamonds) for DD(G)I scenarios with clarithromycin and paroxetine, respectively. The black solid lines mark the lines of identity. Black dotted lines indicate 1.25-fold; black dashed lines indicate two-fold deviation. AS, CYP2D6 activity score; DD(G)I, drug–drug and drug–drug–gene interactions; (*E*)-4-OH-Clom, (*E*)-4-hydroxyclomiphene; (*E*)-4-OH-DE-Clom, (*E*)-4-hydroxy-N-desethylclomiphene; (*E*)-Clom, (*E*)-clomiphene; (*E*)-DE-Clom, (*E*)-N-desethylclomiphene; IM, intermediate metabolizers; NM, normal metabolizers, PM, poor metabolizers; UM, ultrarapid metabolizers.

In total, 40 plasma concentration–time profiles and 40 renal excretion profiles of (*E*)-Clom and its metabolites were used for the investigation of DD(G)I scenarios with clarithromycin (mechanism-based inhibitor of CYP3A4) and paroxetine (mechanism-based inhibitor of CYP3A4 and CYP2D6) for various CYP2D6 AS (AS = 0, AS = 0.5, AS = 1, AS = 2 and AS = 3). Here, the impact of clarithromycin- and paroxetine-induced DD(G)I effects on plasma concentration–time profiles, AUC_last_ and C_max_ values of (*E*)-Clom and its metabolites was assessed. For this, published PBPK model parameters for clarithromycin [16] and paroxetine [30] were used including the respective competitive inhibition (K_i_) and the maximum inactivation rate (k_inact_) constants. Plasma and renal excretion profiles were predicted, compared with observed profiles and served for evaluations of DD(G)I model performance. DD(G)I model prediction performance is visually demonstrated in the concentration–time profiles (Figure 6) and the corresponding goodness-of-fit plots (Figure 7). GMFEs for the predicted AUC_last_ and C_max_ values were 1.30 and 1.40, respectively, and the overall MRD value for predicted plasma concentrations was 1.83.

Since the metabolism of (*E*)-Clom is predominantly mediated via CYP2D6, the AUC_last_ of (*E*)-Clom substantially increased with concomitant administration of the CYP2D6 inhibitor paroxetine (2.5–12-fold) for all phenotypes, except PM, which possess no CYP2D6 activity. Furthermore, due to inhibition of CYP2D6, C_max_ of the metabolite (*E*)-4-OH-Clom decreased in all phenotypes except for PM. However, as (*E*)-4-OH-Clom is not only formed but also degraded via CYP2D6, a substantial decrease in AUC_last_ during paroxetine DD(G)I was only predicted for the IM population in concordance with observed values. The minor involvement of CYP3A4 in the metabolism of (*E*)-Clom and (*E*)-4-OH-Clom is supported by the slight increase in the respective AUC_last_ during CYP3A4 inhibition in all phenotypes.

The AUC_last_ of (*E*)-DE-Clom is substantially reduced in all phenotypes by values between ~70% and 80% (NM and IM) and ~34% (PM) during concomitant clarithromycin administration, demonstrating that CYP3A4 is likely the major enzyme in the formation of (*E*)-DE-Clom. During CYP3A4 inhibition, AUC_last_ and C_max_ values, as well as the corresponding DDGI effects for (*E*)-4-OH-Clom and (*E*)-4-OH-DE-Clom in PM, were overpredicted by ~2.5-fold.

**Figure 8:**
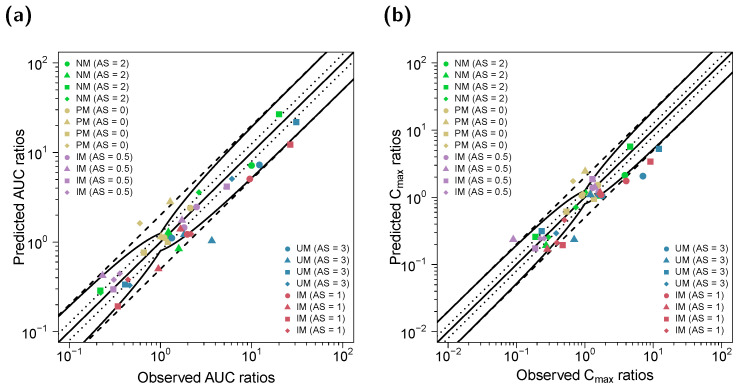
Predicted versus observed DD(G)I AUC_last_ (**a**) and C_max_ (**b**) ratios of (*E*)-Clom (circles), (*E*)-4-OH-Clom (triangles), (*E*)-DE-Clom (squares) and (*E*)-4-OH-DE-Clom (diamonds). The straight black lines mark the lines of identity; the curved black lines show the limits of the predictive measure proposed by Guest et al. with 1.25-fold variability [32]. Black dotted lines indicate 1.25-fold; black dashed lines indicate two-fold deviation. AS, CYP2D6 activity score; DD(G)I, drug–drug and drug–drug–gene interactions; (*E*)-4-OH-Clom, (*E*)-4-hydroxyclomiphene; (*E*)-4-OH-DE-Clom, (*E*)-4-hydroxy-N-desethylclomiphene; (*E*)-Clom, (*E*)-clomiphene; (*E*)-DE-Clom, (*E*)-N-desethylclomiphene; IM, intermediate metabolizers; NM, normal metabolizers, PM, poor metabolizers; UM, ultrarapid metabolizers.

Predicted and observed AUC_last_ and C_max_ ratios of (*E*)-Clom, (*E*)-4-OH-Clom, (*E*)-DE-Clom and (*E*)-4-OH-DE-Clom for the DD(G)I setting are shown in Figure 8. GMFEs for the predicted C_max_ and AUC_last_ ratios in the DD(G)I setting were 1.50 and 1.40, respectively. All predicted and observed values for AUC_last_ and C_max_, DD(G)I effect ratios as well as calculated MRDs and GMFEs are listed in Section S4.3 of the supplementary document.

### 3.4. Contribution of Metabolic Pathways to (E)-Clom and Metabolite Disposition

**Figure 9:**
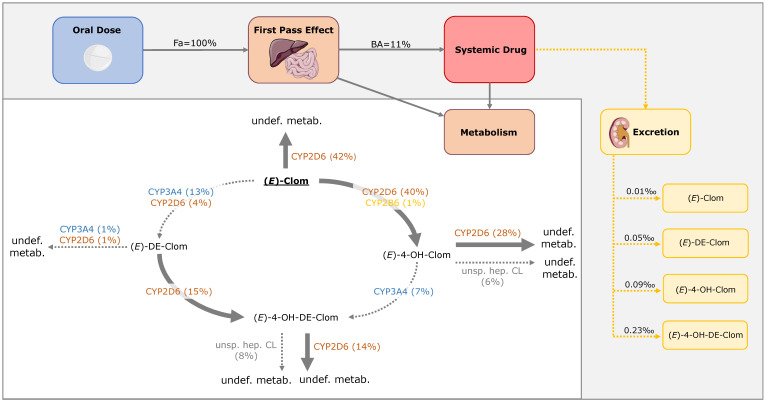
Mass balance diagram after oral administration of 62 mg (*E*)-Clom citrate in CYP2D6 normal metabolizers (AS = 2) including fraction absorbed, bioavailability and fractions of dose excreted in urine for (*E*)-Clom and the three implemented metabolites. Drawings by Servier, licensed under CC BY 3.0 [33]. BA, bioavailability; CL, clearance; CYP, cytochrome P450; (*E*)-4-OH-Clom, (*E*)-4-hydroxyclomiphene; (*E*)-4-OH-DE-Clom, (*E*)-4-hydroxy-N-desethylclomiphene; (*E*)-Clom, (*E*)-clomiphene; (*E*)-DE-Clom, (*E*)-N-desethylclomiphene; Fa, fraction absorbed; undef. metab., undefined metabolite; unsp. hep. CL, unspecific hepatic clearance.

In the PBPK model simulations, (*E*)-Clom is fully absorbed from the gastrointestinal tract (fraction absorbed = 1.0); however, it undergoes a substantial first-pass metabolism leading to a bioavailability of approximately 9% in UM, 11% in NM, 30% in IM (AS = 0.5) and 49% in PM. (*E*)-Clom is metabolized via three pathways to (*E*)-4-OH-Clom, (*E*)-DE-Clom and (Z)-3-hydroxyclomiphene with model-calculated f_m_ for NM of 41%, 17% and 42%, respectively (Figure 9).

The metabolism of the active metabolite (*E*)-4-OH-Clom in NM is mediated primarily via CYP2D6 (69%) and, to a minor extent, via an unspecific hepatic clearance (15%). Only 17% of (*E*)-4-OH-Clom is degraded to the second active metabolite (*E*)-4-OH-DE-Clom via CYP3A4. In addition, (*E*)-4-OH-DE-Clom is formed of (*E*)-DE-Clom via CYP2D6 (90% of (*E*)-DE-Clom elimination), while 10% of (*E*)-DE-Clom is metabolized via CYP2D6 and CYP3A4 to (*E*)-N,N-didesethylclomiphene. The renal excretion of (*E*)-Clom and its three metabolites can be considered negligible (0.01–0.23‰). Calculated contributions for all implemented metabolic pathways and fractions of dose excreted in urine of (*E*)-Clom and its metabolites in PBPK model simulations for NM as well as fractions of dose excreted in urine are illustrated in Figure 9.

## 4. Discussion

Since the approval of clomiphene for the treatment of anovulation in women by the U.S. Food and Drug Administration (FDA) in the late 1960s, several efforts have been made to explain the inter-individual variability in clomiphene PK and drug response [13,14,34,35,36]. While early studies identified obesity, hyperandrogenemia and high levels of serum anti-Müllerian hormone as predictors for non-response [34,35,37,38,39], polymorphisms of CYP2D6 were additionally identified to alter drug disposition and response [5,14,36]. This study presents the first (*E*)-Clom PBPK model that investigates and characterizes the impact of CYP2D6 polymorphisms and the concomitant use of CYP3A4 and CYP2D6 inhibitors on the PK of (*E*)-Clom and its three important metabolites (*E*)-4-OH-Clom, (*E*)-DE-Clom and (*E*)-4-OH-DE-Clom.

For this, a whole-body parent–metabolite PBPK model of (*E*)-Clom has been successfully built and evaluated, predicting plasma concentration–time profiles for various CYP2D6 AS in DGI and DD(G)I scenarios. The predicted DGI and DD(G)I effects on the PK of (*E*)-Clom and its active metabolites were in good agreement with the effects observed in a pharmacokinetic panel study. Despite the complex nature of the disposition of (*E*)-Clom and its metabolites, the PBPK model could capture and quantify the contribution of the different metabolic pathways. The developed model described and predicted plasma profiles of the training and test dataset for the DGI setting with GMFEs of 1.43 and 1.41 for predictions of AUC_last_ and C_max_, respectively. GMFEs in the DD(G)I settings with clarithromycin and paroxetine were 1.30 and 1.40 for predictions of AUC_last_ and C_max_, respectively, highlighting the good descriptive and predictive model performance.

DGI predictions for IM and UM populations were based on in vitro–in vivo extrapolation of CYP2D6 activity. Here, the application of AS-specific k_cat_ values based on estimated in vivo NM-k_cat_ and published in vitro information on differences in metabolic activity between CYP2D6 AS led to successful predictions of observed plasma concentrations and DGI effect ratios. The predicted DGI effects of CYP2D6 polymorphisms on the AUC of the four modeled compounds ranged from a ~60-fold increase ((*E*)-DE-Clom in PM vs. NM) to a ~70% decrease ((*E*)-4-OH-DE-Clom in PM vs. NM).

The observed DGI AUC_last_ effect ratio for (*E*)-Clom in IM (AS = 1) was ~1 representing “no effect”, while the model predicted effect ratio was about 1.7, suggesting a ~70% increase in AUC from NM to IM (AS = 1), which seems reasonable due to the strong CYP2D6 involvement in (*E*)-Clom degradation. The corresponding predicted effect on (*E*)-4-OH-DE-Clom exposure (~1.9) was also higher than the effect observed (~0.8). Similarly, DGI AUC_last_ effect ratios for IM (AS = 0.75) were higher than the corresponding effect ratios observed for (*E*)-Clom and its metabolites. Several genetic and non-genetic factors in addition to the *CYP2D6* genotype have previously been described to affect CYP2D6 activity in vivo, resulting in substantial interindividual variability in the PK of CYP2D6 substrates [5,40,41]. Here, the pharmacokinetic panel study might lack the required power to reliably predict the low observed mean effect ratios for IM (AS =1 and AS = 0.75) individuals (*n* = 2 and *n* =1, respectively). Thus, additional studies with an increased number of CYP2D6 genotyped individuals would be helpful to further evaluate these prediction scenarios.

The underprediction of (*E*)-4-OH-Clom AUC_last_ DGI effects in IM (AS = 0.5) and UM populations based on the in vitro–in vivo extrapolation of CYP2D6 activity could hint towards a stronger involvement of CYP2D6 in the metabolism of (*E*)-4-OH-Clom or indicate lower CYP2D6 k_cat_ values in IM and higher values in UM than was extrapolated from in vitro. Moreover, the relative importance of other enzymes for pathways mediated by CYP2D6 increases for lower CYP2D6 AS. Consequently, the impact of variability in activity for alternative pathways (e.g., due to polymorphisms in *CYP2B6*) increases [41,42]. Notably, only a small number of participants (*n* = 3) in the pharmacokinetic panel study were assigned to the IM (AS = 0.5) group and were genotyped for *CYP2D6* only. Hence, as a result of the underprediction (IM (AS = 0.5)) and overprediction (UM) of (*E*)-4-OH-Clom exposure, respectively, DD(G)I model predictions for this metabolite should be interpreted carefully in these populations.

Since (*E*)-Clom is primarily metabolized via CYP2D6 (f_m_ of ~86% according to model simulations) PM showed the highest exposure for the parent compound (AUC_last, (*E*)-Clom_ order: PM > IM > NM > UM). Additionally, as (*E*)-4-OH-DE-Clom is primarily formed via CYP2D6-dependent pathways, PM showed the lowest AUC_last_ for the active metabolite. However, the complex metabolic network with additional involvement of other CYP enzymes and contribution of multiple CYP2D6-dependent pathways resulted in a different order for (*E*)-4-OH-DE-Clom AUC values compared with (*E*)-Clom. Here, the AUC_last_ of (*E*)-4-OH-DE-Clom was highest in IM (AS = 0.5), while it was lowest for PM and second-lowest for UM, proposing a contribution of CYP2D6 not only in the formation but also in the degradation of (*E*)-4-OH-DE-Clom. This is supported by model simulations, where the integration of a CYP2D6 metabolic route for (*E*)-4-OH-Clom and (*E*)-4-OH-DE-Clom degradation [6,28] was crucial for successful predictions of the respective plasma profiles. The involvement of CYP2D6 in the degradation of the active metabolites might also explain findings from a study by Ji et al., where all nine study participants with IM phenotype responded to clomiphene therapy, whereas 30% of NM were non-responders [14].

For the investigated clarithromycin DD(G)I scenario, (*E*)-Clom exposure increased by only ~15% for NM compared with the control scenario without CYP3A4 inhibition. In contrast, for PM, (*E*)-Clom exposure increased ~2.4-fold, which was successfully predicted by the PBPK model. The increase in (*E*)-Clom AUC_last_, however, also led to a model-predicted increase in (*E*)-4-OH-Clom AUC_last_ (~2.8-fold) and consequently to an increase in (*E*)-4-OH-DE-Clom AUC_last_ (~1.6-fold) for PM. This elevation was not observed in the available clinical data (effect ratio ~1.3-fold and ~0.6-fold, respectively). These differences between observation and prediction might be attributed to a saturated CYP2B6 metabolism from (*E*)-Clom to (*E*)-4-OH-Clom in vivo that was not reflected in the PBPK model or to non-implemented alternative metabolic pathways that are active in scenarios of low CYP3A4 and CYP2D6 activity.

The underprediction of paroxetine DDGI effects on (*E*)-4-OH-Clom AUC_last_ in the IM (AS = 0.5) and UM population supports the aforementioned hint towards lower CYP2D6 k_cat_ values in IM and higher values in UM or a stronger involvement of CYP2D6 in the metabolism of (*E*)-4-OH-Clom than was extrapolated from in vitro.

Many different CYP enzymes are involved in the metabolic pathways of (*E*)-Clom and its metabolites [5,28]; therefore, the implementation of biotransformation generally focused on main CYP enzymes. However, of note, the implementation of CYP2D6 as an additional enzyme, complementing CYP3A4 in the formation of (*E*)-DE-Clom [43], led to a substantial improvement in the prediction of clarithromycin DD(G)I scenarios, preventing an underprediction of AUC_last_ values for (*E*)-DE-Clom. Here, CYP2D6 was incorporated with a ~20% contribution to the formation of the desethyl metabolite [43].

In contrast, the initial assumption of a CYP3A4-mediated desethylation of (*E*)-4-OH-DE-Clom (as for (*E*)-4-OH-Clom, cf. Figure 9) was rejected, since this implemented process led to a consistent overprediction of (*E*)-4-OH-DE-Clom AUC_last_ in the clarithromycin DD(G)I scenarios for all phenotypes. Instead, the metabolic pathway was replaced by an unspecific hepatic clearance process representing glucuronidation, sulfation and potential other metabolic processes of (*E*)-4-OH-DE-Clom as suggested by Kröner [6].

PBPK modeling was also leveraged to gain insights into the PK of (*E*)-Clom and to investigate contributions of the different metabolic pathways for (*E*)-Clom and its metabolites. According to model simulations in NM, about 22% of the administered (*E*)-Clom dose is eventually metabolized to the metabolite with the highest target affinity ((*E*)-4-OH-DE-Clom [28]), mainly via the (*E*)-DE-Clom-pathway (~69%) and ~31% via the (*E*)-4-OH-Clom pathway. This is of note, as only ~17% of (*E*)-Clom is initially metabolized to (*E*)-DE-Clom, while ~41% is metabolized to (*E*)-4-OH-Clom. However, ~90% of (*E*)-DE-Clom metabolism results in (*E*)-4-OH-DE-Clom formation (vs. only ~17% of (*E*)-4-OH-Clom metabolism), eventually representing the main pathway of (*E*)-4-OH-DE-Clom formation according to model simulations.

Clomiphene is typically administered as a racemic mixture of (*E*)- and (*Z*)-Clom (62:38) [22]. Both isomers show highly distinct pharmacokinetic characteristics and also differ in affinity to the target receptor [22,28]. In contrast to (*Z*)-Clom, (*E*)-Clom undergoes an extensive first-pass metabolism resulting in a lower bioavailability [44]. The model predicted bioavailability for (*E*)-Clom in NM was ~11%, which is in congruence with the low bioavailability of ~6.3% for the (*E*)-isomer calculated from the reported AUC_0-24h_ after oral [21] and intravenous application of 50 mg clomiphene citrate [45]. While the calculated value from the literature is based on an intravenous study with a small number of study participants (*n* = 2) [45], a low bioavailability can be supported with the developed PBPK model. The model calculated bioavailabilities in PM, IM (AS = 0.5, AS = 0.75, AS = 1) and UM were 49%, 30%, 27%, 18% and 9%, respectively.

In the pharmacokinetic panel study, renal excretion of the parent compound (*E*)-Clom and the three modeled metabolites was quantified and showed negligible overall contribution to the respective compound elimination. The PBPK model was able to quantify this small contribution of renal excretion for the four investigated compounds. The respective simulated fractions of dose excreted in urine for NM were calculated to be 0.01‰, 0.09‰, 0.05‰ and 0.23‰, for (*E*)-Clom, (*E*)-4-OH-Clom, (*E*)-DE-Clom and (*E*)-4-OH-DE-Clom, respectively. This is in concordance with recent studies, where unchanged (*E*)-Clom and unconjugated metabolites could only be detected in small amounts, or not at all in urine samples [46,47].

The pharmacokinetic panel study was conducted in a cross-over design [28]. One limitation of this work is the small number of participants in the panel study (*n* = 20), with only one to six individuals per AS group available for model development. Additionally, from the PM group, one participant dropped out of the clinical trial during the clarithromycin DDGI scenario and two participants during the paroxetine DDGI scenario. In the case of the IM (AS = 0.75) group, no data for the DDGI scenarios were available due to drop-out.

When additional pharmacokinetic data become available, the PBPK model can be further evaluated according to the “learn–confirm–refine” principle [48,49] to be used for further model applications. Moreover, the presented parent–metabolite PBPK model of (*E*)-Clom provides a basis for future investigations of different covariates (e.g., body mass index), individual CYP2D6 genotypes and the concomitant use of additional perpetrator drugs influencing the PK of (*E*)-Clom and its metabolites. The evaluated model can be leveraged to simulate plasma concentration–time profiles and investigate the exposure of (*E*)-Clom and its active metabolites in as-yet unexplored DD(G)I scenarios with the concomitant administration of moderate and weak CYP enzyme inhibitors as well as CYP enzyme inducers (e.g., carbamazepine [15]). Here, future clinical investigations of DD(G)I scenarios with concomitant use of (*E*)-Clom and CYP enzyme inducers are required for evaluation of such model predictions with clinically observed data. For the translation of exposure differences into dose recommendations, studies quantifying the efficacy- and safety-related contributions of (*E*)-Clom and its metabolites would be of high interest.

## 5. Conclusions

A whole-body parent–metabolite PBPK model of (*E*)-Clom including the metabolites (*E*)-4-OH-Clom, (*E*)-DE-Clom and (*E*)-4-OH-DE-Clom was successfully developed. The model predicted plasma concentration–time profiles of (*E*)-Clom and its metabolites for CYP2D6 DGI, as well as CYP2D6 and CYP3A4 DDI and DDGI scenarios in six different CYP2D6 AS groups. For this, an in vitro–in vivo extrapolation approach to obtain CYP2D6 k_cat_ values for different AS was successfully integrated to predict plasma profiles for IM (AS = 0.5, AS = 0.75, AS = 1) and UM populations. Furthermore, the model was applied to investigate the contribution of metabolic pathways to the elimination of (*E*)-Clom and its metabolites. The developed PBPK model will be made publicly available (http://models.clinicalpharmacy.me/) and can be further leveraged to investigate the PK of (*E*)-Clom and its metabolites for various DD(G)I scenarios.

## Figures and Tables

**Table 1:** Overview of clinical data integrated from the pharmacokinetic panel study.

	AS = 0	AS = 0.5	AS = 0.75	AS = 1	AS = 2	AS = 3
*n*	6 ^#^	4	1 ^+^	2	3	3
CYP2D6 phenotypes	PM	IM	IM	IM	NM	UM
*CYP2D6* genotypes	*4/*4 *4/*5 *4/*6	*4/*41 *4/*9	*9/*10	*1/*4	*1/*1	*1/*1 × 3
Demographics						
Age [years]	25.2 (22–29)	24.3 (21–30)	22.0 (-)	25.5 (23–28)	32.3 (26–43)	25.7 (22–28)
Weight [kg]	62.3 (50.0–70.0)	59.3 (55.5–64.0)	63.0 (-)	68.8 (63.5–74.0)	56.5 (48.0–63.5)	61.7 (54.0–73.0)
Height [cm]	1.70 (1.53–1.75)	1.68 (1.59–1.72)	1.66 (-)	1.71 (1.68–1.73)	1.63 (1.60–1.67)	1.65 (1.57–1.75)
BMI [kg/m^2^]	21.6 (20.6–22.9)	21.1 (20.3–22.0)	22.9 (-)	23.6 (22.5–24.7)	21.3 (18.8–24.2)	22.6 (20.3–23.8)

^#^ number of study participants decreased during the DDGI setting due to drop-outs (*n* = 5 for clarithromycin, *n* = 4 for paroxetine); ^+^ one study participant classified as AS = 0.75 was excluded from the analysis (see Section S1.1 of the supplementary document); demographic parameters are presented as mean (range); AS, CYP2D6 activity score; BMI, body mass index; IM, intermediate metabolizers; *n*, number of subjects; NM, normal metabolizers; PM, poor metabolizers; UM, ultrarapid metabolizers.

## Data Availability

The developed PBPK model will be made publicly available (http://models.clinicalpharmacy.me/).

## Supplementary Materials

The following supporting information can be downloaded at: https://www.mdpi.com/article/10.3390/pharmaceutics14122604/s1, Figure S1: Predicted and observed plasma concentration-time profiles (linear scale) of (*E*)-Clom (a–f), (*E*)-4-OH-Clom (g–l), (*E*)-DE-Clom (m–r) and (*E*)-4-OH-DE-Clom (s–x) for DGI scenarios; Figure S2: Predicted and observed plasma concentration-time profiles (semilogarithmic scale) of (*E*)-Clom (a–f), (*E*)-4-OH-Clom (g–l), (*E*)-DE-Clom (m–r) and (*E*)-4-OH-DE-Clom (s–x) for DGI scenarios; Figure S3: Predicted versus observed AUClast (a), Cmax (b) and plasma concentrations (c) of (E)-Clom (circles), (*E*)-4-OH-Clom (triangles), (*E*)-DE-Clom (squares) and (*E*)-4-OH-DE-Clom (diamonds) in PM, IM, NM and UM (DGI scenarios); Figure S4: Predicted versus observed DGI AUClast (a) and Cmax (b) ratios of (*E*)-Clom (circles), (*E*)-4-OH-Clom (tri-angles), (*E*)-DE-Clom (squares) and (*E*)-4-OH-DE-Clom (diamonds) in PM, IM and UM; Figure S5: Predicted and observed renal excretion profiles (linear scale) of (*E*)-Clom (a–f), (*E*)-4-OH-Clom (g–l), (*E*)-DE-Clom (m–r) and (*E*)-4-OH-DE-Clom (s–x) for DGI scenarios; Figure S6: Predicted and observed plasma concentration-time profiles (linear scale) of digitized studies from literature after single (a,b) and multiple (c–f) dosing; Figure S7: Predicted and observed plasma concentration-time profiles (semilogarithmic scale) of digitized studies from literature after single (a,b) and multiple (c–f) dosing; Figure S8: Predicted versus observed (a) AUClast, (b) Cmax and (c) plasma concentrations of (*E*)-Clom; Figure S9: Predicted and observed plasma concentration-time profiles (linear scale) of (E)-Clom (a–e), (*E*)-4-OH-Clom (f–j), (*E*)-DE-Clom (k–o) and (*E*)-4-OH-DE-Clom (p–t) for DD(G)I scenarios in PM, IM, NM and UM; Figure S10: Predicted and observed plasma concentration-time profiles (semilogarithmic scale) of (*E*)-Clom (a–e), (*E*)-4-OH-Clom (f–j), (*E*)-DE-Clom (k–o) and (*E*)-4-OH-DE-Clom (p–t) for DD(G)I scenar-ios in PM, IM, NM and UM; Figure S11: Predicted versus observed AUClast (a), Cmax (b) and plasma concentrations (c) of (*E*)-Clom (circles), (*E*)-4-OH-Clom (triangles), (*E*)-DE-Clom (squares) and (*E*)-4-OH-DE-Clom (diamonds) for DD(G)I scenarios with clarithromycin and paroxetine, respectively in PM, IM, NM and UM; Figure S12: Predicted versus observed DD(G)I AUClast (a) and Cmax (b) ratios of (*E*)-Clom (circles), (*E*)-4-OH-Clom (triangles), (*E*)-DE-Clom (squares) and (*E*)-4-OH-DE-Clom (diamonds) in PM, IM, NM and UM; Figure S13: Predicted and observed renal excretion profiles (linear scale) of (*E*)-Clom (a–e), (*E*)-4-OH-Clom (f–j), (*E*)-DE-Clom (k–o) and (*E*)-4-OH-DE-Clom (p–t) for DD(G)I scenarios in PM, IM, NM and UM; Figure S14: Sensitivity analysis of the PBPK model for (*E*)-Clom, (*E*)-4-OH-Clom, (*E*)-DE-Clom and (*E*)-4-OH-DE-Clom; Figure S15: Molecular structures of (*E*)-Clom (a) and its metabolites (*E*)-DE-Clom (b), (*E*)-4-OH-Clom (c) and (*E*)-4-OH-DE-Clom (d); Table S1: Optimized CYP2D6 kcat values for each study; Table S2: Overview of clinical study data from literature used for model evaluation; Table S3: System-dependent parameters and expression of relevant enzymes; Table S4: Drug-dependent parameters for (*E*)-clomiphene; Table S5: Drug-dependent parameters for (*E*)-N-desethylclomiphene; Table S6: Drug-dependent parameters for (*E*)-4-hydroxyclomiphene; Table S7: Drug-dependent parameters for (*E*)-4-hydroxy-N-desethyl-clomiphene; Table S8: Employed in vitro scaling factors (IVSFs) for individual CYP2D6 activity scores; Table S9: Mean relative deviation (MRD) values of DGI plasma concentration predictions; Table S10: Mean relative deviation (MRD) values of DD(G)I plasma concentration predictions; Table S11: Geometric Mean Fold Error (GMFE) of AUClast and Cmax DGI Predictions; Table S12: Geometric Mean Fold Error (GMFE) of DGI AUC_last_ and C_max_ ratio; Table S13: Geometric Mean Fold Error (GMFE) of AUC_last_ and C_max_ DD(G)I Predictions; Table S14: Geometric Mean Fold Error (GMFE) of DD(G)I AUC_last_ and C_max_ ratios. References [50,51,52,53,54,55,56,57,58,59,60,61,62,63,64,65,66,67,68,69,70,71,72,73,74,75,76,77,78,79,80] are cited in the Supplementary Materials.
