# Supplementary material for: Prediction of Drug–Drug–Gene Interaction Scenarios of (E)-Clomiphene and Its Metabolites Using Physiologically Based Pharmacokinetic Modeling
Source: Pharmaceutics. 2022 Nov 25;14(12):2604. doi: 10.3390/pharmaceutics14122604 (PMC9781104; doi:10.3390/pharmaceutics14122604)
Supplement: Supplementary file 1 [file pharmaceutics-14-02604-s001.zip › pharmaceutics-2020207-supplementary.pdf]

Pharmaceutics

Supplementary Materials: Prediction of Drug-Drug-Gene Interaction Scenarios of (*E*)-Clomiphene and its Metabolites Using Physiologically Based Pharmacokinetic Modeling

Christina Kovar [1,2], Lukas Kovar [1], Simeon Rüdesheim [1,2], Dominik Selzer [1], Boian Ganchev [2], Patrick Kröner [2], Svitlana Igel [2], Reinhold Kerb [2], Elke Schaeffeler [2], Thomas E. Mürdter [2], Matthias Schwab [2,3] and Thorsten Lehr [1,*]

[1] Clinical Pharmacy, Saarland University, 66123 Saarbrücken, Germany

[2] Dr. Margarete Fischer-Bosch Institute of Clinical Pharmacology, University of Tübingen, 70376 Stuttgart, Germany

[3] Departments of Clinical Pharmacology, Pharmacy and Biochemistry, University of Tübingen, 72076 Tübingen, Germany

* Correspondence: thorsten.lehr@mx.uni-saarland.de; Tel.: +49-681-302-70255

Contents

S1 PBPK Model Building **3**

S1.1 Clinical Studies . . . 3
S1.2 System-dependent Parameters and Virtual Populations . . . 5
S1.3 Supplementary Information on (E)-clomiphene PBPK Model Building . . . 7
S1.4 Drug-dependent Parameter Tables . . . 8
S1.5 Calculation of Fractions Metabolized . . . 11
S1.6 Formulations . . . 12
S1.7 Handling Data Below the Lower Limit of Quantification (LLOQ) . . . 12

S2 Drug-Gene-Interaction (DGI) Modeling **13**

S2.1 CYP2D6 *in vitro* Scaling Factors . . . 13

S3 Drug-Drug-(Gene)-Interaction (DD(G)I) Modeling **13**

S3.1 Clarithromycin and Paroxetine . . . 13

S4 PBPK Model Evaluation **14**

S4.1 Evaluation of the DGI Model . . . 14
S4.1.1 Plasma Profiles (Linear Scale) . . . 14
S4.1.2 Plasma Profiles (Semilogarithmic Scale) . . . 17
S4.1.3 Goodness-of-Fit Plots . . . 20
S4.1.4 Renal Excretion Profiles (Linear Scale) . . . 22
S4.1.5 Plasma Profiles from Literature (Linear Scale) . . . 25
S4.1.6 Plasma Profiles from Literature (Semilogarithmic Scale) . . . 26
S4.1.7 Goodness-of-Fit Plots (from Literature) . . . 27
S4.2 Evaluation of the DD(G)I Model . . . 28
S4.2.1 Plasma Profiles (Linear Scale) . . . 28
S4.2.2 Plasma Profiles (Semilogarithmic Scale) . . . 31
S4.2.3 Goodness-of-Fit Plots . . . 34
S4.2.4 Renal Excretion Profiles (Linear Scale) . . . 36
S4.3 Quantitative PBPK Model Evaluation . . . 39
S4.3.1 Mean Relative Deviation (MRD) . . . 39
S4.3.2 Geometric Mean Fold Error (GMFE) . . . 41
S4.4 Local Sensitivity Analysis . . . 48
S4.4.1 Mathematical Implementation . . . 48
S4.4.2 Results of the Sensitivity Analysis . . . 49

S5 Molecular **50**

References **51**

S1. PBPK Model Building

S1.1. Clinical Studies

Plasma and renal excretion profiles of (E)-clomiphene ((E)-Clom) and its metabolites from a pharmacokinetic panel study with 20 healthy female volunteers, that were assigned to six different cytochrome P450 (CYP) 2D6 activity scores (AS), were available for model building and evaluation (see Table 1 in the main manuscript for demographic information). The pharmacokinetic panel study was complemented with digitized data from published clinical studies (study search criteria were (a) studies with intravenous or oral (E)-Clom administration and (b) reported pharmacokinetic data of (E)-Clom and/or its metabolites (E)-4-hydroxyclomiphene ((E)-4-OH-Clom), (E)-N-desethylclomiphene ((E)-DE-Clom) and (E)-4-hydroxy-N-desethylclomiphene ((E)-4-OH-DE-Clom). Data originating from two single dose and two multiple dose studies with oral (E)-Clom administration could be integrated. To the best of our knowledge, plasma profiles of (E)-Clom and its metabolites after intravenous administration were not publicly available. Information on the identified and integrated published clinical studies are listed in Table S2. As CYP2D6 AS and phenotype of corresponding study participants were not reported, CYP2D6 catalytic rate constants (k_{cat}) values in the PBPK model were estimated (see Table S1).

Table S1. Optimized CYP2D6 k_{cat} values for each study.

**CYP2D6 k_{cat} values**	**Mikkelson et al. 1986 [1]**	**Study Ratioph. 1991 [2]**	**Wiehle et al. 2013 (a) [3]**	**Wiehle et al. 2013 (b)[3]**	**Wiehle et al. 2013 (c) [3]**	**Miller et al. 2018 [4]**
(E)-Clom → (E)-4-OH-Clom	213.0	283.1	87.7	124.1	43.3	18.1
(E)-Clom → undef.	90.6	120.5	37.3	52.8	18.4	7.7
(E)-Clom → (E)-DE-Clom	84.4	112.1	34.8	49.1	17.1	7.2

CYP: cytochrome P450, **(E)-4-OH-Clom**: (E)-4-hydroxyclomiphene, **(E)-Clom**: (E)-clomiphene, **(E)-DE-Clom**: (E)-N-desethylclomiphene, **k_{cat}**: catalytic rate constant, **Ratioph.**: Ratiopharm® GmbH, **undef.**: undefined metabolite

Of note, in the pharmacokinetic panel study, two study participants with the *CYP2D6* genotypes $^*9/^*10$ and $^*9/^*41$ had been classified as AS=0.75. Here, a high interindividual variability in the plasma profiles could be observed. The study participant genotyped as $^*9/^*41$ showed unexpectedly high (E)-Clom plasma concentrations for an AS=0.75 individual with (E)-Clom levels comparable with those of poor metabolizers (PM). Since the allele haplotype ^{*41} has shown a high dispersion in CYP2D6 enzyme activity, the respective individual was excluded from the dataset [5].

Table S2. Overview of clinical study data from literature used for model evaluation.

**Clinical study**	**Route**	**Dose [mg]**[a]	***E/Z* ratio**	**n**	**Females [%]**	**Age [years]**[b]	**Weight [kg]**[b]	**BMI [kg/m²]**[b]	**Metabolites measured**	***CYP2D6* genotyped**
Mikkelson et al. 1986 [1]	po, tab, s.d.	50	-[c]	23	100	32	62.4	-	no	no
Study Ratioph. 1991 [2]	po, tab, s.d.	50	62/38	18	-	-	-	-	no	no
Wiehle et al. 2013 (a) [3]	po, caps, m.d.	6.25	100/0	16	0	53.3±10.2	-	34.7±7.2	no	no
Wiehle et al. 2013 (b) [3]	po, caps, m.d.	12.5	100/0	14	0	53.3±10.2	-	34.7±7.2	no	no
Wiehle et al. 2013 (c) [3]	po, caps, m.d.	25	100/0	16	0	53.3±10.2	-	34.7±7.2	no	no
Miller et al. 2018 [4]	po, tab, m.d.	50	62/38	12	0	31.5±3.6	77.9±8.2	24.4±2.4	no	no

BMI: body mass index, **caps**: capsule, **CYP**: cytochrome P450, ***E/Z***: (*E*)-/(*Z*)-clomiphene, **m.d.**: multiple dose, **n**: number of subjects, **po**: per oral, **Ratioph.**: Ratiopharm® GmbH, **s.d.**: single dose, **tab**: tablet

[a] (E)-/(Z)-clomiphene citrate

[b] mean (±SD) if applicable

[c] E/Z-ratio of 62/38 was assumed

S1.2. System-dependent Parameters and Virtual Populations

Virtual individuals were created in PK-Sim®, using the published information on the respective study population, including mode of ethnicity and gender as well as mean values of age, weight and height. For the study population in the study from Ratiopharm® GmbH [2], demographic information were not provided. Here, the default values of a 30-year-old male European individual with body weight of 73 kg and height of 176 cm according to the International Commission on Radiological Protection (ICRP) reference values were used [6]. Distribution and abundance of enzymes in the different tissues was implemented according to the PK-Sim® expression database [7]. For the generation of virtual populations, 1000 individuals were created according to the respective study population demographics. Demographic characteristics of virtual individuals were varied within the ICRP [6] and the third National Health and Nutrition Examination Survey (NHANES) [8] limits by an implemented algorithm in PK-Sim®. The corresponding algorithms for the generation of virtual populations have been reported by Willmann and coworkers [9]. For the study by Mikkelson et al. [1] and the study from Ratiopharm® GmbH [2] an age range of 20 to 50 years was assumed.

Variabilities for CYP2B6 and CYP3A4 enzyme abundances in the virtual populations were integrated and variability in CYP2D6 abundance was allowed for study populations that were not genotyped and thus not stratified by CYP2D6 AS. For the pharmacokinetic panel study, CYP2D6 k_{cat} values differ across CYP2D6 AS groups, already accounting for varying CYP2D6 abundance and/or activity. Thus, CYP2D6 expression variability was set to 0 for the respective population simulations.

System-dependent parameters including reference concentrations and enzyme expression variabilities are listed in Table S3.

Table S3. System-dependent parameters and expression of relevant enzymes.

Enzyme / Processes	Mean reference concentration [µmol/L][a]	GeoSD of the reference concentration	Relative expression in different organs[b]	Half-life liver [hours]	Half-life intestine [hours]
**Enzymes**					
CYP2B6	1.56 [10]	1.40[c]	RT-PCR [11]	32	23
CYP2D6	0.40 [10]	0[d]	RT-PCR [11]	51	23
CYP3A4	4.32 [10]	1.18 (liver)[7]<br>1.45 (duodenum)[7]	RT-PCR [11]	36 [12]	23 [13]
**Processes**					
Unspec. hep. CL of (*E*)-4-OH-Clom	-	1.40[c]			
Unspec. hep. CL of (*E*)-4-OH-DE-Clom	-	1.40[c]			

CYP: cytochrome P450, **(*E*)-4-OH-Clom**: (*E*)-4-hydroxyclomiphene, **(*E*)-4-OH-DE-Clom**: (*E*)-4-hydroxy-N-desethylclomiphene, **GeoSD**: geometric standard deviation, **RT-PCR**: reverse transcription polymerase chain reaction, **unspec. hep. CL**: unspecific hepatic clearance

[a] [µmol protein/L] in the tissue of the highest expression

[b] PK-Sim® expression database profile

[c] geometric standard deviation with coefficient of variation (CV) of 35 % assumed

[d] as described in Section 1.2

S1.3. Supplementary Information on (*E*)-clomiphene PBPK Model Building

The parent-metabolite PBPK model of (E)-Clom was developed using a middle-out approach, combining information on drug- and system-specific parameters from literature with a parameter estimation step based on clinical trial data [14]. *In vitro*, *in silico* and clinical *in vivo* data were combined to inform model input parameters [14]. Information about absorption, distribution, metabolism and excretion (ADME) processes were used to incorporate relevant enzymes.

Metabolism via CYP enzymes was implemented as Michaelis-Menten kinetic processes. To account for nonspecific binding in *in vitro* assays, apparent Michaelis-Menten constant (K_m) values informed from literature were adjusted by the free fraction of drug compound as suggested by Obach and Austin et al. [15, 16]. K_m and v_{max} values were available only for composite metabolic pathway reactions, while parameters for each specific CYP enzyme involved in the respective pathway were not reported. When multiple CYP enzymes were incorporated in one metabolic pathway (see Figure 2 in the main manuscript), identical K_m values were allocated to each CYP enzyme and the corresponding k_{cat} estimated with a fixed ratio based on *in vitro* results on the metabolic enzyme activities [17, 18].

In the PBPK model, three metabolic pathways were implemented for the parent compound (E)-Clom: metabolism to (E)-DE-Clom, metabolism to (E)-4-OH-Clom and metabolism to (Z)-3-hydroxyclomiphene (implemented as an undefined metabolite). The latter enzymatic pathway, mediated via CYP2D6, was estimated with a 1.8-fold higher intrinsic clearance compared to the formation of (E)-4-OH-Clom in the PBPK model according to literature [19]. Further, the formation of (E)-DE-Clom is primarily catalyzed by CYP3A4 and to some extent by CYP2D6 [17, 18]. This was integrated by accounting for the 80:20 metabolic ratio of CYP3A4 to CYP2D6 reported by Mazzarino and coworkers [20]. (E)-DE-CLOM itself is also metabolized via CYP3A4 and CYP2D6 to (E)-N,N-didesethylclomiphene (implemented as an undefined metabolite) [17, 18]. As previously described, the ratio of the corresponding measured *in vitro* metabolic enzyme activities was used during the parameter estimation step for optimization of k_{cat} values ($k_{cat,\ CYP3A4} = 0.13 * k_{cat,\ CYP2D6}$) [17, 18].

S1.4. Drug-dependent Parameter Tables

Table S4. Drug-dependent parameters for (*E*)-clomiphene.

Parameter	Value	Unit	Source	Literature	Reference	Description
MW	405.96	g/mol	Literature	405.96	[22]	Molecular weight
pK_a (base)	9.31	-	Literature	9.31	[23]	Acid dissociation constant
Solubility (pH 6.8)	0.0138	mg/ml	Literature	0.0138	[24]	Solubility
logP	5.67	-	Optimized	5.18, 6.08, 6.48, 6.65	[23, 25–27]	Lipophilicity
f_u	0.08	%	Optimized	1.42[a]	[21]	Fraction unbound
CYP2D6 K_m → (*E*)-4-OH-Clom	0.13	µmol/l	Literature	0.13[b]	[19]	Michaelis-Menten constant
CYP2D6 k_{cat} → (*E*)-4-OH-Clom	306.4[c]	1/min	Optimized	-	-	Catalytic rate constant
CYP2D6 K_m → undef.	0.03	µmol/l	Literature	0.03[b]	[19]	Michaelis-Menten constant
CYP2D6 k_{cat} → undef.	130.4[c]	1/min	Optimized	-	-	Catalytic rate constant
CYP2B6 K_m → (*E*)-4-OH-Clom	0.60	µmol/l	Literature	0.60[b]	[17, 18]	Michaelis-Menten constant
CYP2B6 k_{cat} → (*E*)-4-OH-Clom	7.5	1/min	Optimized	-	-	Catalytic rate constant
CYP2D6 K_m → (*E*)-DE-Clom	0.78	µmol/l	Literature	0.78[b]	[17, 18]	Michaelis-Menten constant
CYP2D6 k_{cat} → (*E*)-DE-Clom	121.4[c]	1/min	Optimized	-	-	Catalytic rate constant
CYP3A4 K_m → (*E*)-DE-Clom	0.78	µmol/l	Literature	0.78[b]	[17, 18]	Michaelis-Menten constant
CYP3A4 k_{cat} → (*E*)-DE-Clom	45.0	1/min	Optimized	-	-	Catalytic rate constant
GFR fraction	0.92	-	Optimized	-	-	Fraction of filtered drug in the urine
EHC continuous fraction	1.00	-	Assumed	-	-	Fraction of bile continually released
Partition coefficients	Diverse[d]	-	Calculated	Schmitt	[28]	Cell to plasma partition coefficients
Cellular permeability	Diverse[d]	cm/min	Calculated	Ch. dep. Schmitt	[29]	Permeability into the cellular space
Intestinal permeability	0.08	cm/min	Optimized	-	-	Transcellular intestinal permeability
Tablet Weibull time	6.80	min	Assumed	-	[e]	Dissolution time (50 % dissolved)
Tablet Weibull shape	0.47	-	Assumed	-	[e]	Dissolution profile shape

Ch. dep. Schmitt: Charge dependent Schmitt calculation method, **CYP**: cytochrome P450, **(*E*)-4-OH-Clom**: (*E*)-4-hydroxyclomiphene, **(*E*)-DE-Clom**: (*E*)-N-desethylclomiphene, **EHC**: enterohepatic circulation, **GFR**: glomerular filtration rate, **IM**: intermediate metabolizers, **IVSF**: *in vitro* scaling factor, **NM**: normal metabolizers, **UM**: ultrarapid metabolizers, **undef.**: undefined metabolite

[a] f_u was estimated with the classification model by Watanabe et al. [21]

[b] K_m values from literature were adapted with the calculated $f_{u,inc}$=0.024, considering nonspecific binding in *in vitro* assays according to [15, 16]

[c] Only CYP2D6 k_{cat} values of NM are shown while IM- and UM-k_{cat} values were extrapolated according to Equation 1 in the main manuscript (IVSFs represented in Table S8)

[d] values differ across the organs

[e] see Section 1.6

Table S5. Drug-dependent parameters for (*E*)-N-desethylclomiphene.

Parameter	Value	Unit	Source	Literature	Reference	Description
MW	377.91	g/mol	Literature	377.91	[30]	Molecular weight
pK_a (base)	8.14	-	Optimized	9.59	[30]	Acid dissociation constant
Solubility (pH 6.5)	0.46	mg/ml	Literature	0.46	[30]	Solubility
logP	4.17	-	Optimized	5.74, 6.4	[30, 31]	Lipophilicity
f_u	0.86	%	Optimized	1.37[a]	[21]	Fraction unbound
CYP2D6 K_m → (*E*)-4-OH-DE-Clom	0.49	µmol/l	Literature	0.49[b]	[17, 18]	Michaelis-Menten constant
CYP2D6 k_{cat} → (*E*)-4-OH-DE-Clom	64.5[c]	1/min	Optimized	-	-	Catalytic rate conbstant
CYP2D6 K_m → undef.	0.97	µmol/l	Literature	0.97[b]	[17, 18]	Michaelis-Menten constant
CYP2D6 k_{cat} → undef.	5.8[c]	1/min	Optimized	-	-	Catalytic rate constant
CYP3A4 K_m → undef.	0.97	µmol/l	Literature	0.97[b]	[17, 18]	Michaelis-Menten constant
CYP3A4 k_{cat} → undef.	0.8	1/min	Optimized	-	-	Catalytic rate constant
GFR fraction	0.10	-	Optimized	-	-	Fraction of filtered drug in the urine
EHC continuous fraction	1.00	-	Assumed	-	-	Fraction of bile continually released
Partition coefficients	Diverse[d]	-	Calculated	R&R	[32, 33]	Cell to plasma partition coefficients
Cellular permeability	Diverse[d]	cm/min	Calculated	Ch. dep. Schmitt	[29]	Permeability into the cellular space

Ch. dep. Schmitt: Charge dependent Schmitt caclulation method, **CYP**: cytochrome P450,
(*E*)-4-OH-DE-Clom: (*E*)-4-hydroxy-N-desethylclomiphene, **EHC**: enterohepatic circulation, **GFR**: glomerular filtration rate,
IM: intermediate metabolizers, **IVSF**: *in vitro* scaling factor, **NM**: normal metabolizers, **R&R**: Rodgers and Rowland calculation method,
UM: ultrarapid metabolizers, **undef.**: undefined metabolite

[a] f_u was estimated with the classification model by Watanabe et al. [21]

[b] K_m values from literature were adapted with the calculated $f_{u,inc}$=0.059, considering nonspecific binding in *in vitro* assays according to [15, 16]

[c] Only CYP2D6 k_{cat} values of NM are shown while IM- and UM-k_{cat} values were extrapolated according to Equation 1 in the main manuscript (IVSFs represented in Table S8)

[d] values differ across the organs

Table S6. Drug-dependent parameters for (*E*)-4-hydroxyclomiphene.

Parameter	Value	Unit	Source	Literature	Reference	Description
MW	421.97	g/mol	Literature	421.97	[34]	Molecular weight
pK_a (acid)	8.64	-	Literature	8.64	[34]	Acid dissociation constant
pK_a (base)	7.90	-	Optimized	9.41	[34]	Acid dissociation constant
Solubility (pH 6.5)	0.06	mg/ml	Literature	0.06	[34]	Solubility
logP	5.50	-	Optimized	5.31, 5.64	[25, 34]	Lipophilicity
f_u	0.45	%	Optimized	0.6, 1.33[a]	[21, 35]	Fraction unbound
CYP2D6 K_m → undef.	3.60	µmol/l	Literature	3.60[b]	[19]	Michaelis-Menten constant
CYP2D6 k_{cat} → undef.	855.2[c]	1/min	Optimized	-	-	Catalytic rate constant
CYP3A4 K_m → (*E*)-4-OH-DE-Clom	3.40	µmol/l	Literature	3.40[b]	[17, 18]	Michaelis-Menten constant
CYP3A4 k_{cat} → (*E*)-4-OH-DE-Clom	19.5	1/min	Optimized	-	-	Catalytic rate constant
Unspec. hep. CL → undef.	23.78	1/min	Optimized	-	-	Elimination from plasma (first-order process in the liver)
GFR fraction	0.24	-	Optimized	-	-	Fraction of filtered drug in the urine
EHC continuous fraction	1.00	-	Assumed	-	-	Fraction of bile continually released
Partition coefficients	Diverse[d]	-	Calculated	Berez.	[36]	Cell to plasma partition coefficients
Cellular permeability	2.23	cm/min	Calculated	PK-Sim	[37]	Permeability into the cellular space

Berez.: Berezhkovskiy calculation method, **CYP**: cytochrome P450, **(*E*)-4-OH-DE-Clom**: (*E*)-4-hydroxy-N-desethylclomiphene, **EHC**: enterohepatic circulation, **GFR**: glomerular filtration rate, **IM**: intermediate metabolizers, **IVSF**: *in vitro* scaling factor, **NM**: normal metabolizers, **PK-Sim**: PK-Sim standard calculation method, **UM**: ultrarapid metabolizers, **undef.**: undefined metabolite, **unspec. hep. CL**: unspecific hepatic clearance

[a] f_u was estimated with the classification model by Watanabe et al. [21]

[b] K_m values from literature were adapted with the calculated $f_{u,inc}$=0.099, considering nonspecific binding in *in vitro* assays according to [15, 16]

[c] Only CYP2D6 k_{cat} values of NM are shown while IM- and UM-k_{cat} values were extrapolated according to Equation 1 in the main manuscript (IVSFs represented in Table S8)

[d] values differ across the organs

Table S7. Drug-dependent parameters for (*E*)-4-hydroxy-N-desethyl-clomiphene.

Parameter	Value	Unit	Source	Literature	Reference	Description
MW	393.91	g/mol	Literature	393.91	[38]	Molecular weight
pK_a (acid)	8.69	-	Literature	8.69	[38]	Acid dissociation constant
pK_a (base)	9.65	-	Literature	9.65	[38]	Acid dissociation constant
Solubility (pH 6.5)	0.17	mg/ml	Literature	0.17	[38]	Solubility
logP	3.71	-	Optimized	4.47	[38]	Lipophilicity
f_u	1.32	%	Calculated	1.32[a]	[21]	Fraction unbound
CYP2D6 K_m → undef.	8.86	µmol/l	Assumed	8.86[b,c]	-	Michaelis-Menten constant
CYP2D6 k_{cat} → undef.	211.7[d]	1/min	Optimized	-	-	Catalytic rate constant
Unsp. hep. CL → undef.	8.50	1/min	Optimized	-	-	Elimination from plasma (first-order process in the liver)
GFR fraction	0.13	-	Optimized	-	-	Fraction of filtered drug in the urine
EHC continuous fraction	1.00	-	Assumed	-	-	Fraction of bile continually released
Partition coefficients	Diverse[e]	-	Calculated	Schmitt	[28]	Cell to plasma partition coefficients
Cellular permeability	Diverse[e]	cm/min	Calculated	Ch. dep. Schmitt	[29]	Permeability into the cellular space

Ch. dep. Schmitt: Charge dependent Schmitt calculation method, **CYP**: cytochrome P450, **(*E*)-4-OH-Clom**: (*E*)-4-hydroxyclomiphene, **(*E*)-4-OH-DE-Clom**: (*E*)-4-hydroxy-N-desethylclomiphene, **EHC**: enterohepatic circulation, **GFR**: glomerular filtration rate, **IM**: intermediate metabolizers, **IVSF**: *in vitro* scaling factor, **NM**: normal metabolizers, **UM**: ultrarapid metabolizers, **undef.**: undefined metabolite, **unsp. hep. CL**: unspecific hepatic clearance

[a] f_u was estimated with the classification model by Watanabe et al. [21]

[b] K_m values from literature were adapted with the calculated $f_{u,inc}$=0.243, considering nonspecific binding in *in vitro* assays according to [15, 16]

[c] K_m value for CYP2D6-mediated hydroxylation of (*E*)-4-OH-DE-Clom was assumed to be equal to K_m value of the CYP2D6-mediated hydroxylation of (*E*)-4-OH-Clom

[d] Only CYP2D6 k_{cat} values of NM are shown while IM- and UM-k_{cat} values were extrapolated according to Equation 1 in the main manuscript (IVSFs represented in Table S8)

[e] values differ across the organs

S1.5. Calculation of Fractions Metabolized

The fraction metabolized (f_m) of (*E*)-Clom via CYP2D6 was calculated according to Equation S1, using the observed relative AUC_{last} increase between the PM population and the control group (normal metabolizers (NM)) [39]. Calculation yielded a CYP2D6 f_m of ~90%. In addition, data from the CYP2D6 NM population in the clarithromycin DDI scenario (CYP3A4 inhibition) was used to estimate f_m of (*E*)-Clom via CYP3A4 to inform model development regarding CYP3A4-dependent (*E*)-Clom degradation. For this, the observed relative AUC_{last} increase in the NM population between the DDI scenario with CYP3A4 inhibition and the control scenario without inhibition was used, yielding a CYP3A4 f_m of about 13%. Of note, a complete CYP3A4 inhibition through clarithromycin was assumed, given the strong and mechanism-based inhibition through clarithromycin, which was administered twice a day for four days before the victim drug, (*E*)-Clom, was administered.

$$\frac{1}{1 - f_m} = \frac{AUC_{last,effect,\,AS=i}}{AUC_{last,control}} \tag{S1}$$

In case of CYP2D6 f_m calculation, $AUC_{last,\,effect}$ represents the AUC_{last} of (*E*)-Clom for the PM population, while $AUC_{last,\,control}$ represents the AUC_{last} of (*E*)-Clom for the NM population. For

calculation of the CYP3A4 f_m, $AUC_{last,\ effect}$ represents the AUC_{last} of (E)-Clom for the NM population in the DDI scenario with clarithromycin, while $AUC_{last,\ control}$ represents the AUC_{last} of (E)-Clom for the NM population without concomitant clarithromycin administration.

S1.6. Formulations

Dissolution profiles for clomiphene citrate tablets and (E)-Clom citrate capsules were not publicly available. However, according to the U.S. pharmacopoeia, the dissolution rate within the first 30 minutes of clomiphene citrate tablets is required to be at least 75% [40]. This information was used to inform the dissolution shape and time (50% dissolved) parameters of a Weibull function, which was employed as the formulation in PK-Sim® (mathematical implementation see Equation S2 and Equation S3). The respective parameter values are represented in Table S4.

$$m = 1 - exp\left(\frac{-(t - T_{lag})^{\beta}}{\alpha}\right) \tag{S2}$$

$$with\ \alpha = (T_d)^{\beta} \tag{S3}$$

Here, m represents the fraction of dissolved drug at time t, T_{lag} is the lag time before onset of dissolution, α is the scaling parameter, β the shape parameter and T_d the time needed to dissolve 63% of the formulation [37].

S1.7. Handling Data Below the Lower Limit of Quantification (LLOQ)

In the pharmacokinetic panel study used for model building and evaluation, 9% of measured concentrations fell below the lower limit of quantification (LLOQ). For handling lower limit of quantification (LLOQ) data, a combination of the M5 and M6 method [41] was used. Below limit of quantification (BLQ) individual plasma concentrations were substituted by LLOQ/2. Subsequently, mean concentrations were calculated for each CYP2D6 activity score (AS) and only the first BLQ data was used for model building and evaluation, while subsequent concentrations were excluded. During the initial period of metabolite formation, BLQ data also appeared in the ascending branch of the plasma profiles. In this case, the last BLQ concentration was included in the data, while BLQ concentrations before this time point were discarded.

S2. Drug-Gene-Interaction (DGI) Modeling

S2.1. CYP2D6 *in vitro* Scaling Factors

The estimated CYP2D6 k_{cat} values for the NM population were extrapolated to the intermediate metabolizers (IM) (AS=0.5, AS=0.75 and AS=1) and ultrarapid metabolizers (UM) populations according to Equation 1 in the main manuscript, using *in vitro* scaling factors (IVSFs). Determination of IVSFs were based on AS-specific *in vitro* metabolite formation rates relative to the corresponding formation rate in NM as a reference. The respective IVSFs for each CYP2D6-dependent pathway are depicted in Table S8. Measured *in vitro* data for (*E*)-4-OH-Clom and (*E*)-4-OH-DE-Clom AS-specific formation rates were available, while mean values were assumed for the remaining CYP2D6-dependent metabolic pathways [17].

Table S8. Employed *in vitro* scaling factors (IVSFs) for individual CYP2D6 activity scores.

CYP2D6-mediated metabolic pathways	AS=0	AS=0.5	AS=0.75	AS=1	AS=2	AS=3
(*E*)-Clom → (*E*)-4-OH-Clom	0	0.19	0.27	0.57	1	1.52
(*E*)-Clom → (*E*)-DE-Clom	0	0.17	0.23	0.51	1	1.41
(*E*)-Clom → undef.	0	0.17	0.23	0.51	1	1.41
(*E*)-4-OH-Clom → undef.	0	0.17	0.23	0.51	1	1.41
(*E*)-4-OH-DE-Clom → undef.	0	0.17	0.23	0.51	1	1.41
(*E*)-DE-Clom → (*E*)-4-OH-DE-Clom	0	0.16	0.19	0.44	1	1.30
(*E*)-DE-Clom → undef.	0	0.17	0.23	0.51	1	1.41

AS: CYP2D6 activity score, **CYP**: cytochrome P450, **(*E*)-4-OH-Clom**: (*E*)-4-hydroxyclomiphene, **(*E*)-4-OH-DE-Clom**: (*E*)-4-hydroxy-N-desethylclomiphene, **(*E*)-Clom**: (*E*)-clomiphene, **(*E*)-DE-Clom**: (*E*)-N-desethylclomiphene, **undef.**: undefined metabolite

S3. Drug-Drug-(Gene)-Interaction (DD(G)I) Modeling

S3.1. Clarithromycin and Paroxetine

Clarithromycin acts as a mechanism-based inhibitor of CYP3A4, while paroxetine inhibits CYP2D6 and to a minor extent CYP3A4 [42]. Inhibition mechanisms of CYP3A4 and CYP2D6 were implemented according to the PK-Sim® manual [37]. Two previously published PBPK models of clarithromycin [43] and paroxetine [44] were applied and coupled with the developed parent-metabolite PBPK model of (*E*)-Clom to assess the model prediction performance in the DD(G)I setting. Interaction parameters were used as published in the respective perpetrator PBPK models.

S4. PBPK Model Evaluation

S4.1. Evaluation of the DGI Model

S4.1.1. Plasma Profiles (Linear Scale)

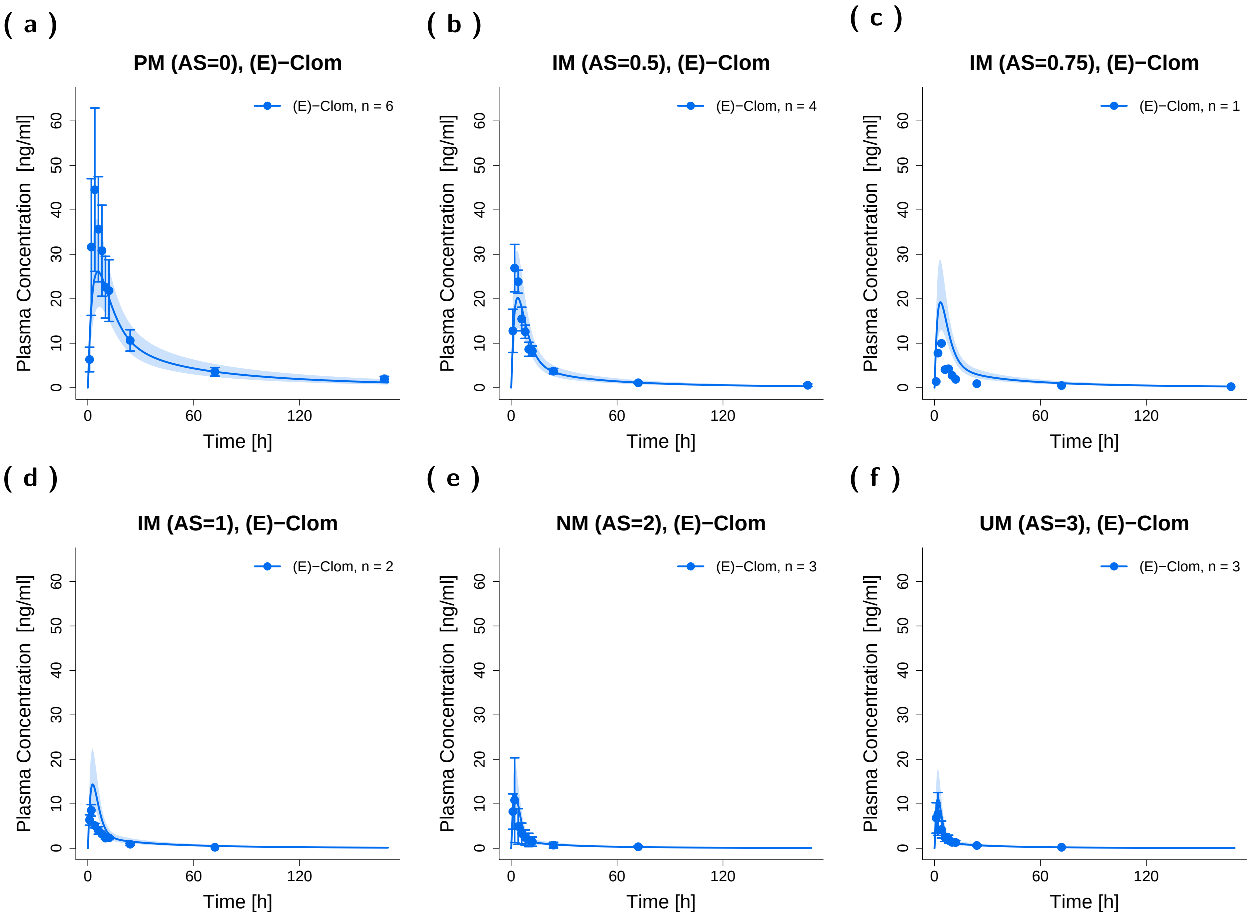

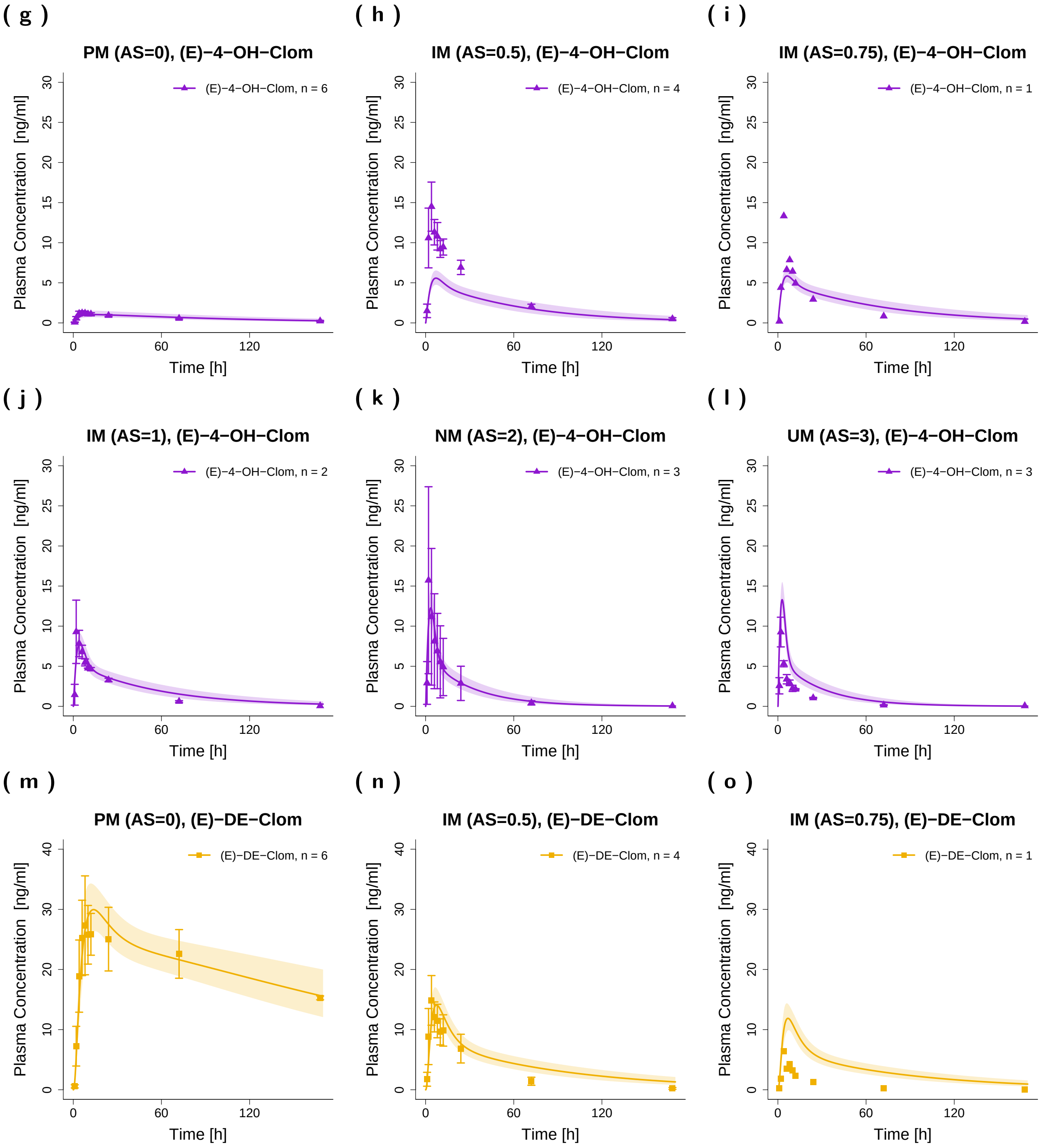

(g)
PM (AS=0), (E)−4−OH−Clom
(E)−4−OH−Clom, n = 6
(h)
IM (AS=0.5), (E)−4−OH−Clom
(E)−4−OH−Clom, n = 4
(i)
IM (AS=0.75), (E)−4−OH−Clom
(E)−4−OH−Clom, n = 1
(j)
IM (AS=1), (E)−4−OH−Clom
(E)−4−OH−Clom, n = 2
(k)
NM (AS=2), (E)−4−OH−Clom
(E)−4−OH−Clom, n = 3
(l)
UM (AS=3), (E)−4−OH−Clom
(E)−4−OH−Clom, n = 3
(m)
PM (AS=0), (E)−DE−Clom
(E)−DE−Clom, n = 6
(n)
IM (AS=0.5), (E)−DE−Clom
(E)−DE−Clom, n = 4
(o)
IM (AS=0.75), (E)−DE−Clom
(E)−DE−Clom, n = 1
Plasma Concentration [ng/ml]
Time [h]
0
60
120

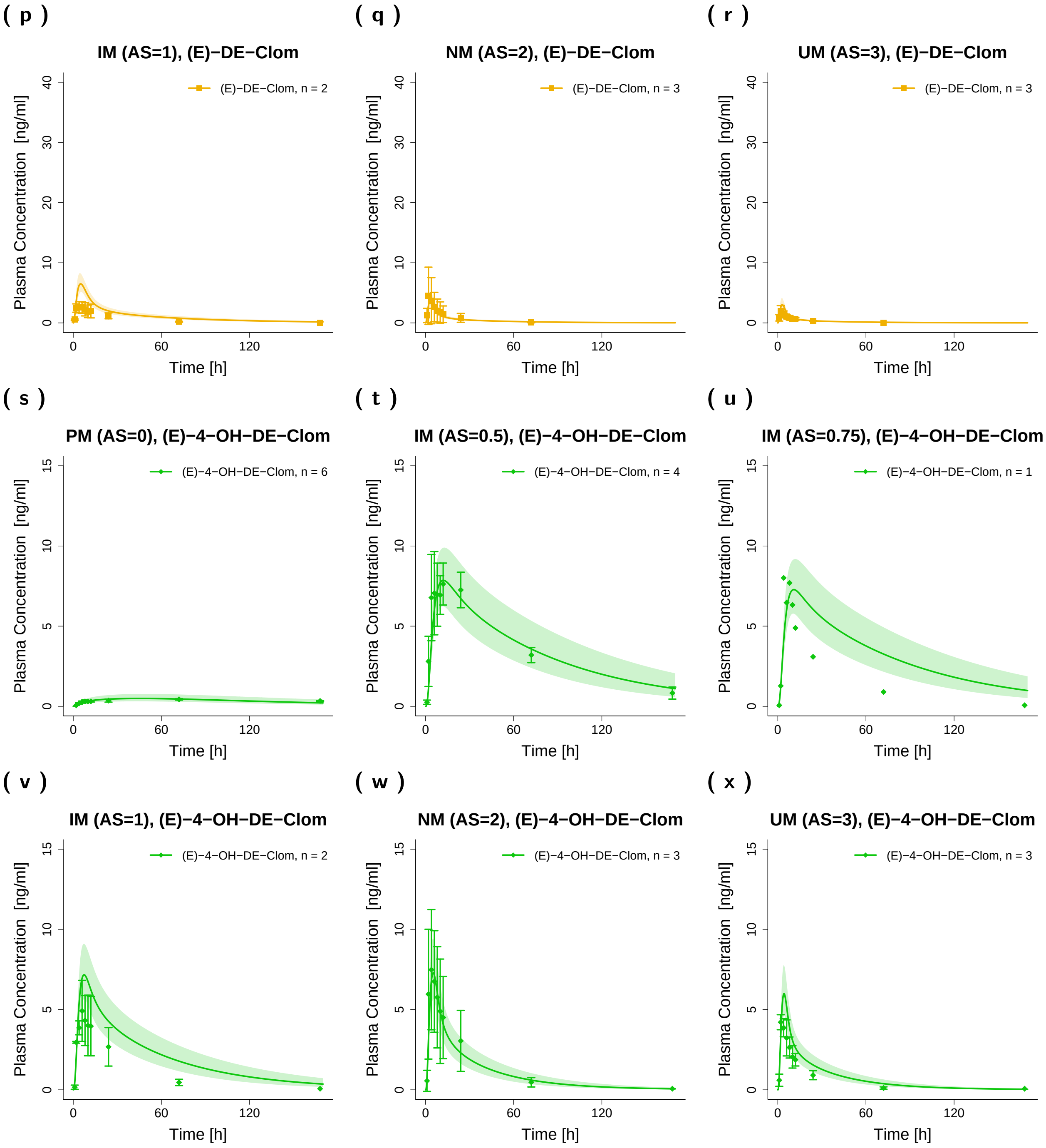

Figure S1. Predicted and observed plasma concentration-time profiles (linear scale) of (E)-Clom (a–f), (*E*)-4-OH-Clom (g–l), (*E*)-DE-Clom (m–r) and (*E*)-4-OH-DE-Clom (s–x) for DGI scenarios. Solid lines de-pict predicted geometric mean concentration-time profiles in PM, IM, NM and UM. The respective semitranspar-ent areas show the geometric standard deviation of the population simulations (n=1000). Mean observed data are shown as symbols with the corresponding standard deviation. **AS**, CYP2D6 activity score; **DGI**, drug-gene inter-action; **(*E*)-4-OH-Clom**, (*E*)-4-hydroxyclomiphene; **(*E*)-4-OH-DE-Clom**, (*E*)-4-hydroxy-N-desethylclomiphene;**(*E*)-Clom**, (*E*)-clomiphene; **(*E*)-DE-Clom**, (*E*)-N-desethylclomiphene; **IM**, intermediate metabolizers; **n**, num-ber of subjects; **NM**, normal metabolizers; **PM**, poor metabolizers; **UM**, ultrarapid metabolizers.

S4.1.2. Plasma Profiles (Semilogarithmic Scale)

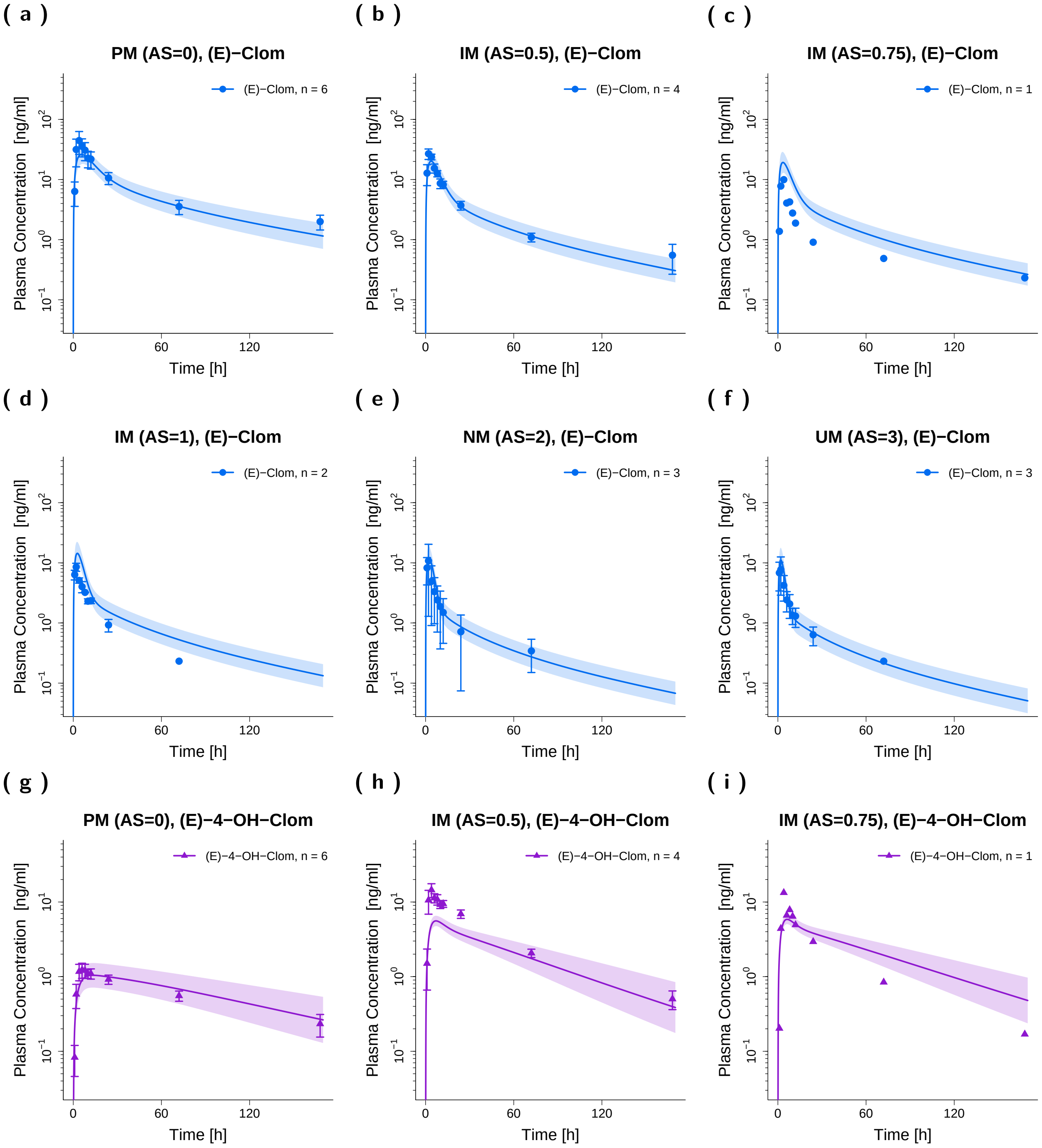

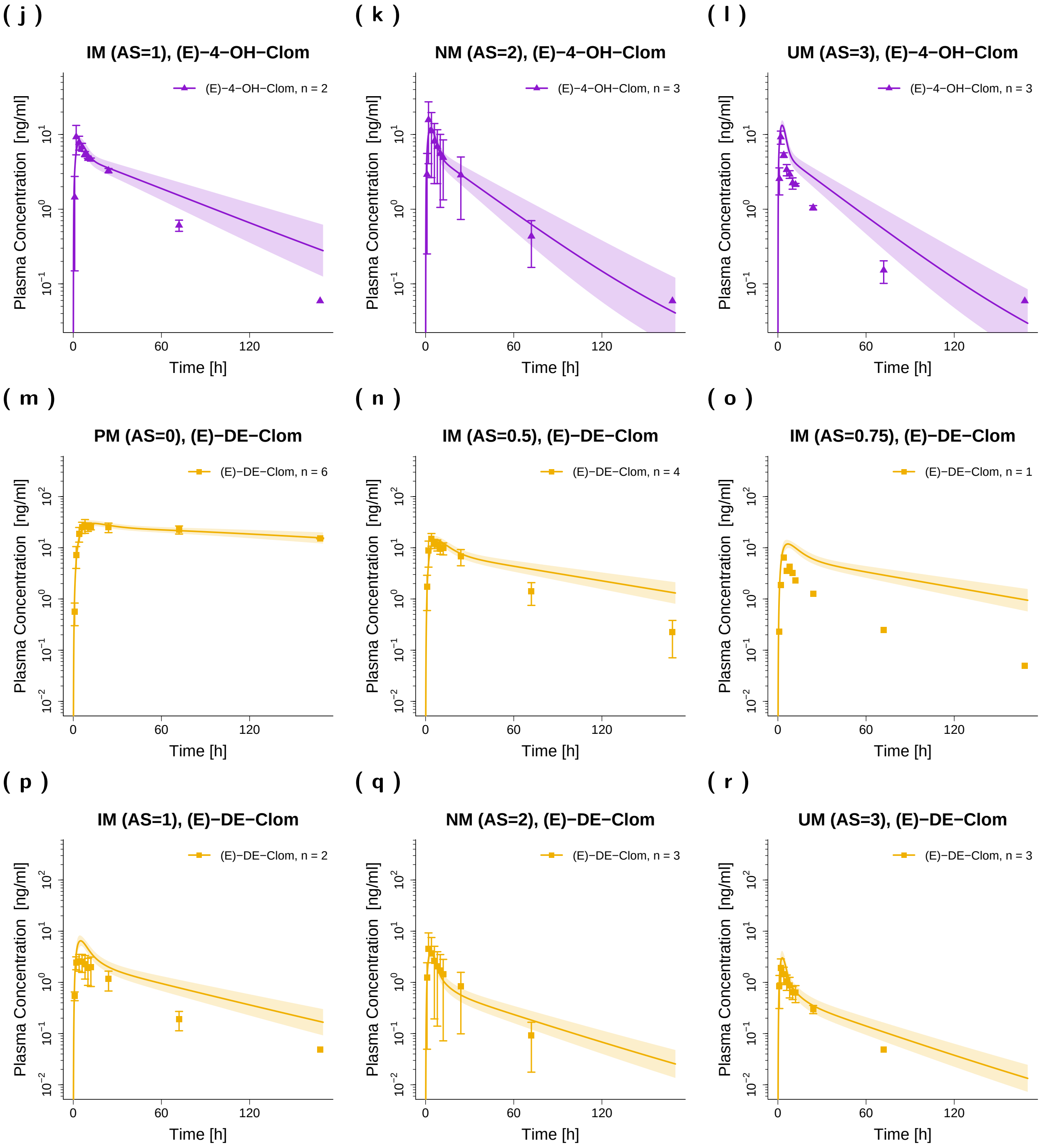
(j)
IM (AS=1), (E)−4−OH−Clom
(E)−4−OH−Clom, n = 2
(k)
NM (AS=2), (E)−4−OH−Clom
(E)−4−OH−Clom, n = 3
(l)
UM (AS=3), (E)−4−OH−Clom
(E)−4−OH−Clom, n = 3
(m)
PM (AS=0), (E)−DE−Clom
(E)−DE−Clom, n = 6
(n)
IM (AS=0.5), (E)−DE−Clom
(E)−DE−Clom, n = 4
(o)
IM (AS=0.75), (E)−DE−Clom
(E)−DE−Clom, n = 1
(p)
IM (AS=1), (E)−DE−Clom
(E)−DE−Clom, n = 2
(q)
NM (AS=2), (E)−DE−Clom
(E)−DE−Clom, n = 3
(r)
UM (AS=3), (E)−DE−Clom
(E)−DE−Clom, n = 3
Plasma Concentration [ng/ml]
Time [h]
0
60
120

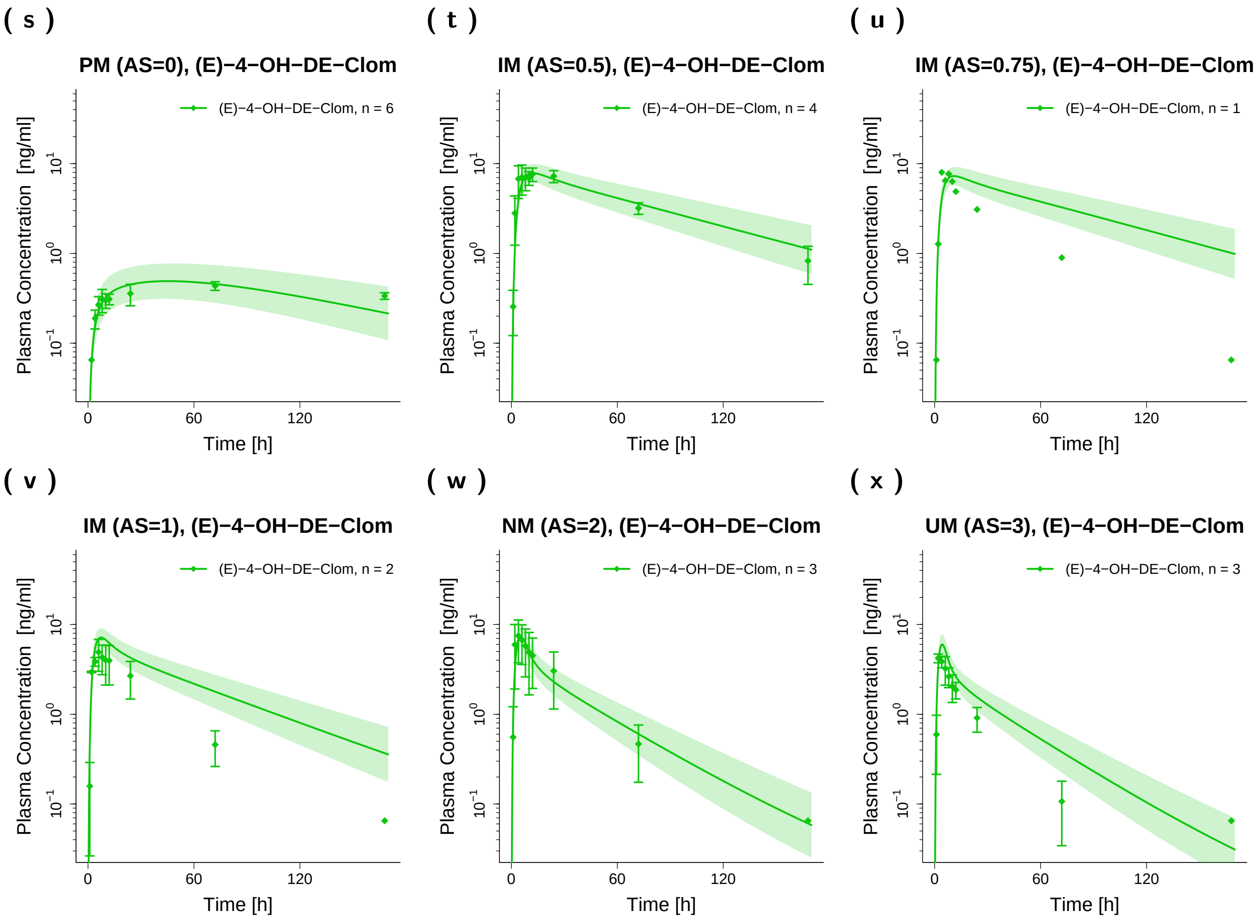

Figure S2. Predicted and observed plasma concentration-time profiles (semilogarithmic scale) of (*E*)-Clom (a–f), (*E*)-4-OH-Clom (g–l), (*E*)-DE-Clom (m–r) and (*E*)-4-OH-DE-Clom (s–x) for DGI scenarios. Solid lines depict predicted geometric mean concentration-time profiles in the PM, IM, NM and UM populations. The respective semitransparent areas show the geometric standard deviation of the population simulations (n=1000). Mean observed data are shown as symbols with the corresponding standard deviation. **AS**, CYP2D6 activity score; **DGI**, drug-gene interaction; **(*E*)-4-OH-Clom**, (*E*)-4-hydroxyclomiphene; **(*E*)-4-OH-DE-Clom**, (*E*)-4-hydroxy-N-desethylclomiphene; (E)-Clom, (E)-clomiphene; (E)-DE-Clom, (E)-N-desethylclomiphene; IM, intermediate metabolizers; n, number of subjects; NM, normal metabolizers; PM, poor metabolizers; UM, ultrarapid metab-olizers.

S4.1.3. Goodness-of-Fit Plots

(a) AUC_{last}

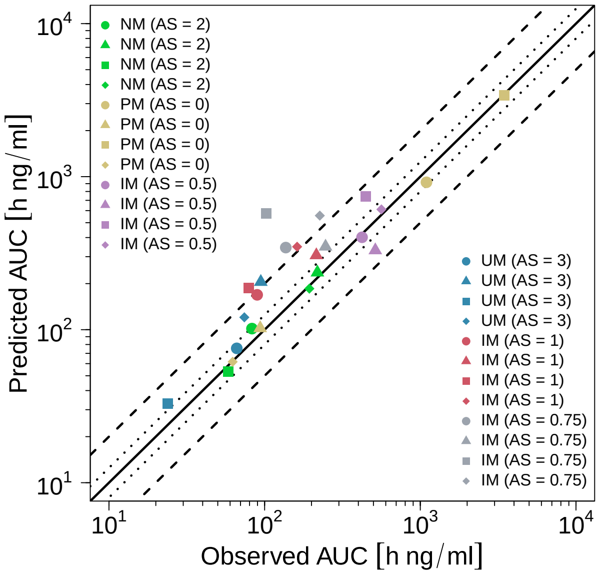

(b) C_{max}

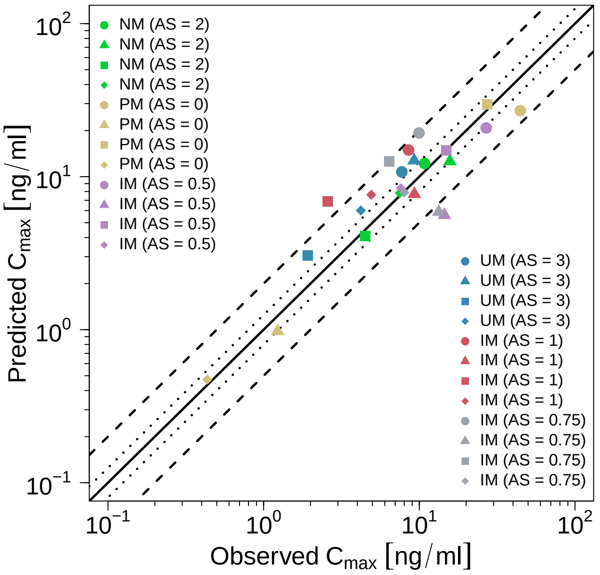

(c) Plasma concentrations

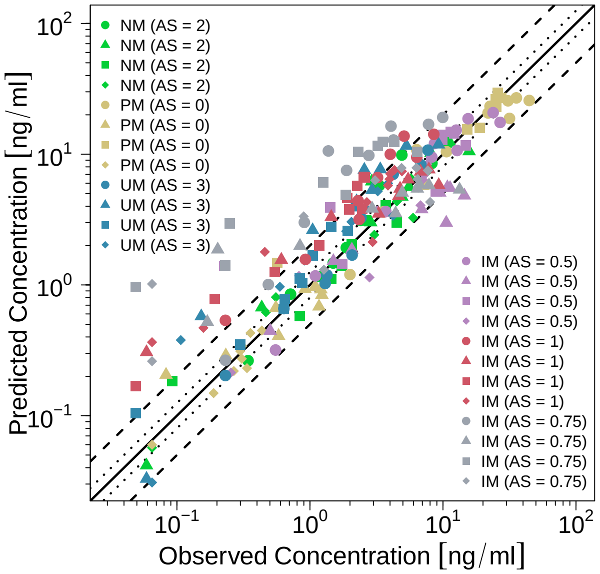

Figure S3. Predicted versus observed AUClast (a), Cmax (b) and plasma concentrations (c) of (E)-Clom (circles), (*E*)-4-OH-Clom (triangles), (*E*)-DE-Clom (squares) and (*E*)-4-OH-DE-Clom (diamonds) in PM, IM, NM and UM (DGI scenarios). The black solid lines mark the lines of identity. Black dotted lines indicate 1.25-fold, black dashed lines indicate 2-fold deviation. **AS**, CYP2D6 activity score; **DGI**, drug-gene interaction;**(*E*)-4-OH-Clom**, (*E*)-4-hydroxyclomiphene; **(*E*)-4-OH-DE-Clom**, (*E*)-4-hydroxy-N-desethylclomiphene; **(*E*)-Clom**, (*E*)-clomiphene; **(*E*)-DE-Clom**, (*E*)-N-desethylclomiphene; **IM**, intermediate metabolizers; **NM**, normal metabolizers; **PM**, poor metabolizers; **UM**, ultrarapid metabolizers.

(a) AUC_{last}

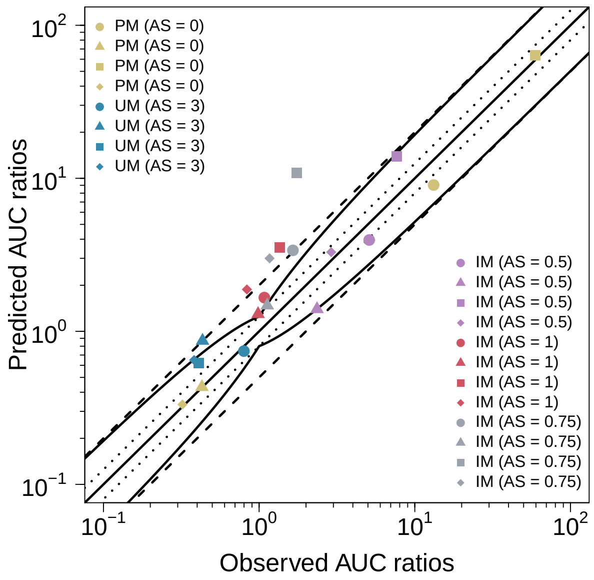

(b) C_{max}

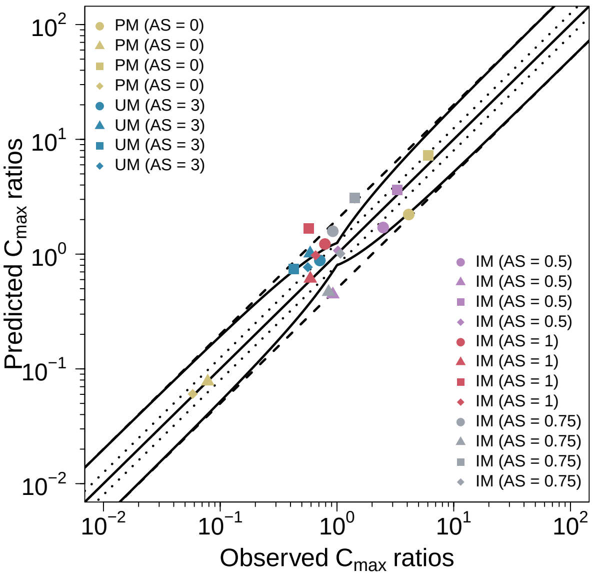

Figure S4. Predicted versus observed DGI AUC_{last} (a) and C_{max} (b) ratios of (*E*)-Clom (circles), (*E*)-4-OH-Clom (tri-angles), (*E*)-DE-Clom (squares) and (*E*)-4-OH-DE-Clom (diamonds) in PM, IM and UM. The straight black lines mark the lines of identity, the curved solid black lines show the limits of the predictive measure proposed by Guest et al. with 1.25-fold variability [46]. Black dotted lines indicate 1.25-fold, black dashed lines indicate 2-fold deviation. **AS**, CYP2D6 activity score; **(*E*)-4-OH-Clom**, (*E*)-4-hydroxyclomiphene; **(*E*)-4-OH-DE-Clom**, (*E*)-4-hydroxy-N-desethylclomiphene; **(*E*)-Clom**, (*E*)-clomiphene; **(*E*)-DE-Clom**, (E)-N-desethylclomiphene; **IM**, intermediate metabolizers; **PM**, poor metabolizers; **UM**, ultrarapid metabolizers.

S4.1.4. Renal Excretion Profiles (Linear Scale)

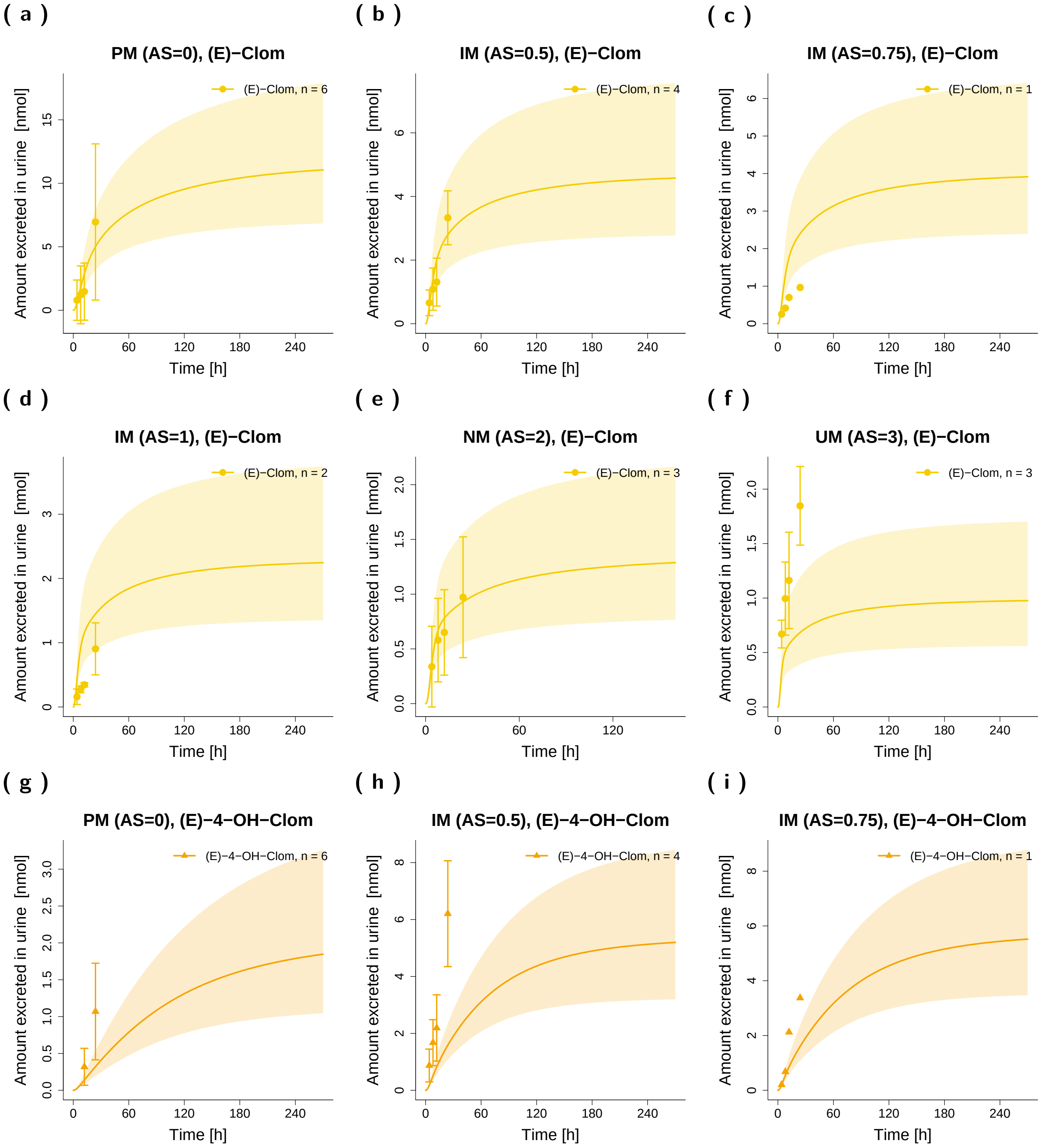

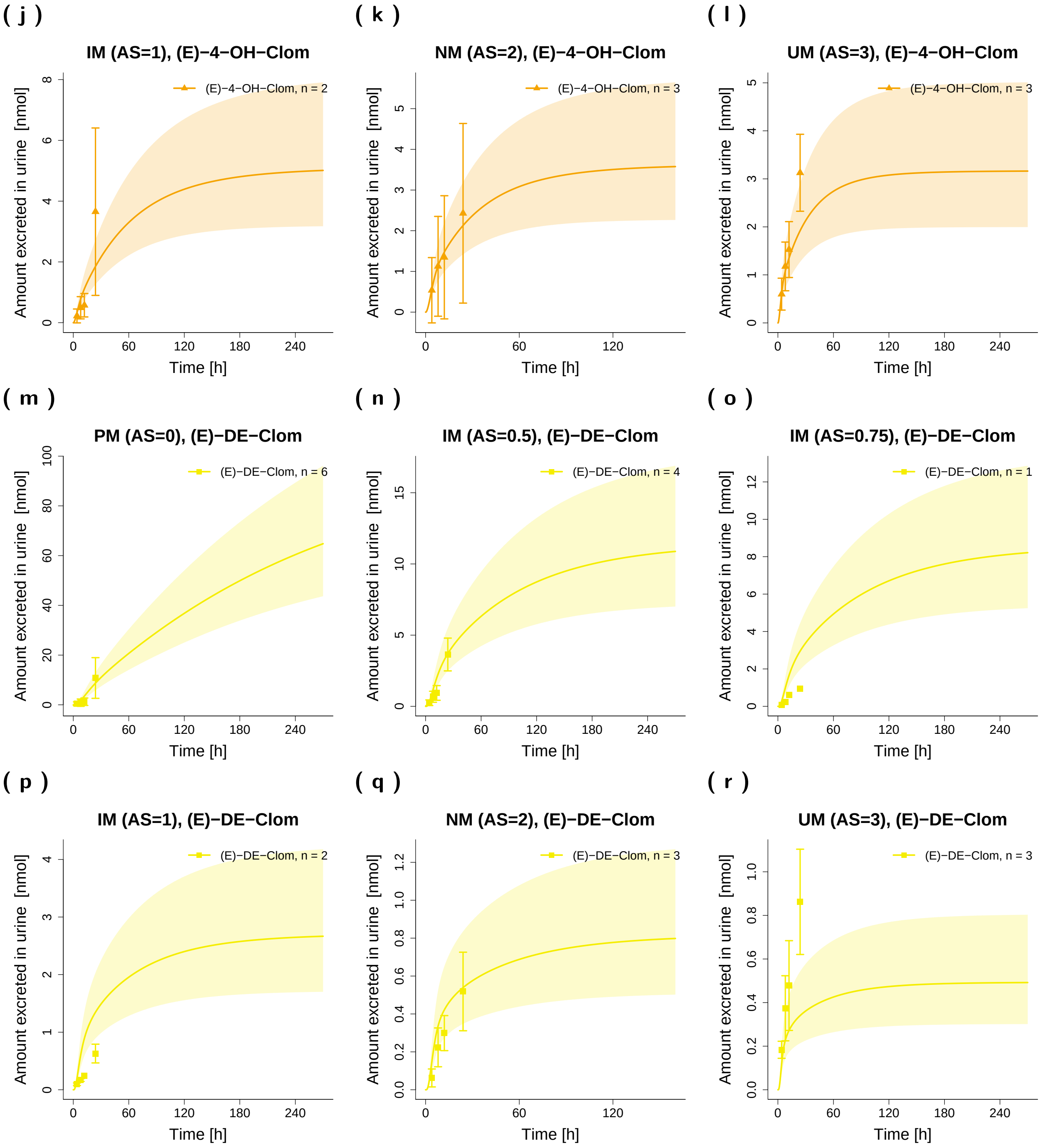
(j)
IM (AS=1), (E)−4−OH−Clom
(E)−4−OH−Clom, n = 2
(k)
NM (AS=2), (E)−4−OH−Clom
(E)−4−OH−Clom, n = 3
(l)
UM (AS=3), (E)−4−OH−Clom
(E)−4−OH−Clom, n = 3
(m)
PM (AS=0), (E)−DE−Clom
(E)−DE−Clom, n = 6
(n)
IM (AS=0.5), (E)−DE−Clom
(E)−DE−Clom, n = 4
(o)
IM (AS=0.75), (E)−DE−Clom
(E)−DE−Clom, n = 1
(p)
IM (AS=1), (E)−DE−Clom
(E)−DE−Clom, n = 2
(q)
NM (AS=2), (E)−DE−Clom
(E)−DE−Clom, n = 3
(r)
UM (AS=3), (E)−DE−Clom
(E)−DE−Clom, n = 3
Amount excreted in urine [nmol]
Time [h]

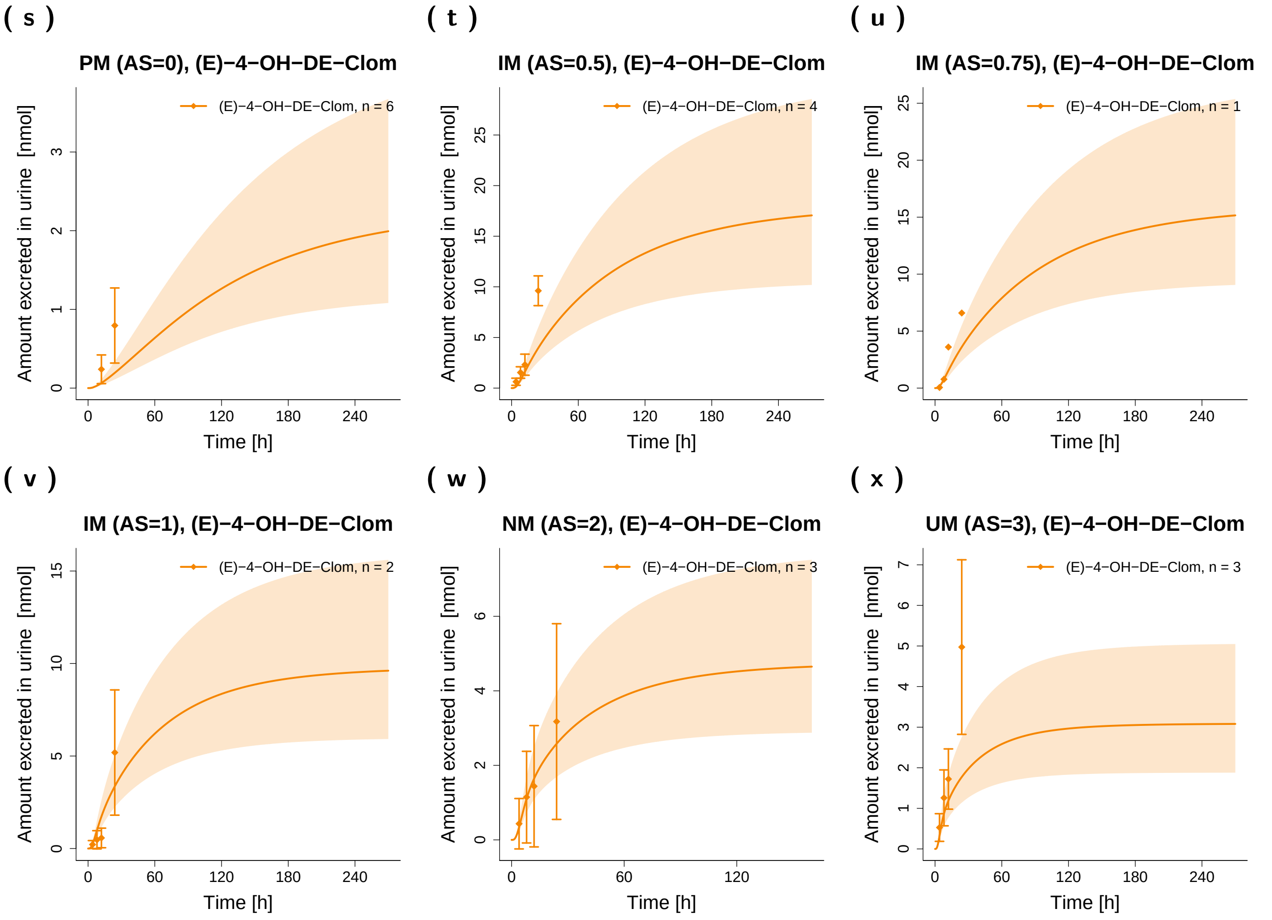

Figure S5. Predicted and observed renal excretion profiles (linear scale) of (E)-Clom (a–f), (E)-4-OH-Clom (g–l), (*E*)-DE-Clom (m–r) and (*E*)-4-OH-DE-Clom (s–x) for DGI scenarios. Solid lines depict predicted geometric mean profiles in PM, IM, NM and UM. The respective semitransparent areas show the geometric standard deviation of the population simulations (n=1000). Mean observed data are shown as symbols with the corresponding standard deviation. **AS**, CYP2D6 activity score; **DGI**, drug-gene interaction; **(*E*)-4-OH-Clom**, (*E*)-4-hydroxyclomiphene; **(*E*)-4-OH-DE-Clom**, (*E*)-4-hydroxy-N-desethylclomiphene; **(*E*)-Clom**, (*E*)-clomiphene; **(*E*)-DE-Clom**, (*E*)-N-desethylclomiphene; **IM**, intermediate metabolizers; **n**, number of subjects; **NM**, normal metabolizers, **PM**, poor metabolizers; **UM**, ultrarapid metabolizers.

S4.1.5. Plasma Profiles from Literature (Linear Scale)

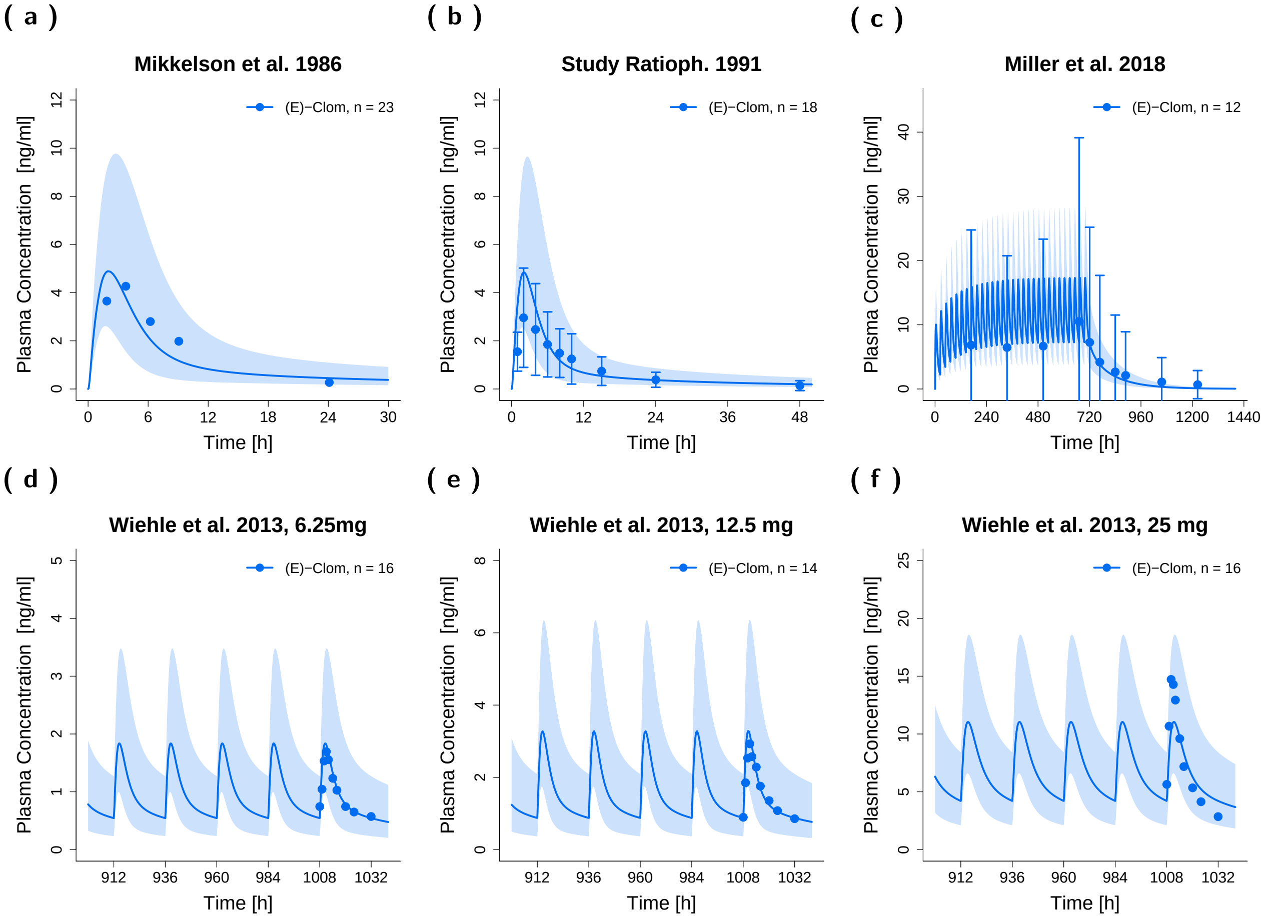

Figure S6. Predicted and observed plasma concentration-time profiles (linear scale) of digitized studies from literature after single (a,b) and multiple (c–f) dosing. Solid lines depict predicted geometric mean concentration-time profiles of (*E*)-Clom. The respective semitransparent areas show the geometric standard deviation of the population simulations (n=1000). Mean observed data are shown as symbols with the corresponding standard deviation. **(*E*)-Clom**, (*E*)-clomiphene; **n**, number of subjects; **Ratioph.**, Ratiopharm® GmbH.

S4.1.6. Plasma Profiles from Literature (Semilogarithmic Scale)

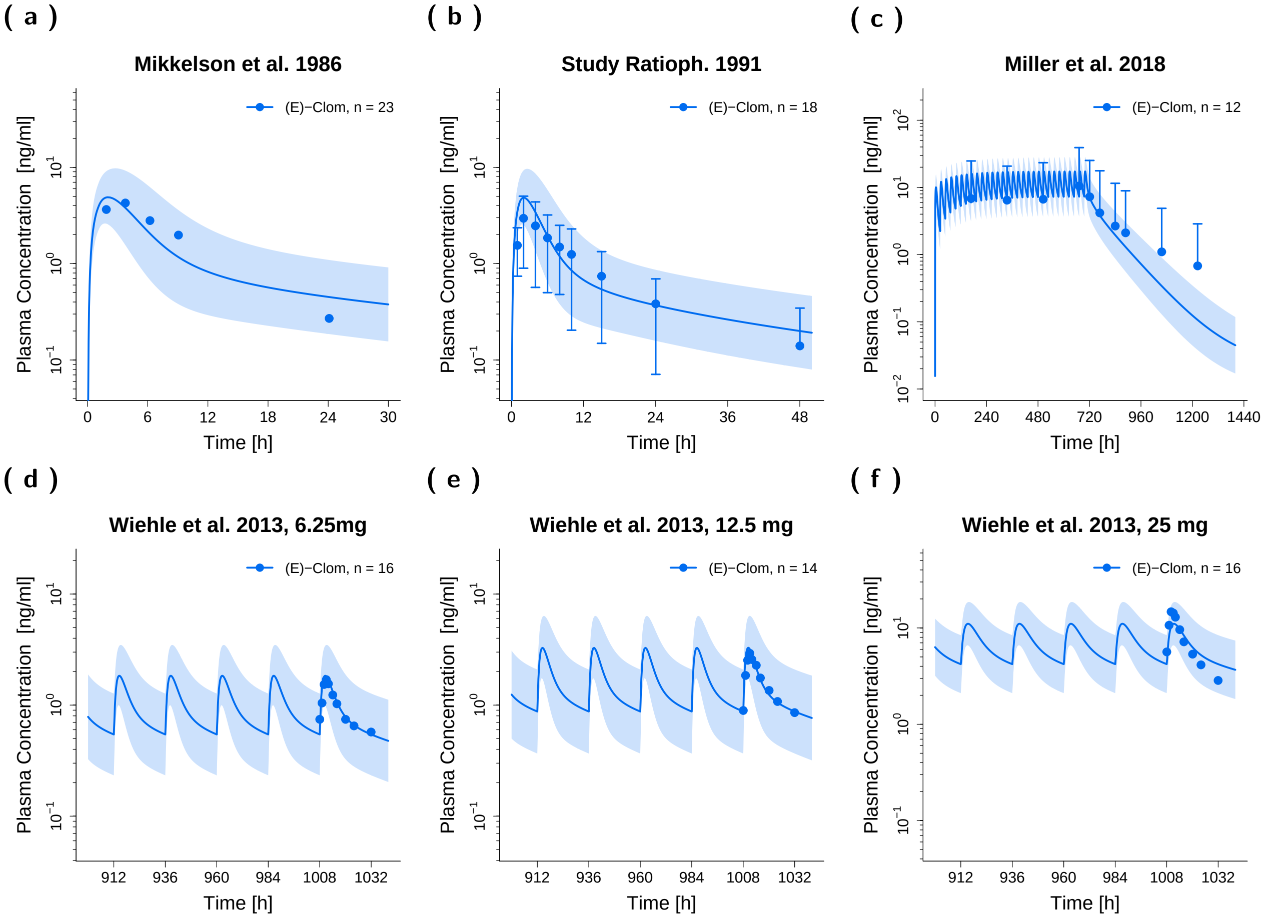

Figure S7. Predicted and observed plasma concentration-time profiles (semilogarithmic scale) of digitized studies from literature after single (a,b) and multiple (c–f) dosing. Solid lines depict predicted geometric mean concentration-time profiles of (*E*)-Clom. The respective semitransparent areas show the geometric standard deviation of the population simulations (n=1000). Mean observed data are shown as symbols with the corresponding standard deviation. **(*E*)-Clom**, (*E*)-clomiphene; **n**, number of subjects; **Ratioph.**, Ratiopharm® GmbH.

S4.1.7. Goodness-of-Fit Plots (from Literature)

(a) AUC_{last}

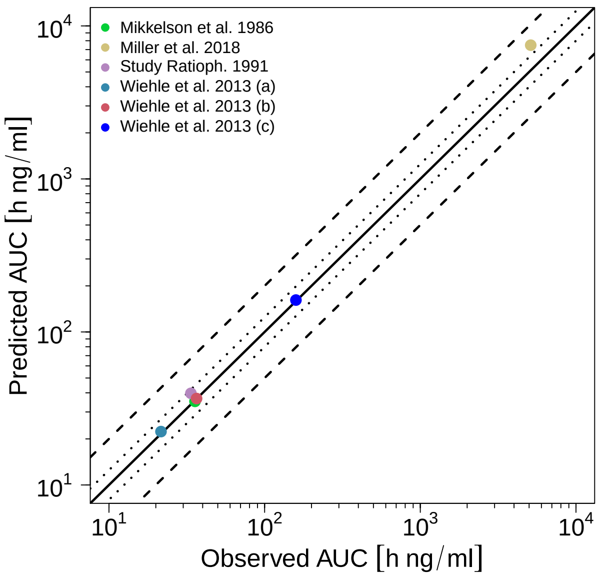

(b) C_{max}

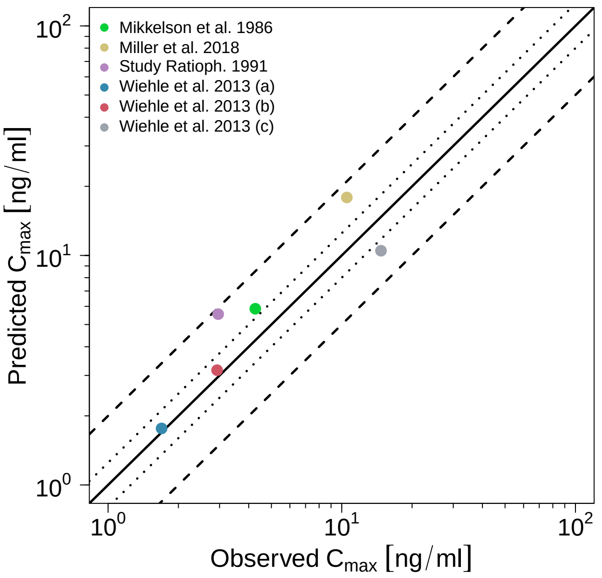

(c) Plasma concentrations

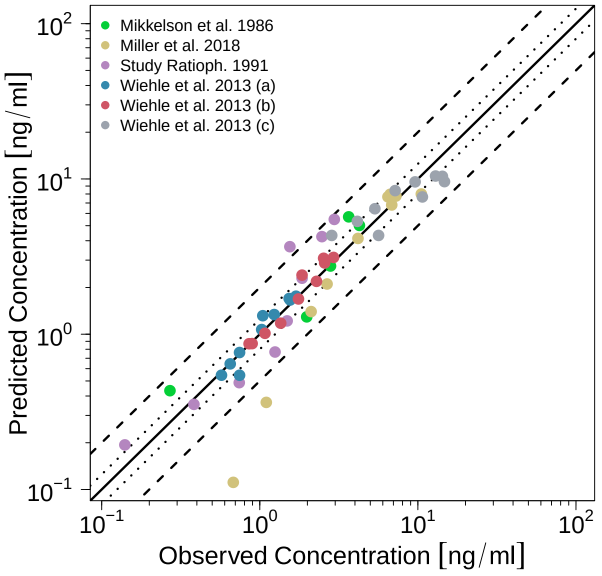

Figure S8. Predicted versus observed (a) AUC_{last}, (b) C_{max} and (c) plasma concentrations of (*E*)-Clom. The black solid lines mark the lines of identity. Black dotted lines indicate 1.25-fold, black dashed lines indicate 2-fold deviation. **Ratioph.**, Ratiopharm® GmbH.

S4.2. Evaluation of the DD(G)I Model

S4.2.1. Plasma Profiles (Linear Scale)

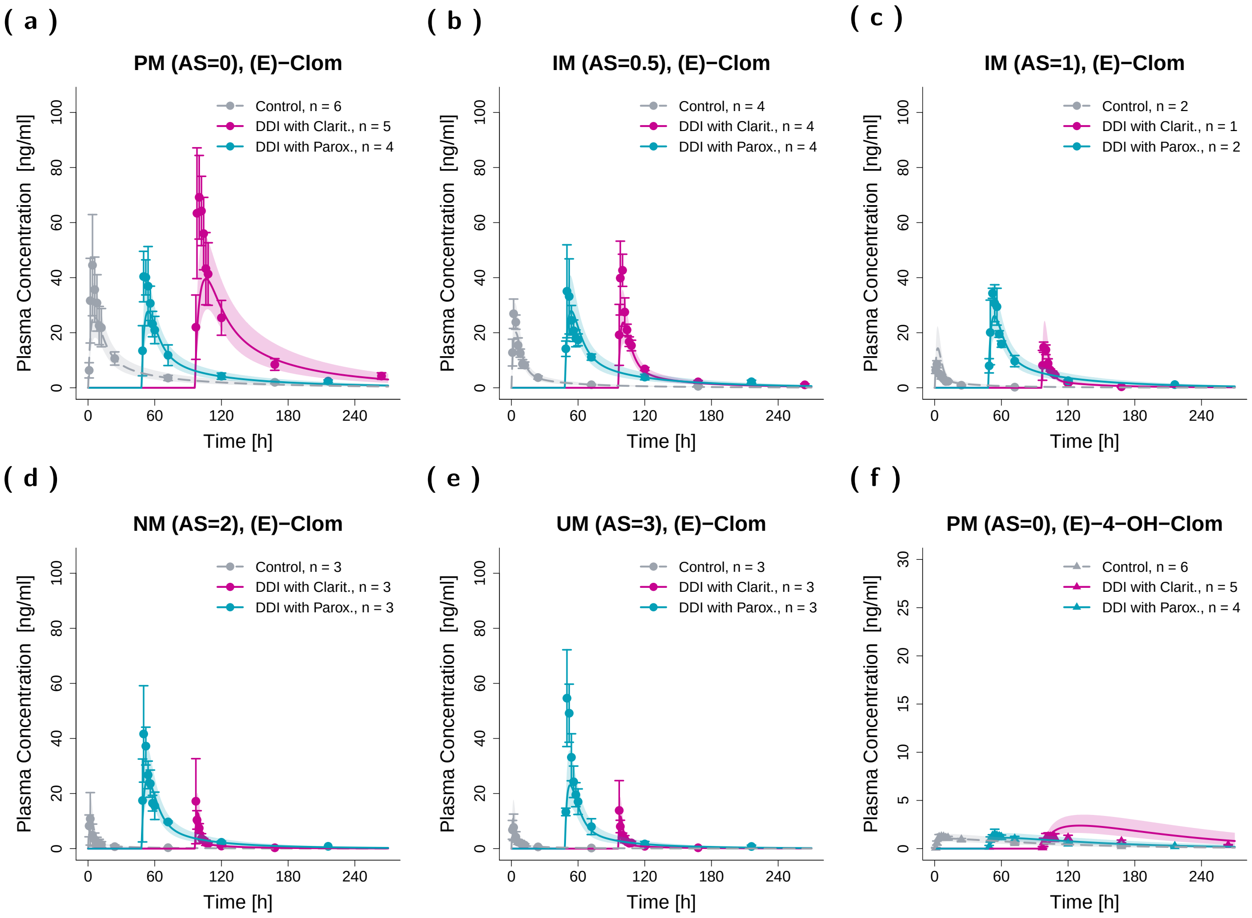

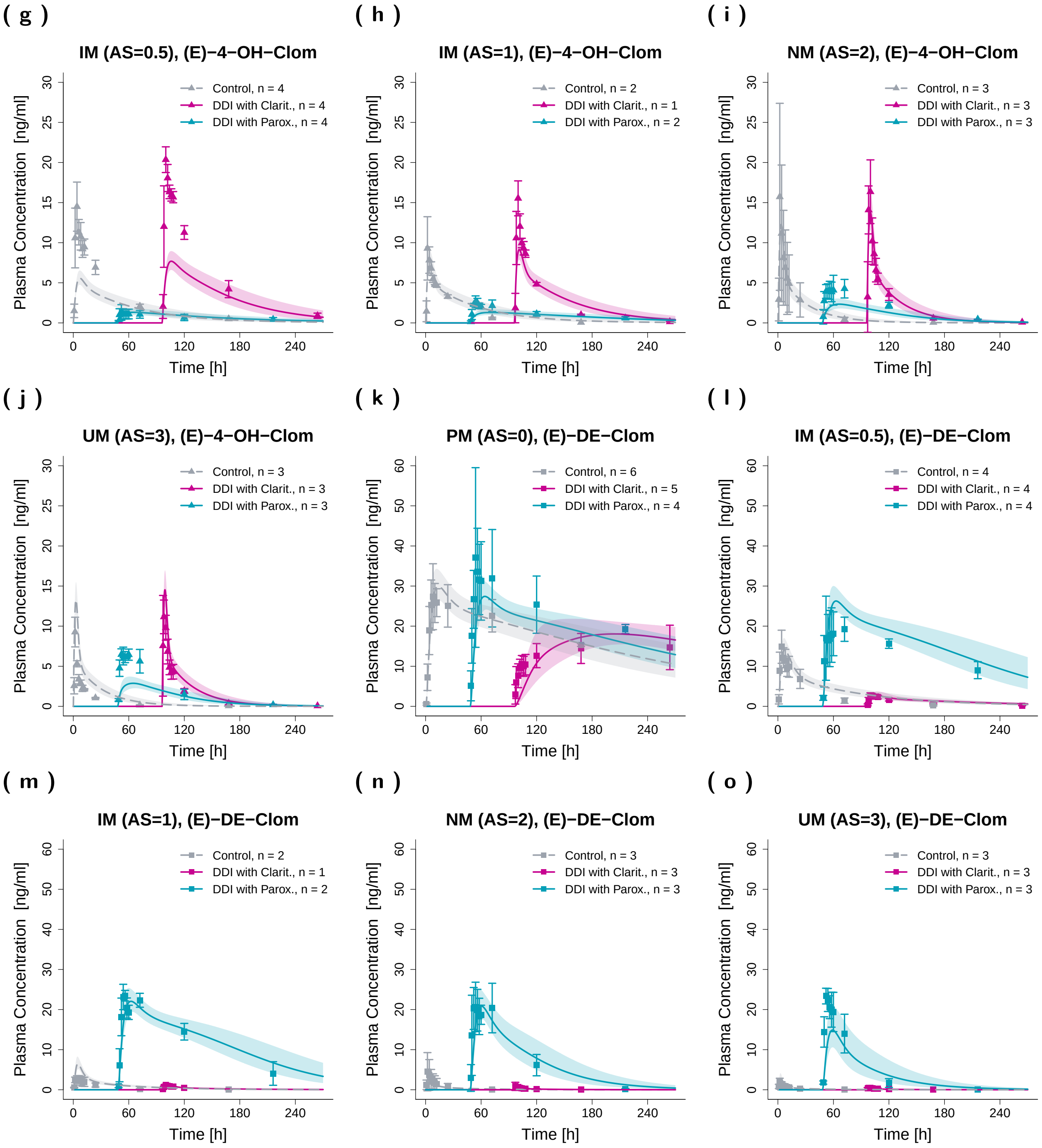
(g)
IM (AS=0.5), (E)−4−OH−Clom
Control, n = 4
DDI with Clarit., n = 4
DDI with Parox., n = 4
(h)
IM (AS=1), (E)−4−OH−Clom
Control, n = 2
DDI with Clarit., n = 1
DDI with Parox., n = 2
(i)
NM (AS=2), (E)−4−OH−Clom
Control, n = 3
DDI with Clarit., n = 3
DDI with Parox., n = 3
(j)
UM (AS=3), (E)−4−OH−Clom
Control, n = 3
DDI with Clarit., n = 3
DDI with Parox., n = 3
(k)
PM (AS=0), (E)−DE−Clom
Control, n = 6
DDI with Clarit., n = 5
DDI with Parox., n = 4
(l)
IM (AS=0.5), (E)−DE−Clom
Control, n = 4
DDI with Clarit., n = 4
DDI with Parox., n = 4
(m)
IM (AS=1), (E)−DE−Clom
Control, n = 2
DDI with Clarit., n = 1
DDI with Parox., n = 2
(n)
NM (AS=2), (E)−DE−Clom
Control, n = 3
DDI with Clarit., n = 3
DDI with Parox., n = 3
(o)
UM (AS=3), (E)−DE−Clom
Control, n = 3
DDI with Clarit., n = 3
DDI with Parox., n = 3
Plasma Concentration [ng/ml]
Time [h]
0
60
120
180
240

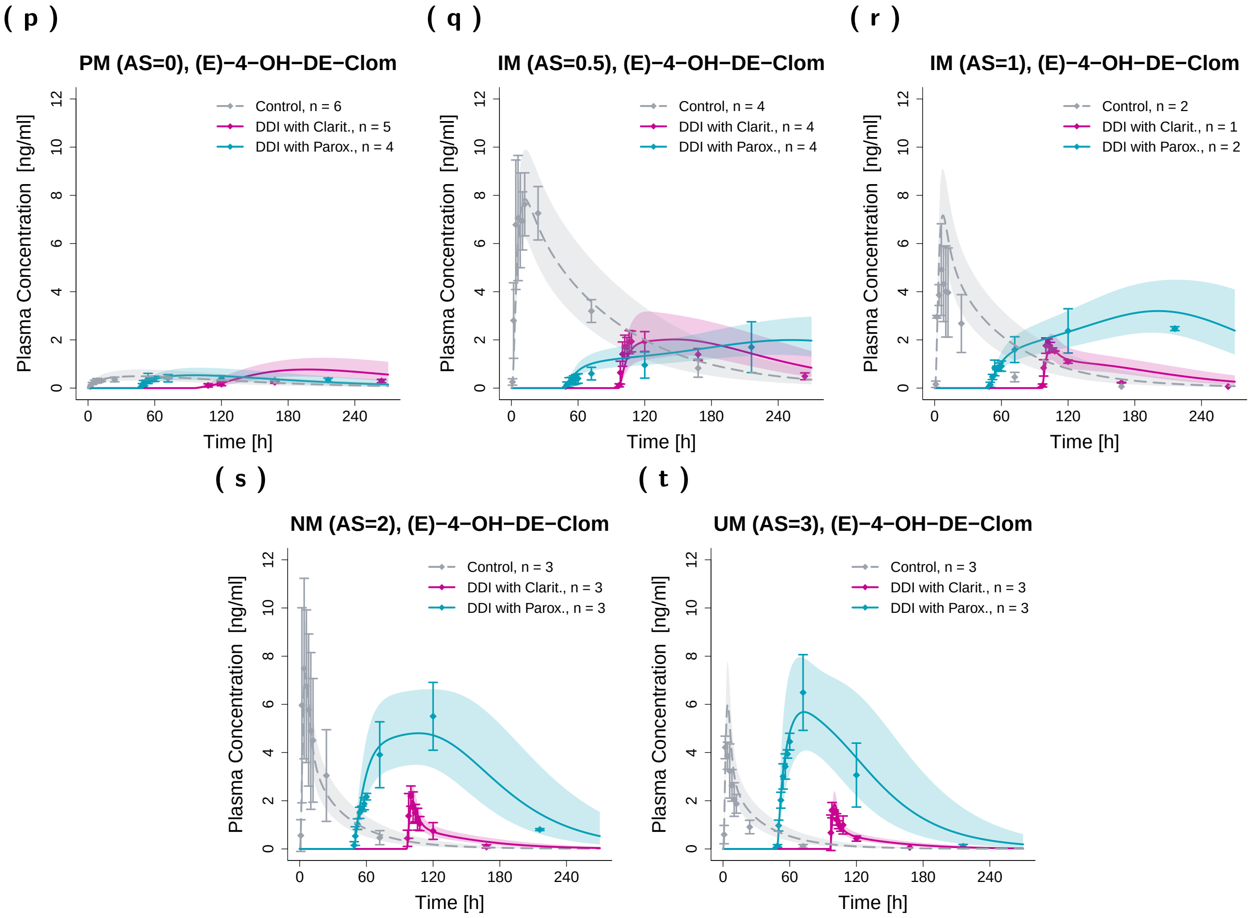

Figure S9. Predicted and observed plasma concentration-time profiles (linear scale) of (*E*)-Clom (a–e), (*E*)-4-OH-Clom (f–j), (*E*)-DE-Clom (k–o) and (*E*)-4-OH-DE-Clom (p–t) for DD(G)I scenarios in PM, IM, NM and UM. Grey dashed lines depict the predicted geometric mean concentration-time profiles without clarithromycin and paroxetine (control), turquoise lines represent the predicted geometric mean profiles in presence of paroxetine and pink lines the predicted geometric mean profiles in presence of clarithromycin (DD(G)I). The respective semitransparent areas show the geometric standard deviation of the population simulations (n=1000). Mean observed data are shown as symbols with the corresponding standard deviation. For a better visibility, DD(G)I scenarios were plotted with a time offset with t =0 at the first dose of the perpetrator drug. **AS**, CYP2D6 activity score; **Clarit.**, clarithromycin; **DD(G)I**, drug-drug and drug-drug-gene interactions; **(*E*)-4-OH-Clom**, (*E*)-4-hydroxyclomiphene; **(*E*)-4-OH-DE-Clom**, (*E*)-4-hydroxy-N-desethylclomiphene; **(*E*)-Clom**, (*E*)-clomiphene; **(*E*)-DE-Clom**, (*E*)-N-desethylclomiphene; **IM**, intermediate metabolizers; **n**, number of subjects; **NM**, normal metabolizers; **Parox.**, paroxetine; **PM**, poor metabolizers; **UM**, ultrarapid metabolizers.

S4.2.2. Plasma Profiles (Semilogarithmic Scale)

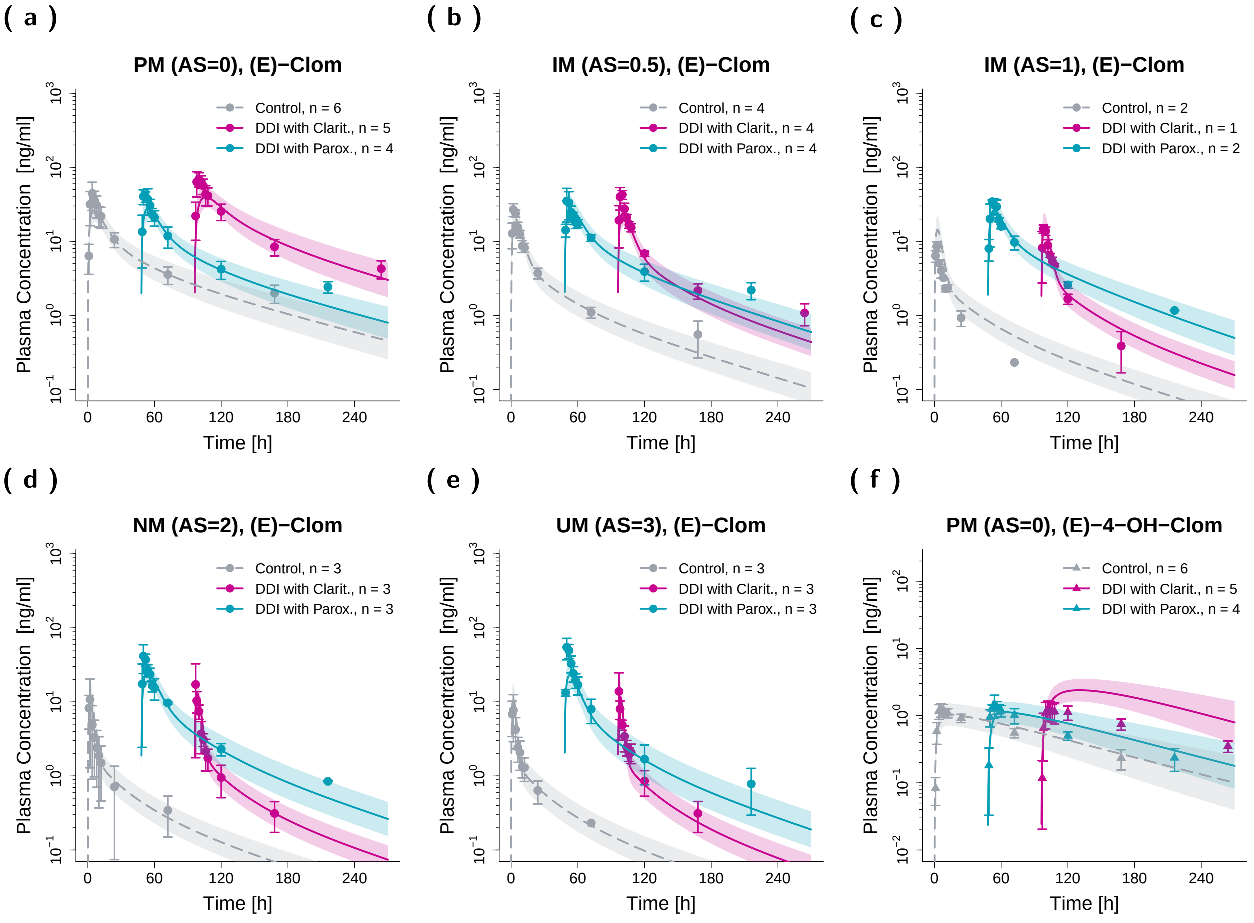

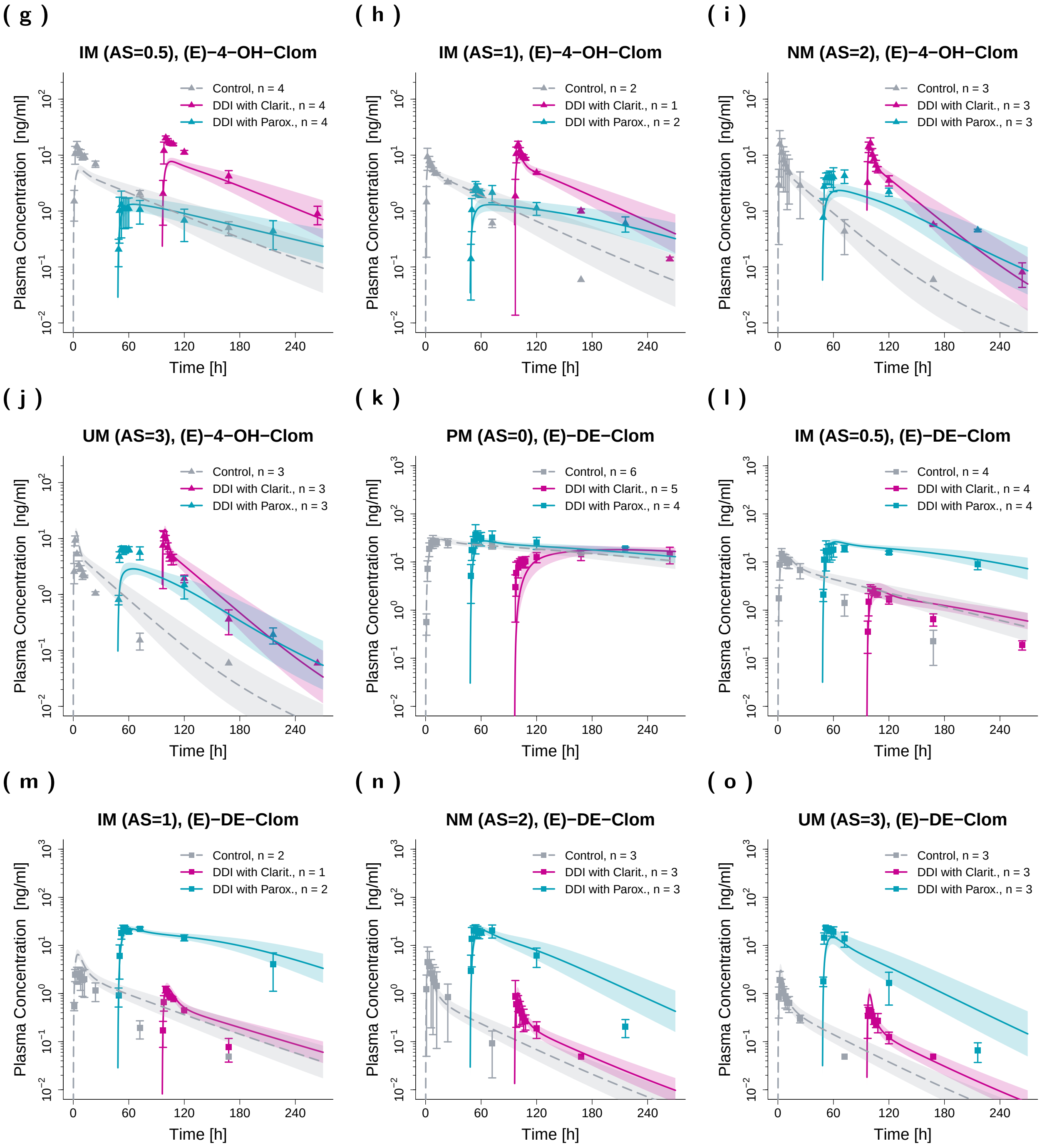
(g)
IM (AS=0.5), (E)−4−OH−Clom
Control, n = 4
DDI with Clarit., n = 4
DDI with Parox., n = 4
(h)
IM (AS=1), (E)−4−OH−Clom
Control, n = 2
DDI with Clarit., n = 1
DDI with Parox., n = 2
(i)
NM (AS=2), (E)−4−OH−Clom
Control, n = 3
DDI with Clarit., n = 3
DDI with Parox., n = 3
(j)
UM (AS=3), (E)−4−OH−Clom
Control, n = 3
DDI with Clarit., n = 3
DDI with Parox., n = 3
(k)
PM (AS=0), (E)−DE−Clom
Control, n = 6
DDI with Clarit., n = 5
DDI with Parox., n = 4
(l)
IM (AS=0.5), (E)−DE−Clom
Control, n = 4
DDI with Clarit., n = 4
DDI with Parox., n = 4
(m)
IM (AS=1), (E)−DE−Clom
Control, n = 2
DDI with Clarit., n = 1
DDI with Parox., n = 2
(n)
NM (AS=2), (E)−DE−Clom
Control, n = 3
DDI with Clarit., n = 3
DDI with Parox., n = 3
(o)
UM (AS=3), (E)−DE−Clom
Control, n = 3
DDI with Clarit., n = 3
DDI with Parox., n = 3
Plasma Concentration [ng/ml]
Time [h]
0
60
120
180
240

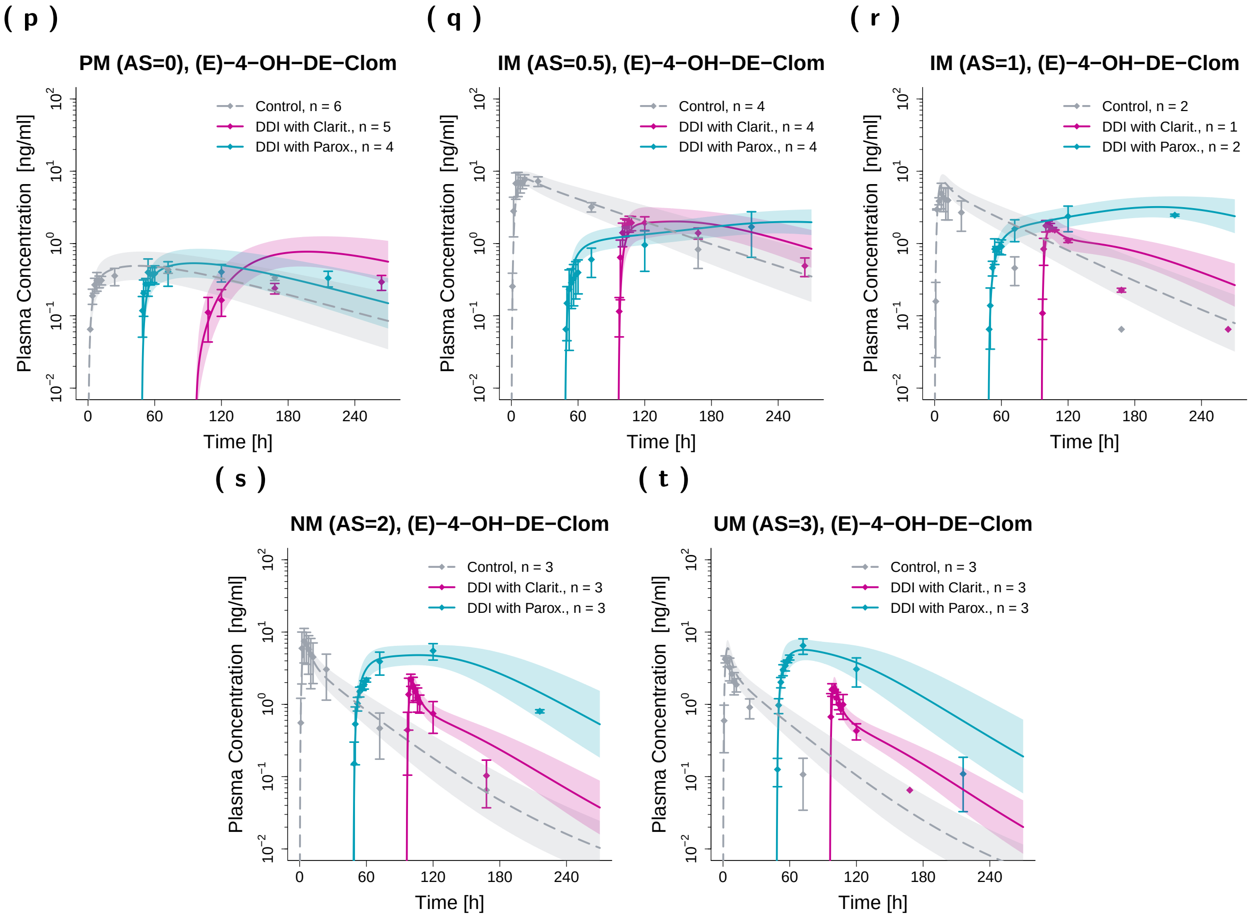

Figure S10. Predicted and observed plasma concentration-time profiles (semilogarithmic scale) of (*E*)-Clom (a–e), (*E*)-4-OH-Clom (f–j), (*E*)-DE-Clom (k–o) and (*E*)-4-OH-DE-Clom (p–t) for DD(G)I scenarios in PM, IM, NM and UM. Grey dashed lines depict the predicted geometric mean concentration-time profiles without clarithromycin and paroxetine (control), turquoise lines represent the predicted geometric mean profiles in presence of paroxetine and pink lines the predicted geometric mean profiles in presence of clarithromycin. The respective semitransparent areas show the geometric standard deviation of the population simulations (n=1000). Mean observed data are shown as symbols with the corresponding standard deviation. For a better visibility, DD(G)I scenarios were plotted with a time offset with t=0 at the first dose of the perpetrator drug. **AS**, CYP2D6 activity score; **Clarit.**, clarithromycin; **DD(G)I**, drug-drug and drug-drug-gene interactions; **(*E*)-4-OH-Clom**, (*E*)-4-hydroxyclomiphene; **(*E*)-4-OH-DE-Clom**, (*E*)-4-hydroxy-N-desethylclomiphene; **(*E*)-Clom**, (*E*)-clomiphene; **(*E*)-DE-Clom**, (*E*)-N-desethylclomiphene; **IM**, intermediate metabolizers; **n**, number of subjects; **NM**, normal metabolizers; **Parox.**, paroxetine; **PM**, poor metabolizers; **UM**, ultrarapid metabolizers.

S4.2.3. Goodness-of-Fit Plots

(a) AUC_{last}

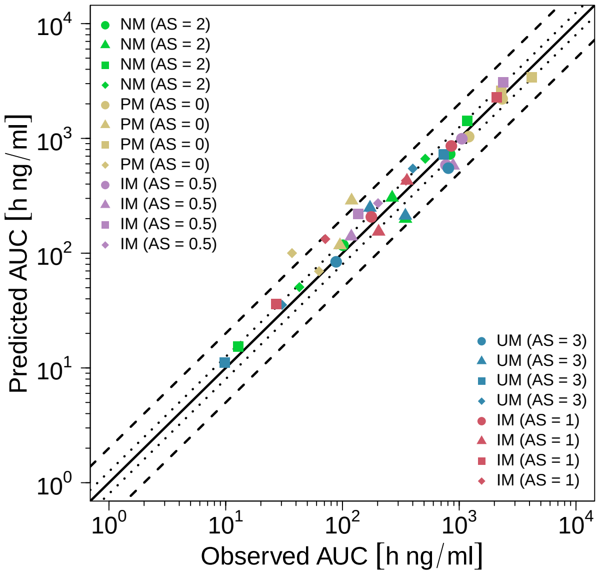

(b) C_{max}

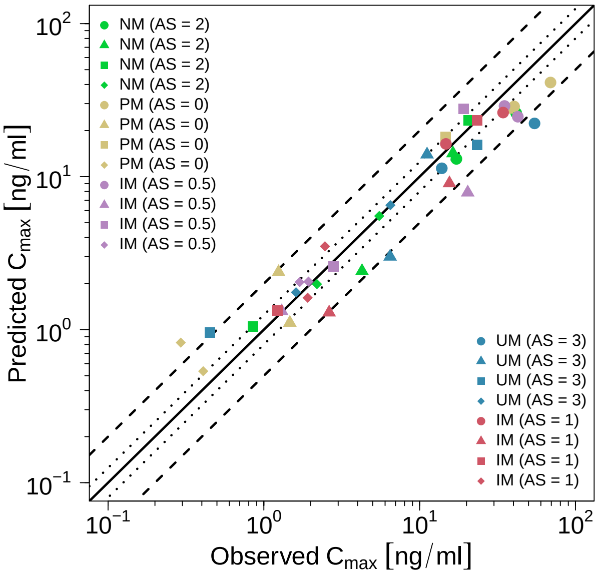

(c) Plasma concentrations

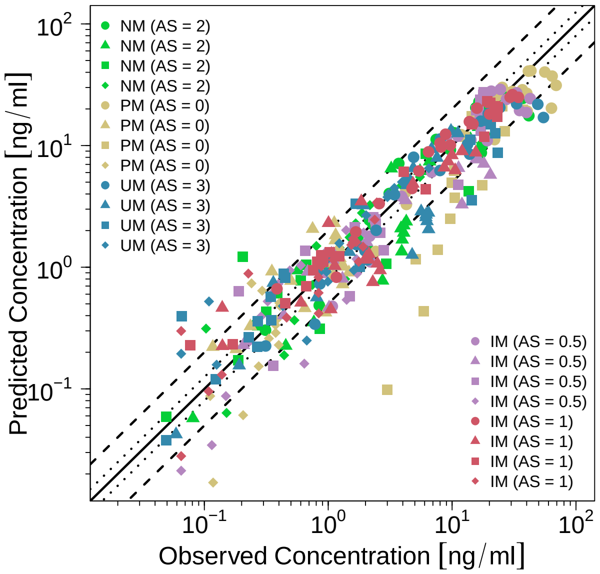

Figure S11. Predicted versus observed AUC_{last} (a), C_{max} (b) and plasma concentrations (c) of (*E*)-Clom (circles), (*E*)-4-OH-Clom (triangles), (*E*)-DE-Clom (squares) and (*E*)-4-OH-DE-Clom (diamonds) for DD(G)I scenarios with clarithromycin and paroxetine, respectively in PM, IM, NM and UM. The black solid lines mark the lines of identity. Black dotted lines indicate 1.25-fold, black dashed lines indicate 2-fold deviation. **AS**, CYP2D6 activity score; **DD(G)I**, drug-drug and drug-drug-gene interactions; **(*E*)-4-OH-Clom**, (*E*)-4-hydroxyclomiphene;**(*E*)-4-OH-DE-Clom**, (*E*)-4-hydroxy-N-desethylclomiphene; **(*E*)-Clom**, (*E*)-clomiphene; **(*E*)-DE-Clom**, (*E*)-N-desethylclomiphene; **IM**, intermediate metabolizers; **NM**, normal metabolizers, **PM**, poor metabolizers; **UM**, ultrarapid metabolizers.

(a) AUC

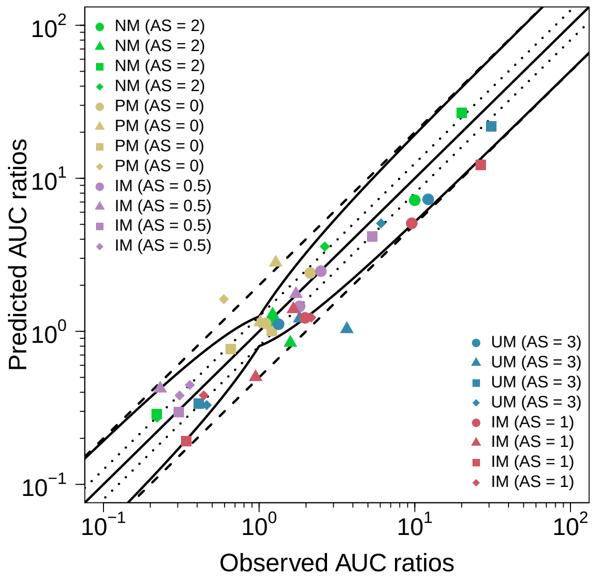

(b) C_{max}

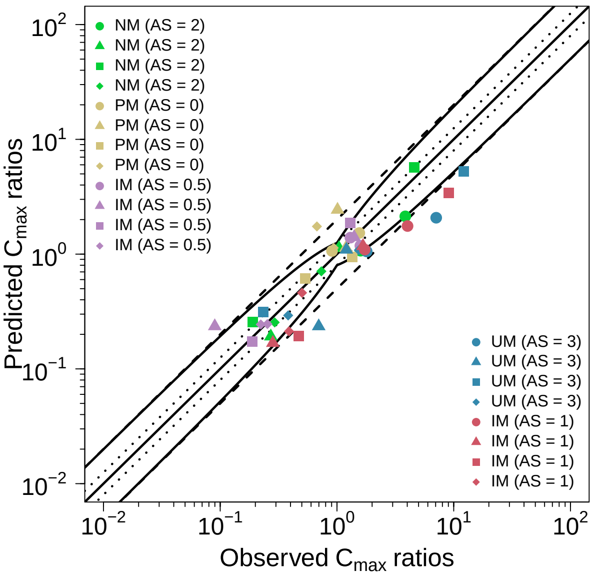

Figure S12. Predicted versus observed DD(G)I AUC_{last} (a) and C_{max} (b) ratios of (*E*)-Clom (circles), (*E*)-4-OH-Clom (triangles), (*E*)-DE-Clom (squares) and (*E*)-4-OH-DE-Clom (diamonds) in PM, IM, NM and UM. The straight black lines mark the lines of identity, the curved black lines show the limits of the predictive measure proposed by Guest et al. with 1.25-fold variability [46]. Black dotted lines indicate 1.25-fold, black dashed lines indicate 2-fold deviation. **AS**, CYP2D6 activity score; **DD(G)I**, drug-drug and drug-drug-gene interactions;**(*E*)-4-OH-Clom**, (*E*)-4-hydroxyclomiphene; **(*E*)-4-OH-DE-Clom**, (*E*)-4-hydroxy-N-desethylclomiphene; **(*E*)-Clom**, (*E*)-clomiphene; **(*E*)-DE-Clom**, (*E*)-N-desethylclomiphene; **IM**, intermediate metabolizers; **NM**, normal metabolizers, **PM**, poor metabolizers; **UM**, ultrarapid metabolizers.

S4.2.4. Renal Excretion Profiles (Linear Scale)

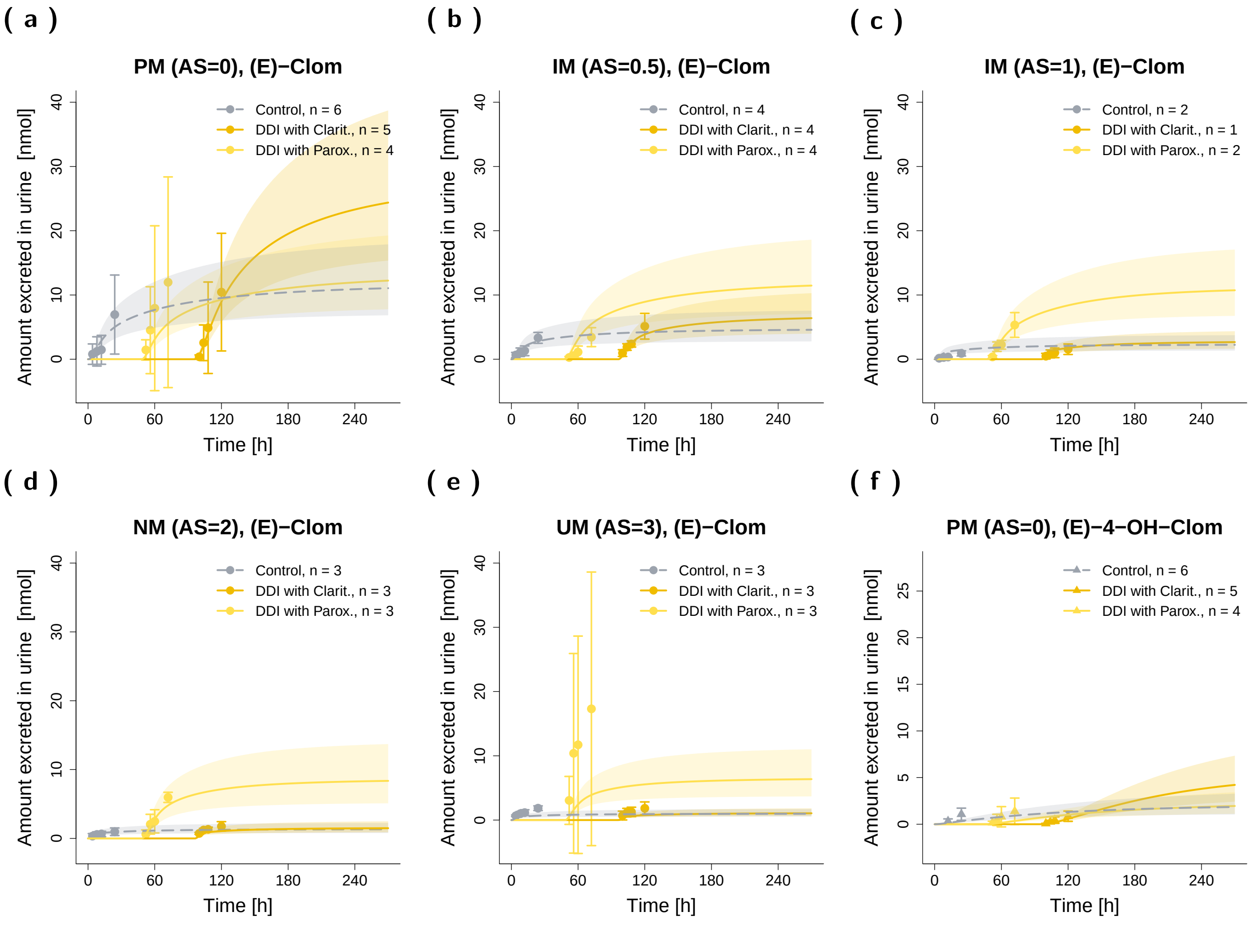

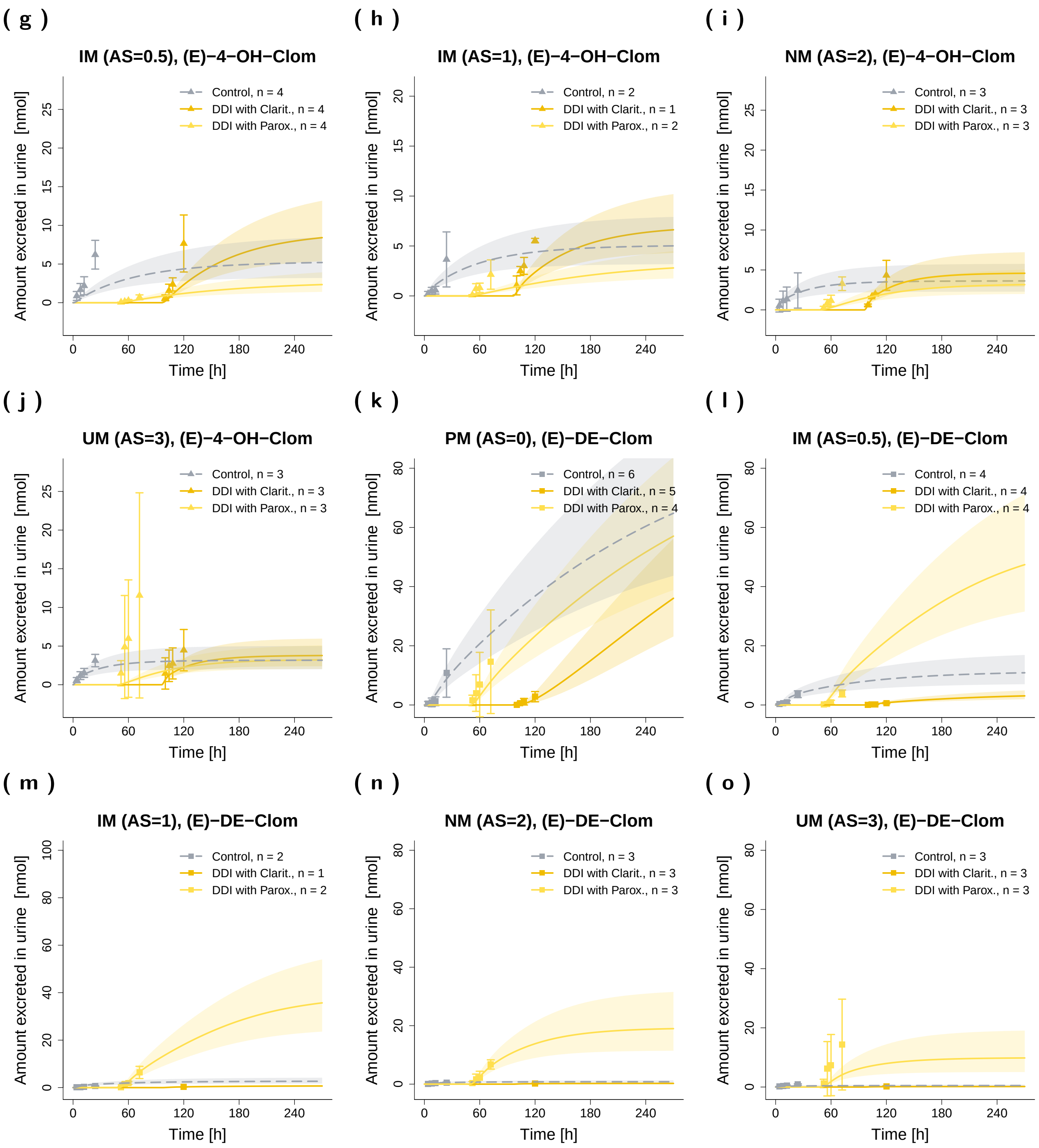

(g)
IM (AS=0.5), (E)−4−OH−Clom
Control, n = 4
DDI with Clarit., n = 4
DDI with Parox., n = 4
(h)
IM (AS=1), (E)−4−OH−Clom
Control, n = 2
DDI with Clarit., n = 1
DDI with Parox., n = 2
(i)
NM (AS=2), (E)−4−OH−Clom
Control, n = 3
DDI with Clarit., n = 3
DDI with Parox., n = 3
(j)
UM (AS=3), (E)−4−OH−Clom
Control, n = 3
DDI with Clarit., n = 3
DDI with Parox., n = 3
(k)
PM (AS=0), (E)−DE−Clom
Control, n = 6
DDI with Clarit., n = 5
DDI with Parox., n = 4
(l)
IM (AS=0.5), (E)−DE−Clom
Control, n = 4
DDI with Clarit., n = 4
DDI with Parox., n = 4
(m)
IM (AS=1), (E)−DE−Clom
Control, n = 2
DDI with Clarit., n = 1
DDI with Parox., n = 2
(n)
NM (AS=2), (E)−DE−Clom
Control, n = 3
DDI with Clarit., n = 3
DDI with Parox., n = 3
(o)
UM (AS=3), (E)−DE−Clom
Control, n = 3
DDI with Clarit., n = 3
DDI with Parox., n = 3
Amount excreted in urine [nmol]
Time [h]
0
60
120
180
240

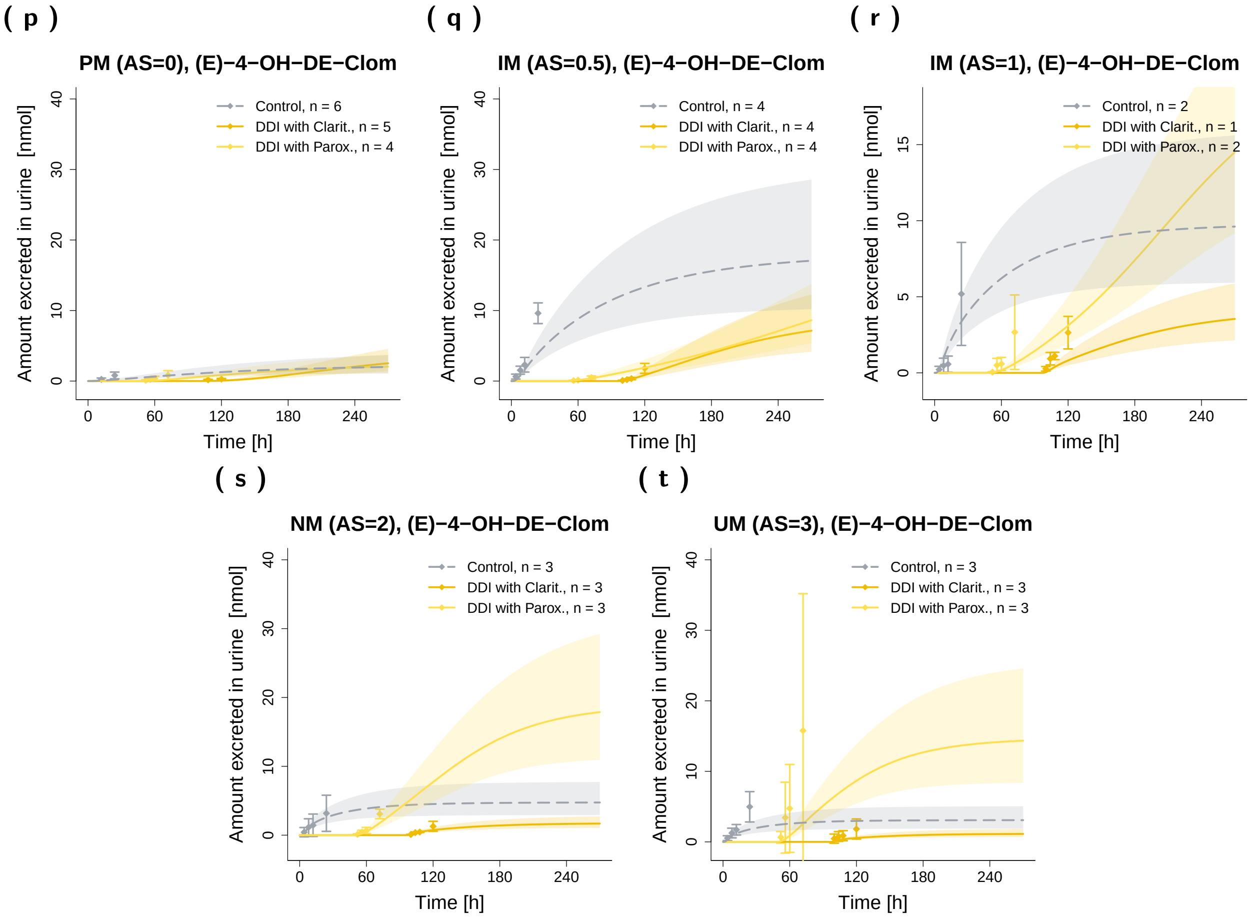

Figure S13. Predicted and observed renal excretion profiles (linear scale) of (*E*)-Clom (a–e), (*E*)-4-OH-Clom (f–j), (*E*)-DE-Clom (k–o) and (*E*)-4-OH-DE-Clom (p–t) for DD(G)I scenarios in PM, IM, NM and UM. Grey dashed lines depict the predicted geometric mean profiles in absence of clarithromycin and paroxetine (control), yellow solid lines represent the predicted geometric mean profiles in presence of paroxetine and orange solid lines represent the predicted geometric mean profiles in presence of clarithromycin (DD(G)I). The respective semitransparent areas show the geometric standard deviation of the population simulations (n=1000). Mean observed data are shown as symbols with the corresponding standard deviation. For a better visibility, DD(G)I scenarios were plotted with a time offset with t=0 at the first dose of the perpetrator drug. **AS**, CYP2D6 activity score; **Clarit.**, clarithromycin; **DD(G)I**, drug-drug and drug-drug-gene interactions; **(*E*)-4-OH-Clom**, (*E*)-4-hydroxyclomiphene; **(E)-4-OH-DE-Clom**, (*E*)-4-hydroxy-N-desethylclomiphene; **(*E*)-Clom**, (*E*)-clomiphene; **(*E*)-DE-Clom**, (*E*)-N-desethylclomiphene; **IM**, intermediate metabolizers; **n**, number of subjects; **NM**, normal metabolizers; **Parox.**, paroxetine; **PM**, poor metabolizers; **UM**, ultrarapid metabolizers.

S4.3. Quantitative PBPK Model Evaluation

S4.3.1. Mean Relative Deviation (MRD)

Table S9. Mean relative deviation (MRD) values of DGI plasma concentration predictions.

Study	Compound	MRD	Reference
PK Panel Study, PM (AS = 0)	(*E*)-4-OH-Clom	1.49	[19]
PK Panel Study, PM (AS = 0)	(*E*)-4-OH-DE-Clom	1.20	[19]
PK Panel Study, PM (AS = 0)	(*E*)-Clom	1.42	[19]
PK Panel Study, PM (AS = 0)	(*E*)-DE-Clom	1.38	[19]
PK Panel Study, IM (AS = 0.5)	(*E*)-4-OH-Clom	2.00	[19]
PK Panel Study, IM (AS = 0.5)	(*E*)-4-OH-DE-Clom	1.44	[19]
PK Panel Study, IM (AS = 0.5)	(*E*)-Clom	1.31	[19]
PK Panel Study, IM (AS = 0.5)	(*E*)-DE-Clom	2.04	[19]
PK Panel Study, IM (AS = 0.75)	(*E*)-4-OH-Clom	2.45	[19]
PK Panel Study, IM (AS = 0.75)	(*E*)-4-OH-DE-Clom	3.04	[19]
PK Panel Study, IM (AS = 0.75)	(*E*)-Clom	3.24	[19]
PK Panel Study, IM (AS = 0.75)	(*E*)-DE-Clom	5.42	[19]
PK Panel Study, IM (AS = 1)	(*E*)-4-OH-Clom	1.96	[19]
PK Panel Study, IM (AS = 1)	(*E*)-4-OH-DE-Clom	2.38	[19]
PK Panel Study, IM (AS = 1)	(*E*)-Clom	1.99	[19]
PK Panel Study, IM (AS = 1)	(*E*)-DE-Clom	2.52	[19]
PK Panel Study, NM (AS = 2)	(*E*)-4-OH-Clom	1.40	[19]
PK Panel Study, NM (AS = 2)	(*E*)-4-OH-DE-Clom	1.30	[19]
PK Panel Study, NM (AS = 2)	(*E*)-Clom	1.39	[19]
PK Panel Study, NM (AS = 2)	(*E*)-DE-Clom	1.38	[19]
PK Panel Study, UM (AS = 3)	(*E*)-4-OH-Clom	2.26	[19]
PK Panel Study, UM (AS = 3)	(*E*)-4-OH-DE-Clom	1.81	[19]
PK Panel Study, UM (AS = 3)	(*E*)-Clom	1.30	[19]
PK Panel Study, UM (AS = 3)	(*E*)-DE-Clom	1.50	[19]
Mikkelson et al. 1986	(*E*)-Clom	1.43	[1]
Miller et al. 2018	(*E*)-Clom	2.01	[4]
Study Ratioph. 1991	(*E*)-Clom	1.61	[2]
Wiehle et al. 2013 (a)	(*E*)-Clom	1.14	[3]
Wiehle et al. 2013 (b)	(*E*)-Clom	1.13	[3]
Wiehle et al. 2013 (c)	(*E*)-Clom	1.33	[3]
**Overall MRD: 1.95 (1.13–5.42)**			
**21/30 MRD ≤ 2**			

AS: CYP2D6 acitivity score, **DGI**: drug-gene interaction, **(*E*)-4-OH-Clom**: (*E*)-4-hydroxyclomiphene, **(*E*)-4-OH-DE-Clom**: (*E*)-4-hydroxy-N-desethylclomiphene, **(*E*)-Clom**: (*E*)-clomiphene, **(*E*)-DE-Clom**: (*E*)-N-desethylclomiphene, **IM**: intermediate metabolizers, **NM**: normal metabolizers, **PK**: pharmacokinetic, **PM**: poor metabolizers, **UM**: ultrarapid metabolizers, **Ratioph.**: Ratiopharm® GmbH

Table S10. Mean relative deviation (MRD) values of DD(G)I plasma concentration predictions.

Study	Compound	Perpetrator	MRD	Reference
PK Panel Study, PM (AS = 0)	(*E*)-4-OH-Clom	Clarithromycin	1.80	[19]
PK Panel Study, PM (AS = 0)	(*E*)-4-OH-Clom	Paroxetine	1.50	[19]
PK Panel Study, PM (AS = 0)	(*E*)-4-OH-DE-Clom	Clarithromycin	2.04	[19]
PK Panel Study, PM (AS = 0)	(*E*)-4-OH-DE-Clom	Paroxetine	2.18	[19]
PK Panel Study, PM (AS = 0)	(*E*)-Clom	Clarithromycin	1.72	[19]
PK Panel Study, PM (AS = 0)	(*E*)-Clom	Paroxetine	1.41	[19]
PK Panel Study, PM (AS = 0)	(*E*)-DE-Clom	Clarithromycin	4.87	[19]
PK Panel Study, PM (AS = 0)	(*E*)-DE-Clom	Paroxetine	2.04	[19]
PK Panel Study, IM (AS = 0.5)	(*E*)-4-OH-Clom	Clarithromycin	2.18	[19]
PK Panel Study, IM (AS = 0.5)	(*E*)-4-OH-Clom	Paroxetine	1.38	[19]
PK Panel Study, IM (AS = 0.5)	(*E*)-4-OH-DE-Clom	Clarithromycin	2.11	[19]
PK Panel Study, IM (AS = 0.5)	(*E*)-4-OH-DE-Clom	Paroxetine	1.73	[19]
PK Panel Study, IM (AS = 0.5)	(*E*)-Clom	Clarithromycin	1.55	[19]
PK Panel Study, IM (AS = 0.5)	(*E*)-Clom	Paroxetine	1.43	[19]
PK Panel Study, IM (AS = 0.5)	(*E*)-DE-Clom	Clarithromycin	1.92	[19]
PK Panel Study, IM (AS = 0.5)	(*E*)-DE-Clom	Paroxetine	1.53	[19]
PK Panel Study, IM (AS = 1)	(*E*)-4-OH-Clom	Clarithromycin	1.79	[19]
PK Panel Study, IM (AS = 1)	(*E*)-4-OH-Clom	Paroxetine	2.01	[19]
PK Panel Study, IM (AS = 1)	(*E*)-4-OH-DE-Clom	Clarithromycin	2.03	[19]
PK Panel Study, IM (AS = 1)	(*E*)-4-OH-DE-Clom	Paroxetine	1.38	[19]
PK Panel Study, IM (AS = 1)	(*E*)-Clom	Clarithromycin	1.30	[19]
PK Panel Study, IM (AS = 1)	(*E*)-Clom	Paroxetine	1.25	[19]
PK Panel Study, IM (AS = 1)	(*E*)-DE-Clom	Clarithromycin	1.53	[19]
PK Panel Study, IM (AS = 1)	(*E*)-DE-Clom	Paroxetine	1.29	[19]
PK Panel Study, NM (AS = 2)	(*E*)-4-OH-Clom	Clarithromycin	1.38	[19]
PK Panel Study, NM (AS = 2)	(*E*)-4-OH-Clom	Paroxetine	2.23	[19]
PK Panel Study, NM (AS = 2)	(*E*)-4-OH-DE-Clom	Clarithromycin	1.67	[19]
PK Panel Study, NM (AS = 2)	(*E*)-4-OH-DE-Clom	Paroxetine	1.52	[19]
PK Panel Study, NM (AS = 2)	(*E*)-Clom	Clarithromycin	1.44	[19]
PK Panel Study, NM (AS = 2)	(*E*)-Clom	Paroxetine	1.51	[19]
PK Panel Study, NM (AS = 2)	(*E*)-DE-Clom	Clarithromycin	1.62	[19]
PK Panel Study, NM (AS = 2)	(*E*)-DE-Clom	Paroxetine	2.19	[19]
PK Panel Study, UM (AS = 3)	(*E*)-4-OH-Clom	Clarithromycin	1.42	[19]
PK Panel Study, UM (AS = 3)	(*E*)-4-OH-Clom	Paroxetine	2.28	[19]
PK Panel Study, UM (AS = 3)	(*E*)-4-OH-DE-Clom	Clarithromycin	1.72	[19]
PK Panel Study, UM (AS = 3)	(*E*)-4-OH-DE-Clom	Paroxetine	1.71	[19]
PK Panel Study, UM (AS = 3)	(*E*)-Clom	Clarithromycin	1.46	[19]
PK Panel Study, UM (AS = 3)	(*E*)-Clom	Paroxetine	1.74	[19]
PK Panel Study, UM (AS = 3)	(*E*)-DE-Clom	Clarithromycin	1.47	[19]
PK Panel Study, UM (AS = 3)	(*E*)-DE-Clom	Paroxetine	2.38	[19]
**Overall MRD: 1.83 (1.25–4.87)**				
**28/40 MRD ≤ 2**				

AS: CYP2D6 acitivity score, **DD(G)I**: drug-drug and drug-drug-gene interactions, **(*E*)-4-OH-Clom**: (*E*)-4-hydroxyclomiphene, **(*E*)-4-OH-DE-Clom**: (*E*)-4-hydroxy-N-desethylclomiphene, **(*E*)-Clom**: (*E*)-clomiphene, **(*E*)-DE-Clom**: (*E*)-N-desethylclomiphene, **IM**: intermediate metabolizers, **NM**: normal metabolizers, **PK**: pharmacokinetic, **PM**: poor metabolizers, **UM**: ultrarapid metabolizers

S4.3.2. Geometric Mean Fold Error (GMFE)

Table S11. Geometric Mean Fold Error (GMFE) of AUC_{last} and C_{max} DGI Predictions.

		$\mathbf{AUC_{last}}$			$\mathbf{C_{max}}$			
Study	Compound	Pred $\left[\frac{ng \cdot h}{ml}\right]$	Obs $\left[\frac{ng \cdot h}{ml}\right]$	Pred/Obs	Pred $\left[\frac{ng}{ml}\right]$	Obs $\left[\frac{ng}{ml}\right]$	Pred/Obs	Reference
PK Panel Study, PM (AS = 0)	(*E*)-Clom	919.01	1095.56	0.84	27.00	44.53	0.61	[19]
PK Panel Study, PM (AS = 0)	(*E*)-4-OH-Clom	102.28	93.66	1.09	0.98	1.23	0.79	[19]
PK Panel Study, PM (AS = 0)	(*E*)-DE-Clom	3389.54	3473.88	0.98	29.59	27.34	1.08	[19]
PK Panel Study, PM (AS = 0)	(*E*)-4-OH-DE-Clom	61.68	62.33	0.99	0.47	0.44	1.09	[19]
PK Panel Study, IM (AS = 0.5)	(*E*)-Clom	401.77	422.50	0.95	20.81	26.89	0.77	[19]
PK Panel Study, IM (AS = 0.5)	(*E*)-4-OH-Clom	330.20	513.99	0.64	5.61	14.50	0.39	[19]
PK Panel Study, IM (AS = 0.5)	(*E*)-DE-Clom	741.34	446.69	1.66	14.89	14.86	1.00	[19]
PK Panel Study, IM (AS = 0.5)	(*E*)-4-OH-DE-Clom	610.91	562.68	1.09	8.39	7.63	1.10	[19]
PK Panel Study, IM (AS = 0.75)	(*E*)-Clom	344.04	136.73	2.52	19.30	9.96	1.94	[19]
PK Panel Study, IM (AS = 0.75)	(*E*)-4-OH-Clom	349.10	246.20	1.42	5.89	13.33	0.44	[19]
PK Panel Study, IM (AS = 0.75)	(*E*)-DE-Clom	575.65	102.23	5.63	12.62	6.39	1.98	[19]
PK Panel Study, IM (AS = 0.75)	(*E*)-4-OH-DE-Clom	556.31	226.19	2.46	7.89	8.01	0.98	[19]
PK Panel Study, IM (AS = 1)	(*E*)-Clom	168.98	89.54	1.89	14.93	8.53	1.75	[19]
PK Panel Study, IM (AS = 1)	(*E*)-4-OH-Clom	306.54	214.87	1.43	7.69	9.29	0.83	[19]
PK Panel Study, IM (AS = 1)	(*E*)-DE-Clom	187.42	79.09	2.37	6.88	2.59	2.66	[19]
PK Panel Study, IM (AS = 1)	(*E*)-4-OH-DE-Clom	348.35	161.70	2.15	7.62	4.92	1.55	[19]
PK Panel Study, NM (AS = 2)	(*E*)-Clom	101.66	82.93	1.23	12.20	10.82	1.13	[19]
PK Panel Study, NM (AS = 2)	(*E*)-4-OH-Clom	236.46	218.30	1.08	12.59	15.72	0.80	[19]
PK Panel Study, NM (AS = 2)	(*E*)-DE-Clom	53.16	58.47	0.91	4.09	4.50	0.91	[19]
PK Panel Study, NM (AS = 2)	(*E*)-4-OH-DE-Clom	185.45	193.74	0.96	7.81	7.49	1.04	[19]
PK Panel Study, UM (AS = 3)	(*E*)-Clom	75.53	66.21	1.14	10.74	7.72	1.39	[19]
PK Panel Study, UM (AS = 3)	(*E*)-4-OH-Clom	205.20	94.52	2.17	12.76	9.26	1.38	[19]
PK Panel Study, UM (AS = 3)	(*E*)-DE-Clom	32.98	23.91	1.38	3.05	1.93	1.58	[19]
PK Panel Study, UM (AS = 3)	(*E*)-4-OH-DE-Clom	120.44	74.07	1.63	6.01	4.21	1.43	[19]
Mikkelson et al. 1986	(*E*)-Clom	35.20	35.70	0.99	5.86	4.27	1.37	[1]
Miller et al. 2018	(*E*)-Clom	7484.01	5121.29	1.46	17.89	10.51	1.70	[4]
Study Ratioph. 1991	(*E*)-Clom	39.73	33.60	1.18	5.55	2.96	1.88	[2]
Wiehle et al. 2013 (a)	(*E*)-Clom	22.34	21.59	1.03	1.76	1.69	1.04	[3]
Wiehle et al. 2013 (b)	(*E*)-Clom	36.73	36.53	1.01	3.16	2.93	1.08	[3]
Wiehle et al. 2013 (c)	(*E*)-Clom	161.63	158.86	1.02	10.49	14.72	0.71	[3]
		**GMFE: 1.43 (1.01–5.63)**			**GMFE: 1.41 (1.00–2.66)**			

(continued)

Table S11. *continued*

Study	Compound	Pred $\left[\frac{ng \cdot h}{ml}\right]$	Obs $\left[\frac{ng \cdot h}{ml}\right]$	Pred/Obs	Pred $\left[\frac{ng}{ml}\right]$	Obs $\left[\frac{ng}{ml}\right]$	Pred/Obs	Reference
				**GMFE ≤ 2: 24/30**			**GMFE ≤ 2: 27/30**	

AS: CYP2D6 acitivity score, **DGI**: drug-gene interaction, **(*E*)-4-OH-Clom**: (*E*)-4-hydroxyclomiphene, **(*E*)-4-OH-DE-Clom**: (*E*)-4-hydroxy-N-desethylclomiphene, **(*E*)-Clom**: (*E*)-clomiphene, **(*E*)-DE-Clom**: (*E*)-N-desethylclomiphene, **IM**: intermediate metabolizers, **NM**: normal metabolizers, **Obs**: observed, **PK**: pharmacokinetic, **PM**: poor metabolizers, **Pred**: predicted, **UM**: ultrarapid metabolizers, **Ratioph.**: Ratiopharm® GmbH

Table S12. Geometric Mean Fold Error (GMFE) of DGI AUC_{last} and C_{max} ratios.

		AUC_{last} Ratio			C_{max} Ratio			
Study	Compound	Pred [1]	Obs [1]	Pred/Obs	Pred [1]	Obs [1]	Pred/Obs	Reference
PK Panel Study, PM (AS = 0)	(*E*)-Clom	9.04	13.21	0.68	2.21	4.12	0.54	[19]
PK Panel Study, PM (AS = 0)	(*E*)-4-OH-Clom	0.43	0.43	1.01	0.08	0.08	0.99	[19]
PK Panel Study, PM (AS = 0)	(*E*)-DE-Clom	63.77	59.41	1.07	7.23	6.07	1.19	[19]
PK Panel Study, PM (AS = 0)	(*E*)-4-OH-DE-Clom	0.33	0.32	1.03	0.06	0.06	1.04	[19]
PK Panel Study, IM (AS = 0.5)	(*E*)-Clom	3.95	5.09	0.78	1.71	2.49	0.69	[19]
PK Panel Study, IM (AS = 0.5)	(*E*)-4-OH-Clom	1.40	2.35	0.59	0.45	0.92	0.48	[19]
PK Panel Study, IM (AS = 0.5)	(*E*)-DE-Clom	13.95	7.64	1.83	3.64	3.30	1.10	[19]
PK Panel Study, IM (AS = 0.5)	(*E*)-4-OH-DE-Clom	3.29	2.90	1.13	1.07	1.02	1.05	[19]
PK Panel Study, IM (AS = 0.75)	(*E*)-Clom	3.38	1.65	2.05	1.58	0.92	1.72	[19]
PK Panel Study, IM (AS = 0.75)	(*E*)-4-OH-Clom	1.48	1.13	1.31	0.47	0.85	0.55	[19]
PK Panel Study, IM (AS = 0.75)	(*E*)-DE-Clom	10.83	1.75	6.19	3.08	1.42	2.17	[19]
PK Panel Study, IM (AS = 0.75)	(*E*)-4-OH-DE-Clom	3.00	1.17	2.57	1.01	1.07	0.94	[19]
PK Panel Study, IM (AS = 1)	(*E*)-Clom	1.66	1.08	1.54	1.22	0.79	1.55	[19]
PK Panel Study, IM (AS = 1)	(*E*)-4-OH-Clom	1.30	0.98	1.32	0.61	0.59	1.03	[19]
PK Panel Study, IM (AS = 1)	(*E*)-DE-Clom	3.53	1.35	2.61	1.68	0.57	2.92	[19]
PK Panel Study, IM (AS = 1)	(*E*)-4-OH-DE-Clom	1.88	0.83	2.25	0.98	0.66	1.49	[19]
PK Panel Study, UM (AS = 3)	(*E*)-Clom	0.74	0.80	0.93	0.88	0.71	1.23	[19]
PK Panel Study, UM (AS = 3)	(*E*)-4-OH-Clom	0.87	0.43	2.00	1.01	0.59	1.72	[19]
PK Panel Study, UM (AS = 3)	(*E*)-DE-Clom	0.62	0.41	1.52	0.75	0.43	1.74	[19]
PK Panel Study, UM (AS = 3)	(*E*)-4-OH-DE-Clom	0.65	0.38	1.70	0.77	0.56	1.37	[19]
		**GMFE: 1.65 (1.00–6.19)**			**GMFE: 1.46 (1.00–2.95)**			
		**GMFE ≤ 2: 14/20**			**GMFE ≤ 2: 17/20**			
		**Guest limits: 12/20**			**Guest limits: 10/20**			

AS: CYP2D6 acitivity score, **DGI**: drug-gene interaction, **(*E*)-4-OH-Clom**: (*E*)-4-hydroxyclomiphene, **(*E*)-4-OH-DE-Clom**: (*E*)-4-hydroxy-N-desethylclomiphene, **(*E*)-Clom**: (*E*)-clomiphene, **(*E*)-DE-Clom**: (*E*)-N-desethylclomiphene, **IM**: intermediate metabolizers, **NM**: normal metabolizers, **Obs**: observed, **PK**: pharmacokinetic, **PM**: poor metabolizers, **Pred**: predicted, **UM**: ultrarapid metabolizers

Table S13. Geometric Mean Fold Error (GMFE) of AUC_{last} and C_{max} DD(G)I Predictions.

Study	Compound	Perpetrator	AUC_{last}			C_{max}			Reference
			Pred $\left[\frac{ng \cdot h}{ml}\right]$	Obs $\left[\frac{ng \cdot h}{ml}\right]$	Pred/Obs	Pred $\left[\frac{ng}{ml}\right]$	Obs $\left[\frac{ng}{ml}\right]$	Pred/Obs	
PK Panel Study, PM (AS = 0)	(*E*)-Clom	Clarithromycin	2211.99	2332.83	0.95	41.25	69.18	0.60	[19]
PK Panel Study, PM (AS = 0)	(*E*)-4-OH-Clom	Clarithromycin	287.69	119.75	2.40	2.38	1.24	1.91	[19]
PK Panel Study, PM (AS = 0)	(*E*)-DE-Clom	Clarithromycin	2592.38	2282.03	1.14	18.17	14.69	1.24	[19]
PK Panel Study, PM (AS = 0)	(*E*)-4-OH-DE-Clom	Clarithromycin	100.11	36.96	2.71	0.82	0.29	2.81	[19]
PK Panel Study, IM (AS = 0.5)	(*E*)-Clom	Clarithromycin	585.91	769.47	0.76	24.64	42.67	0.58	[19]
PK Panel Study, IM (AS = 0.5)	(*E*)-4-OH-Clom	Clarithromycin	578.95	885.93	0.65	7.88	20.35	0.39	[19]
PK Panel Study, IM (AS = 0.5)	(*E*)-DE-Clom	Clarithromycin	219.84	135.87	1.62	2.58	2.79	0.92	[19]
PK Panel Study, IM (AS = 0.5)	(*E*)-4-OH-DE-Clom	Clarithromycin	272.46	201.78	1.35	2.06	1.93	1.07	[19]
PK Panel Study, IM (AS = 1)	(*E*)-Clom	Clarithromycin	207.03	176.77	1.17	16.37	14.74	1.11	[19]
PK Panel Study, IM (AS = 1)	(*E*)-4-OH-Clom	Clarithromycin	427.82	356.60	1.20	9.05	15.53	0.58	[19]
PK Panel Study, IM (AS = 1)	(*E*)-DE-Clom	Clarithromycin	35.98	26.96	1.33	1.33	1.22	1.09	[19]
PK Panel Study, IM (AS = 1)	(*E*)-4-OH-DE-Clom	Clarithromycin	132.72	71.32	1.86	1.62	1.91	0.85	[19]
PK Panel Study, NM (AS = 2)	(*E*)-Clom	Clarithromycin	117.25	100.98	1.16	13.07	17.22	0.76	[19]
PK Panel Study, NM (AS = 2)	(*E*)-4-OH-Clom	Clarithromycin	304.50	266.53	1.14	14.21	16.36	0.87	[19]
PK Panel Study, NM (AS = 2)	(*E*)-DE-Clom	Clarithromycin	15.33	12.85	1.19	1.05	0.85	1.23	[19]
PK Panel Study, NM (AS = 2)	(*E*)-4-OH-DE-Clom	Clarithromycin	50.59	42.79	1.18	1.98	2.19	0.91	[19]
PK Panel Study, UM (AS = 3)	(*E*)-Clom	Clarithromycin	84.09	88.29	0.95	11.34	13.87	0.82	[19]
PK Panel Study, UM (AS = 3)	(*E*)-4-OH-Clom	Clarithromycin	248.86	172.19	1.45	13.96	11.16	1.25	[19]
PK Panel Study, UM (AS = 3)	(*E*)-DE-Clom	Clarithromycin	11.10	9.79	1.13	0.96	0.45	2.11	[19]
PK Panel Study, UM (AS = 3)	(*E*)-4-OH-DE-Clom	Clarithromycin	35.43	30.40	1.17	1.76	1.61	1.09	[19]
PK Panel Study, PM (AS = 0)	(*E*)-Clom	Paroxetine	1035.07	1204.78	0.86	28.75	40.39	0.71	[19]
PK Panel Study, PM (AS = 0)	(*E*)-4-OH-Clom	Paroxetine	117.15	95.18	1.23	1.11	1.47	0.76	[19]
PK Panel Study, PM (AS = 0)	(*E*)-DE-Clom	Paroxetine	3405.95	4195.92	0.81	27.95	37.12	0.75	[19]
PK Panel Study, PM (AS = 0)	(*E*)-4-OH-DE-Clom	Paroxetine	69.53	63.01	1.10	0.54	0.41	1.31	[19]
PK Panel Study, IM (AS = 0.5)	(*E*)-Clom	Paroxetine	993.64	1053.60	0.94	28.98	35.05	0.83	[19]
PK Panel Study, IM (AS = 0.5)	(*E*)-4-OH-Clom	Paroxetine	139.64	119.04	1.17	1.32	1.30	1.01	[19]
PK Panel Study, IM (AS = 0.5)	(*E*)-DE-Clom	Paroxetine	3094.26	2384.31	1.30	27.70	19.23	1.44	[19]
PK Panel Study, IM (AS = 0.5)	(*E*)-4-OH-DE-Clom	Paroxetine	232.51	173.56	1.34	2.05	1.70	1.20	[19]
PK Panel Study, IM (AS = 1)	(*E*)-Clom	Paroxetine	858.97	855.99	1.00	26.24	34.32	0.76	[19]
PK Panel Study, IM (AS = 1)	(*E*)-4-OH-Clom	Paroxetine	153.97	204.03	0.75	1.30	2.62	0.49	[19]
PK Panel Study, IM (AS = 1)	(*E*)-DE-Clom	Paroxetine	2288.71	2104.81	1.09	23.38	23.47	1.00	[19]
PK Panel Study, IM (AS = 1)	(*E*)-4-OH-DE-Clom	Paroxetine	428.74	349.83	1.23	3.51	2.47	1.42	[19]
PK Panel Study, NM (AS = 2)	(*E*)-Clom	Paroxetine	731.17	828.75	0.88	26.03	41.65	0.63	[19]

(continued)

Table S13. *continued*

Study	Compound	Perpetrator	Pred $\left[\frac{ng \cdot h}{ml}\right]$	Obs $\left[\frac{ng \cdot h}{ml}\right]$	Pred/Obs	Pred $\left[\frac{ng}{ml}\right]$	Obs $\left[\frac{ng}{ml}\right]$	Pred/Obs	Reference
PK Panel Study, NM (AS = 2)	(*E*)-4-OH-Clom	Paroxetine	199.16	346.58	0.57	2.41	4.27	0.57	[19]
PK Panel Study, NM (AS = 2)	(*E*)-DE-Clom	Paroxetine	1421.08	1170.81	1.21	23.33	20.66	1.13	[19]
PK Panel Study, NM (AS = 2)	(*E*)-4-OH-DE-Clom	Paroxetine	664.56	511.87	1.30	5.53	5.50	1.01	[19]
PK Panel Study, UM (AS = 3)	(*E*)-Clom	Paroxetine	550.74	806.88	0.68	22.29	54.63	0.41	[19]
PK Panel Study, UM (AS = 3)	(*E*)-4-OH-Clom	Paroxetine	212.08	345.90	0.61	3.00	6.46	0.46	[19]
PK Panel Study, UM (AS = 3)	(*E*)-DE-Clom	Paroxetine	722.15	739.59	0.98	16.09	23.37	0.69	[19]
PK Panel Study, UM (AS = 3)	(*E*)-4-OH-DE-Clom	Paroxetine	545.59	400.60	1.36	6.51	6.49	1.00	[19]
					**GMFE: 1.30 (1.00–2.71)**			**GMFE: 1.40 (1.00–2.83)**	
					**GMFE ≤ 2: 38/40**			**GMFE ≤ 2: 34/40**	

AS: CYP2D6 acitivity score, **DD(G)I**: drug-drug and drug-drug-gene interactions, **(*E*)-4-OH-Clom**: (*E*)-4-hydroxyclomiphene, **(*E*)-4-OH-DE-Clom**: (*E*)-4-hydroxy-N-desethylclomiphene, **(*E*)-Clom**: (*E*)-clomiphene, **(*E*)-DE-Clom**: (*E*)-N-desethylclomiphene, **IM**: intermediate metabolizers, **NM**: normal metabolizers, **Obs**: observed, **PK**: pharmacokinetic, **PM**: poor metabolizers, **Pred**: predicted, **UM**: ultrarapid metabolizers

Table S14. Geometric Mean Fold Error (GMFE) of DD(G)I AUC_{last} and C_{max} ratios.

Study	Compound	Perpetrator	AUC_{last} Ratio			C_{max} Ratio			Reference
			Pred [1]	Obs [1]	Pred/Obs	Pred [1]	Obs [1]	Pred/Obs	
PK Panel Study, PM (AS = 0)	(*E*)-Clom	Clarithromycin	2.41	2.13	1.13	1.53	1.55	0.98	[19]
PK Panel Study, PM (AS = 0)	(*E*)-4-OH-Clom	Clarithromycin	2.81	1.28	2.20	2.44	1.01	2.41	[19]
PK Panel Study, PM (AS = 0)	(*E*)-DE-Clom	Clarithromycin	0.76	0.66	1.16	0.61	0.54	1.14	[19]
PK Panel Study, PM (AS = 0)	(*E*)-4-OH-DE-Clom	Clarithromycin	1.62	0.59	2.74	1.74	0.67	2.59	[19]
PK Panel Study, IM (AS = 0.5)	(*E*)-Clom	Clarithromycin	1.46	1.82	0.80	1.18	1.59	0.75	[19]
PK Panel Study, IM (AS = 0.5)	(*E*)-4-OH-Clom	Clarithromycin	1.75	1.72	1.02	1.41	1.40	1.00	[19]
PK Panel Study, IM (AS = 0.5)	(*E*)-DE-Clom	Clarithromycin	0.30	0.30	0.97	0.17	0.19	0.92	[19]
PK Panel Study, IM (AS = 0.5)	(*E*)-4-OH-DE-Clom	Clarithromycin	0.45	0.36	1.24	0.25	0.25	0.97	[19]
PK Panel Study, IM (AS = 1)	(*E*)-Clom	Clarithromycin	1.23	1.97	0.62	1.10	1.73	0.63	[19]
PK Panel Study, IM (AS = 1)	(*E*)-4-OH-Clom	Clarithromycin	1.40	1.66	0.84	1.18	1.67	0.70	[19]
PK Panel Study, IM (AS = 1)	(*E*)-DE-Clom	Clarithromycin	0.19	0.34	0.56	0.19	0.47	0.41	[19]
PK Panel Study, IM (AS = 1)	(*E*)-4-OH-DE-Clom	Clarithromycin	0.38	0.44	0.86	0.21	0.39	0.55	[19]
PK Panel Study, NM (AS = 2)	(*E*)-Clom	Clarithromycin	1.15	1.22	0.95	1.07	1.59	0.67	[19]
PK Panel Study, NM (AS = 2)	(*E*)-4-OH-Clom	Clarithromycin	1.29	1.22	1.05	1.13	1.04	1.08	[19]
PK Panel Study, NM (AS = 2)	(*E*)-DE-Clom	Clarithromycin	0.29	0.22	1.31	0.26	0.19	1.36	[19]
PK Panel Study, NM (AS = 2)	(*E*)-4-OH-DE-Clom	Clarithromycin	0.27	0.22	1.24	0.25	0.29	0.87	[19]
PK Panel Study, UM (AS = 3)	(*E*)-Clom	Clarithromycin	1.11	1.33	0.84	1.06	1.80	0.59	[19]
PK Panel Study, UM (AS = 3)	(*E*)-4-OH-Clom	Clarithromycin	1.21	1.82	0.67	1.09	1.20	0.91	[19]
PK Panel Study, UM (AS = 3)	(*E*)-DE-Clom	Clarithromycin	0.34	0.41	0.82	0.31	0.23	1.33	[19]
PK Panel Study, UM (AS = 3)	(*E*)-4-OH-DE-Clom	Clarithromycin	0.33	0.46	0.72	0.29	0.38	0.77	[19]
PK Panel Study, PM (AS = 0)	(*E*)-Clom	Paroxetine	1.13	1.10	1.02	1.07	0.91	1.17	[19]
PK Panel Study, PM (AS = 0)	(*E*)-4-OH-Clom	Paroxetine	1.15	1.02	1.13	1.13	1.19	0.95	[19]
PK Panel Study, PM (AS = 0)	(*E*)-DE-Clom	Paroxetine	1.00	1.21	0.83	0.94	1.36	0.70	[19]
PK Panel Study, PM (AS = 0)	(*E*)-4-OH-DE-Clom	Paroxetine	1.13	1.01	1.12	1.13	0.94	1.21	[19]
PK Panel Study, IM (AS = 0.5)	(*E*)-Clom	Paroxetine	2.47	2.49	0.99	1.39	1.30	1.07	[19]
PK Panel Study, IM (AS = 0.5)	(*E*)-4-OH-Clom	Paroxetine	0.42	0.23	1.83	0.24	0.09	2.62	[19]
PK Panel Study, IM (AS = 0.5)	(*E*)-DE-Clom	Paroxetine	4.17	5.34	0.78	1.86	1.29	1.44	[19]
PK Panel Study, IM (AS = 0.5)	(*E*)-4-OH-DE-Clom	Paroxetine	0.38	0.31	1.23	0.24	0.22	1.10	[19]
PK Panel Study, IM (AS = 1)	(*E*)-Clom	Paroxetine	5.08	9.56	0.53	1.76	4.02	0.44	[19]
PK Panel Study, IM (AS = 1)	(*E*)-4-OH-Clom	Paroxetine	0.50	0.95	0.53	0.17	0.28	0.60	[19]
PK Panel Study, IM (AS = 1)	(*E*)-DE-Clom	Paroxetine	12.21	26.61	0.46	3.40	9.07	0.37	[19]
PK Panel Study, IM (AS = 1)	(*E*)-4-OH-DE-Clom	Paroxetine	1.23	2.16	0.57	0.46	0.50	0.92	[19]
PK Panel Study, NM (AS = 2)	(*E*)-Clom	Paroxetine	7.19	9.99	0.72	2.13	3.85	0.55	[19]

(continued)

Table S14. *continued*

Study	Compound	Perpetrator	Pred [1]	Obs [1]	Pred/Obs	Pred [1]	Obs [1]	Pred/Obs	Reference
PK Panel Study, NM (AS = 2)	(*E*)-4-OH-Clom	Paroxetine	0.84	1.59	0.53	0.19	0.27	0.71	[19]
PK Panel Study, NM (AS = 2)	(*E*)-DE-Clom	Paroxetine	26.73	20.02	1.34	5.70	4.59	1.24	[19]
PK Panel Study, NM (AS = 2)	(*E*)-4-OH-DE-Clom	Paroxetine	3.58	2.64	1.36	0.71	0.74	0.96	[19]
PK Panel Study, UM (AS = 3)	(*E*)-Clom	Paroxetine	7.29	12.19	0.60	2.08	7.08	0.29	[19]
PK Panel Study, UM (AS = 3)	(*E*)-4-OH-Clom	Paroxetine	1.03	3.66	0.28	0.24	0.70	0.34	[19]
PK Panel Study, UM (AS = 3)	(*E*)-DE-Clom	Paroxetine	21.90	30.93	0.71	5.27	12.14	0.43	[19]
PK Panel Study, UM (AS = 3)	(*E*)-4-OH-DE-Clom	Paroxetine	5.08	6.07	0.84	1.08	1.54	0.70	[19]
					**GMFE: 1.40 (1.00–3.55)**			**GMFE: 1.50 (1.00–3.40)**	
					**GMFE ≤ 2: 36/40**			**GMFE ≤ 2: 31/40**	
					**Guest limits: 29/40**			**Guest limits: 23/40**	

AS: CYP2D6 acitivity score, **DD(G)I**: drug-drug and drug-drug-gene interactions, **(*E*)-4-OH-Clom**: (*E*)-4-hydroxyclomiphene, **(*E*)-4-OH-DE-Clom**: (*E*)-4-hydroxy-N-desethylclomiphene, **(*E*)-Clom**: (*E*)-clomiphene, **(*E*)-DE-Clom**: (*E*)-N-desethylclomiphene, **IM**: intermediate metabolizers, **NM**: normal metabolizers, **Obs**: observed, **PK**: pharmacokinetic, **PM**: poor metabolizers, **Pred**: predicted, **UM**: ultrarapid metabolizers

S4.4. Local Sensitivity Analysis

S4.4.1. Mathematical Implementation

A sensitivity analysis of the developed model was conducted to explore the impact of single parameter changes (local sensitivity analysis) on the predicted AUC_{inf}. According to Equation S4, the relative change of AUC_{inf} after oral application of a single dose of 100 mg clomiphene citrate to the relative variation of model input parameters was calculated. All optimized parameters as well as parameters that might have a strong impact because of calculation methods employed in the model (e.g., lipophilicity) were integrated in the sensitivity analysis and a relative perturbation of 10% was used.

$$S = \frac{\Delta AUC_{inf}}{\Delta p} \cdot \frac{p}{AUC_{inf}} \tag{S4}$$

S is the sensitivity of the AUC_{inf} to the examined model parameter, ΔAUC_{inf} is the change of the AUC_{inf}, AUC_{inf} represents the simulated AUC_{inf} with the original parameter value, p is the original model parameter value and Δp the variation of the model parameter value. A sensitivity value of +1.0 signifies that a 10% increase of the examined parameter causes a 10% increase of the simulated AUC_{inf}.

S4.4.2. Results of the Sensitivity Analysis

(a) Sensitivity Analysis (E)-Clom

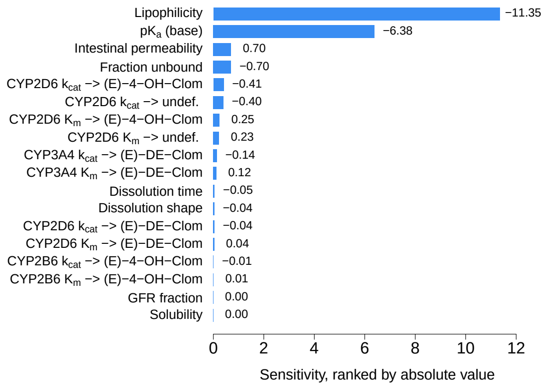

(b) Sensitivity Analysis (E)-4-OH-Clom

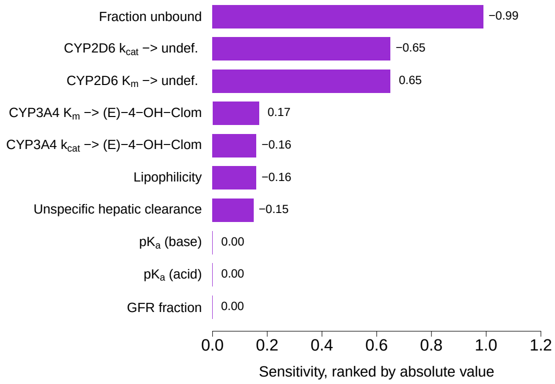

(c) Sensitivity Analysis (E)-Clom

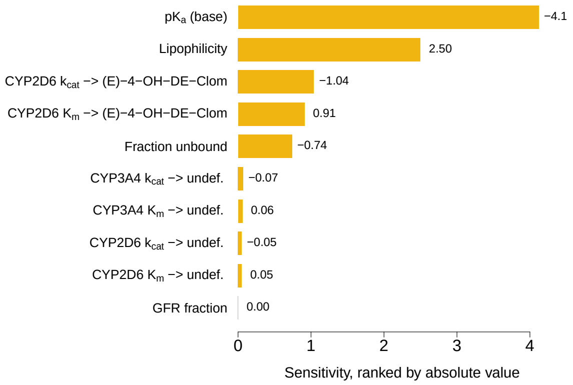

(d) Sensitivity Analysis (E)-4-OH-Clom

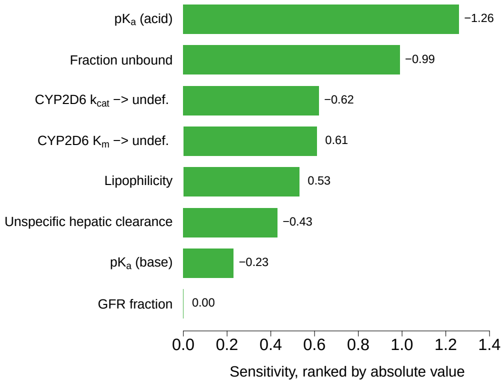

Figure S14. Sensitivity analysis of the PBPK model for (*E*)-Clom, (*E*)-4-OH-Clom, (*E*)-DE-Clom and (*E*)-4-OH-DE-Clom. **CYP**, cytochrome P450; **(*E*)-4-OH-Clom**, (*E*)-4-hydroxyclomiphene; **(*E*)-4-OH-DE-Clom**, (*E*)-4-hydroxy-N-desethylclomiphene; **(*E*)-Clom**, (*E*)-clomiphene; **(*E*)-DE-Clom**, (*E*)-N-desethylclomiphene; **GFR**, glomerular filtration rate; $\mathbf{k_{cat}}$, catalytic rate constant; $\mathbf{K_m}$, Michaelis Menten constant; $\mathbf{pK_a}$, acid dissociation constant; **undef.**, undefined metabolite.

S5. Molecular Structures

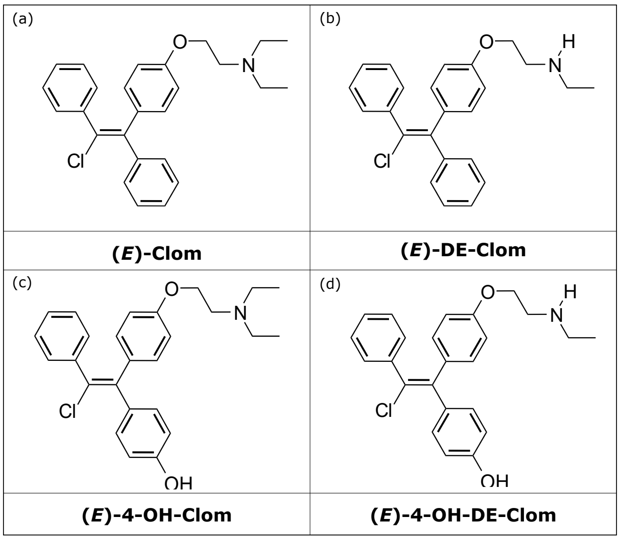

Figure S15. Molecular structures of (*E*)-Clom (a) and its metabolites (*E*)-DE-Clom (b), (*E*)-4-OH-Clom (c) and (*E*)-4-OH-DE-Clom (d). (*E*)-4-OH-Clom, (*E*)-4-hydroxyclomiphene; **(*E*)-4-OH-DE-Clom**, (*E*)-4-hydroxy-N-desethylclomiphene; **(*E*)-Clom**, (*E*)-clomiphene; **(*E*)-DE-Clom**, (*E*)-N-desethylclomiphene.

References

[1] Mikkelson TJ, Kroboth PD, Cameron WJ, Dittert LW, Chungi V, Manberg PJ (1986) Single-dose pharmacokinetics of clomiphene citrate in normal volunteers**Supported by a grant from Serono Laboratories, Inc., Randolph, Massachusetts. Fertility and Sterility 46(3):392–396

[2] Ratiopharm® GmbH (2016) Clomifen-ratiopharm® 50 mg Tabletten (Study 1991). https://www.ratiopharm.de/produkte/details/praeparate/praeparatedaten/detail/pzn-3884844.html, available online (accessed: 2021-11-23)

[3] Wiehle R, Cunningham GR, Pitteloud N, Wike J, Hsu K, Fontenot GK, Rosner M, Dwyer A, Podolski J (2013) Testosterone Restoration by Enclomiphene Citrate in Men with Secondary Hypogonadism: Pharmacodynamics and Pharmacokinetics. BJU international 112(8):1188–1200

[4] Miller GD, Moore C, Nair V, Hill B, Willick SE, Rogol AD, Eichner D (2019) Hypothalamic-Pituitary-Testicular Axis Effects and Urinary Detection Following Clomiphene Administration in Males. The Journal of clinical endocrinology and metabolism 104(3):906–914

[5] van der Lee M, Allard WG, Vossen RHAM, Baak-Pablo RF, Menafra R, Deiman BALM, Deenen MJ, Neven P, Johansson I, Gastaldello S, Ingelman-Sundberg M, Guchelaar HJ, Swen JJ, Anvar SY (2021) Toward predicting CYP2D6-mediated variable drug response from CYP2D6 gene sequencing data. Science translational medicine 13(603)

[6] Valentin J (2002) Basic anatomical and physiological data for use in radiological protection: reference values. A report of age- and gender-related differences in the anatomical and physiological characteristics of reference individuals. ICRP Publication 89. Annals of the ICRP 32(3-4):5–265

[7] Open Systems Pharmacology Suite Community (2018) PK-Sim® Ontogeny Database Documentation, Version 7.3. https://github.com/Open-Systems-Pharmacology/OSPSuite.Documentation/blob/master/PK-SimOntogenyDatabaseVersion7.3.pdf, available online (accessed: 2020-03-25)

[8] National Center for Health Statistics (1997) Third National Health and Nutrition Examination Survey (NHANES III). Tech. rep., Hyattsville, MD 20782

[9] Willmann S, Höhn K, Edginton A, Sevestre M, Solodenko J, Weiss W, Lippert J, Schmitt W (2007) Development of a physiology-based whole-body population model for assessing the influence of individual variability on the pharmacokinetics of drugs. Journal of pharmacokinetics and pharmacodynamics 34(3):401–31

[10] Rodrigues AD (1999) Integrated cytochrome P450 reaction phenotyping: attempting to bridge the gap between cDNA-expressed cytochromes P450 and native human liver microsomes. Biochemical pharmacology 57(5):465–80

[11] Nishimura M, Yaguti H, Yoshitsugu H, Naito S, Satoh T (2003) Tissue distribution of mRNA expression of human cytochrome P450 isoforms assessed by high-sensitivity real-time reverse transcription PCR. Yakugaku zasshi : Journal of the Pharmaceutical Society of Japan 123(5):369–75

[12] Rowland Yeo K, Walsky RL, Jamei M, Rostami-Hodjegan A, Tucker GT (2011) Prediction of time-dependent CYP3A4 drug-drug interactions by physiologically based pharmacokinetic modelling: impact of inactivation parameters and enzyme turnover. European journal of pharmaceutical sciences : official journal of the European Federation for Pharmaceutical Sciences 43(3):160–73

[13] Greenblatt DJ, von Moltke LL, Harmatz JS, Chen G, Weemhoff JL, Jen C, Kelley CJ, LeDuc BW, Zinny MA (2003) Time course of recovery of cytochrome p450 3A function after single doses of grapefruit juice. Clinical pharmacology and therapeutics 74(2):121–9

[14] Tsamandouras N, Rostami-Hodjegan A, Aarons L (2015) Combining the 'bottom up' and 'top down' approaches in pharmacokinetic modelling: fitting PBPK models to observed clinical data. British journal of clinical pharmacology 79(1):48–55

[15] Austin RP, Barton P, Cockroft SL, Wenlock MC, Riley RJ (2002) The influence of nonspecific microsomal binding on apparent intrinsic clearance, and its prediction from physicochemical properties. Drug metabolism and disposition: the biological fate of chemicals 30(12):1497–503

[16] Obach RS (1997) Nonspecific binding to microsomes: impact on scale-up of in vitro intrinsic clearance to hepatic clearance as assessed through examination of warfarin, imipramine, and propranolol. Drug metabolism and disposition: the biological fate of chemicals 25(12):1359–69

[17] Mürdter TE, Kerb R, Turpeinen M, Schroth W, Ganchev B, Böhmer GM, Igel S, Schaeffeler E, Zanger U, Brauch H, Schwab M (2012) Genetic polymorphism of cytochrome P450 2D6 determines oestrogen receptor activity of the major infertility drug clomiphene via its active metabolites. Human molecular genetics 21(5):1145–54

[18] Ganchev B (2014) Charakterisierung der metabolischen Bioaktivierung des Clomifens unter besonderer Berücksichtigung genetischer Polymorphismen. PhD thesis, Eberhard Karls University Tübingen

[19] Kröner P (2018) Hydroxylierte Metaboliten des Clomifens : in vitro und in vivo Untersuchungen zur Bildung, Aktivität und Konjugation. PhD thesis, Eberhard-Karls-University Tübingen

[20] Mazzarino M, Biava M, de la Torre X, Fiacco I, Botrè F (2013) Characterization of the biotransformation pathways of clomiphene, tamoxifen and toremifene as assessed by LC-MS/(MS) following in vitro and excretion studies. Analytical and bioanalytical chemistry 405(16):5467–87

[21] Watanabe R, Esaki T, Kawashima H, Natsume-Kitatani Y, Nagao C, Ohashi R, Mizuguchi K (2018) Predicting Fraction Unbound in Human Plasma from Chemical Structure: Improved Accuracy in the Low Value Ranges. Molecular pharmaceutics 15(11):5302–5311

[22] Siramshetty VB, Grishagin I, Nguyen cT, Peryea T, Skovpen Y, Stroganov O, Katzel D, Sheils T, Jadhav A, Mathé EA, Southall NT (2022) NCATS Inxight Drugs: a comprehensive and curated portal for translational research. Nucleic acids research 50(D1):D1307–D1316

[23] Developed by ChemAxon (2009) (http://www.chemaxon.com), chemicalize was used for prediction of (E)-clomiphene properties. https://chemicalize.com/, available online (accessed: 2021-08-09)

[24] Das P, Prajapati M, Maity A (2020) Study of equilibrium solubility of Clomiphene Citrate as model compound by Saturation orbital shake flask method 3(4):843–847

[25] Smith DA, Dalvie D (2012) Why do metabolites circulate? Xenobiotica; the fate of foreign compounds in biological systems 42(1):107–26

[26] Wishart DS, Knox C, Guo AC, Shrivastava S, Hassanali M, Stothard P, Chang Z, Woolsey J (2006) DrugBank: a comprehensive resource for in silico drug discovery and exploration. Nucleic acids research 34(Database issue):D668–72

[27] Güngör S, Delgado-Charro MB, Masini-Etévé V, Potts RO, Guy RH (2013) Transdermal flux predictions for selected selective oestrogen receptor modulators (SERMs): comparison with experimental results. Journal of controlled release : official journal of the Controlled Release Society 172(3):601–6

[28] Schmitt W (2008) General approach for the calculation of tissue to plasma partition coefficients. Toxicology in vitro : an international journal published in association with BIBRA 22(2):457–67

[29] Kawai R, Lemaire M, Steimer JL, Bruelisauer A, Niederberger W, Rowland M (1994) Physiologically based pharmacokinetic study on a cyclosporin derivative, SDZ IMM 125. Journal of pharmacokinetics and biopharmaceutics 22(5):327–65

[30] Developed by ChemAxon (2009) (http://www.chemaxon.com), chemicalize was used for prediction of (E)-N-desethylclomiphene properties. https://chemicalize.com/, available online (accessed: 2021-08-09)

[31] Kim S, Chen J, Cheng T, Gindulyte A, He J, He S, Li Q, Shoemaker BA, Thiessen PA, Yu B, Zaslavsky L, Zhang J, Bolton EE (2021) PubChem in 2021: new data content and improved web interfaces. Nucleic acids research 49(D1):D1388–D1395

[32] Rodgers T, Leahy D, Rowland M (2005) Physiologically based pharmacokinetic modeling 1: predicting the tissue distribution of moderate-to-strong bases. Journal of pharmaceutical sciences 94(6):1259–76

[33] Rodgers T, Rowland M (2006) Physiologically based pharmacokinetic modelling 2: predicting the tissue distribution of acids, very weak bases, neutrals and zwitterions. Journal of pharmaceutical sciences 95(6):1238–57

[34] Developed by ChemAxon (2009) (http://www.chemaxon.com), chemicalize was used for prediction of (E)-4-hydroxyclomiphene properties. https://chemicalize.com/, available online (accessed: 2021-08-09)

[35] European Medicines Agency (2018) Assessment Report EnCyzix. https://www.ema.europa.eu/en/documents/assessment-report/encyzix-epar-public-assessment-report_en.pdf, available online (accessed: 2021-08-12)

[36] Berezhkovskiy LM (2004) Volume of distribution at steady state for a linear pharmacokinetic system with peripheral elimination. Journal of pharmaceutical sciences 93(6):1628–40

[37] Open Systems Pharmacology Suite Community (2021) Open Systems Pharmacology Suite Manual. https://raw.githubusercontent.com/Open-Systems-Pharmacology/OSPSuite.Documentation/master/OpenSystemsPharmacologySuite.pdf, available online (accessed: 2022-02-19)

[38] Developed by ChemAxon (2009) (http://www.chemaxon.com), chemicalize was used for prediction of (E)-4-hydroxy-N-desethylclomiphene properties. https://chemicalize.com/, available online (accessed: 2021-08-09)

[39] T'jollyn H, Snoeys J, Vermeulen A, Michelet R, Cuyckens F, Mannens G, Van Peer A, Annaert P, Allegaert K, Van Bocxlaer J, Boussery K (2015) Physiologically Based Pharmacokinetic Predictions of Tramadol Exposure Throughout Pediatric Life: an Analysis of the Different Clearance Contributors with Emphasis on CYP2D6 Maturation. The AAPS journal 17(6):1376–87

[40] United States Pharmacopeial Convention (2006) United States Pharmacopeia and National Formulary (USP 29-NF 24). p 553, http://www.pharmacopeia.cn/v29240/usp29nf24s0_m18490.html, available online (accessed: 2021-08-24)

[41] Beal SL (2001) Ways to fit a PK model with some data below the quantification limit. Journal of pharmacokinetics and pharmacodynamics 28(5):481–504

[42] Obach RS, Walsky RL, Venkatakrishnan K (2007) Mechanism-based inactivation of human cytochrome p450 enzymes and the prediction of drug-drug interactions. Drug metabolism and disposition: the biological fate of chemicals 35(2):246–55

[43] Hanke N, Frechen S, Moj D, Britz H, Eissing T, Wendl T, Lehr T (2018) PBPK Models for CYP3A4 and P-gp DDI Prediction: A Modeling Network of Rifampicin, Itraconazole, Clarithromycin, Midazolam, Alfentanil, and Digoxin. CPT: pharmacometrics & systems pharmacology 7(10):647–659

[44] Rüdesheim S, Selzer D, Mürdter T, Igel S, Kerb R, Schwab M, Lehr T (2022) Physiologically Based Pharmacokinetic Modeling to Describe the CYP2D6 Activity Score-Dependent Metabolism of Paroxetine, Atomoxetine and Risperidone. Pharmaceutics 14(8):1734

[45] Guest EJ, Aarons L, Houston JB, Rostami-Hodjegan A, Galetin A (2011) Critique of the two-fold measure of prediction success for ratios: application for the assessment of drug-drug interactions. Drug metabolism and disposition: the biological fate of chemicals 39(2):170–3
